# Flash Communication: Reactivity of Bis(phosphine) Ligands with Dicobalt Octacarbonyl

**DOI:** 10.1021/acs.organomet.6c00176

**Published:** 2026-07-17

**Authors:** Rowan M. Bailey, Tsz-Kan Ma, Dominykas Dautoras, Benedek Stadler, Álvaro García-Romero, Andrew J. P. White, James A. Bull, Mark R. Crimmin, Philip W. Miller

**Affiliations:** Department of Chemistry, Molecular Sciences Research Hub, 4615 Imperial College London, 82 Wood Lane, London, W12 0BZ, U.K.

## Abstract

A series of bis­(phosphine) cobalt carbonyl complexes were prepared and investigated *in situ* using cyclic voltammetry, ^1^H, ^31^P, and ^59^Co NMR spectroscopy (including DOSY), and IR spectroscopy. X-ray diffraction analysis on isolated single crystals confirmed that complex speciation was highly dependent on the nature of the bis­(phosphine) and ligand-to-metal stoichiometry. Reactions of [Co_2_(CO)_8_] with 1,2-bis­(dicyclohexylphosphino)­ethane (DCyPE) and 1,2-bis­(diphenylphosphino)­benzene (DPPBz) led to disproportionation of the precursor, affording cationic *κ* ^2^-Co­(I) bis­(phosphine) complexes with Co­(−I) tetracarbonyl cobaltate counteranions. In contrast, ferrocenyl bis­(phosphine) ligands 1,1′-bis­(diphenylphosphino)­ferrocene (DPPF), 1,1′-bis­(di-isopropylphosphino)­ferrocene (D^i^PrPF), and 1,1′-bis­(dicyclohexylphosphino)­ferrocene (DCyPF), displayed different reactivity. D^i^PrPF and DCyPF appeared to form polymeric species at 1:1 ligand-to-metal ratios but favored the formation of discrete neutral dinuclear complexes of the type [(μ^2^-L)­Co_2_(CO)_6_] when 0.5 equiv of ligand was employed. Reaction of [Co_2_(CO)_8_] with DPPF yielded a significantly larger molecular assembly. Single-crystal X-ray diffraction revealed an unprecedented metallocyclic structure comprising six cobalt tricarbonyl centers interconnected by four bridging DPPF ligands.

## Graphical abstract

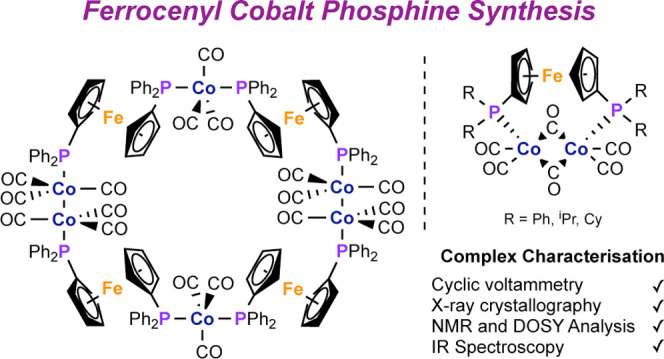

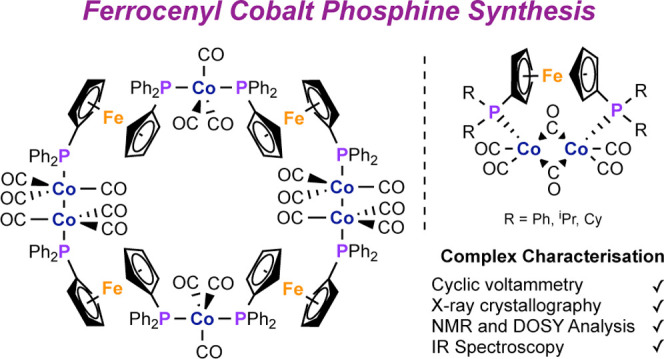

Cobalt complexes have attracted significant attention as catalysts for a variety of transformations, including hydroformylation, carbonylation, and hydrogenation, due to their high reactivity and relatively low cost.^1–7^ Recently, visible-light-driven approaches have been the focus for these catalytic reactions.^7–9^ As part of these approaches, there is continued interest in discovery of ‘next-generation’ cobalt catalysts supported by phosphine ligands.^4,10–12^

The formation of cobalt bis­(phosphine) complexes from reaction of phosphine ligands such as DPPF, DCyPE, and DPPBz with Co­(−I), Co­(I), and Co­(II) sources is relatively well understood.^3,4,12–20^ By comparison, investigation of [Co_2_(CO)_8_] as a synthetic precursor for cobalt phosphine complexes is less explored despite the commercial accessibility of this reagent.^21–25^ Herein we report a series of reactions of [Co_2_(CO)_8_] with chelating phosphines.

A range of bis­(phosphine) ligands were combined with [Co_2_(CO)_8_] in DMF, and the reaction mixtures analyzed by cyclic voltammetry (CV). Initial findings separated the ligands into two distinct classes, one class containing DCyPE and DPPBz and the other DPPF and its derivatives. DCyPE and DPPBz appear to form Co­(I) and Co­(−I) centers from disproportionation of [Co_2_(CO)_8_]. The presence of the cobaltate anion [Co­(CO)_4_]^−^ was confirmed via ^59^Co NMR spectroscopy (δ_Co_ ≈ −3000 ppm), and CV traces reflected two distinct reduction events consistent with reduction of Co­(I) to Co(0) and onward to Co­(−I) (Figure 1).

**Figure 1:**
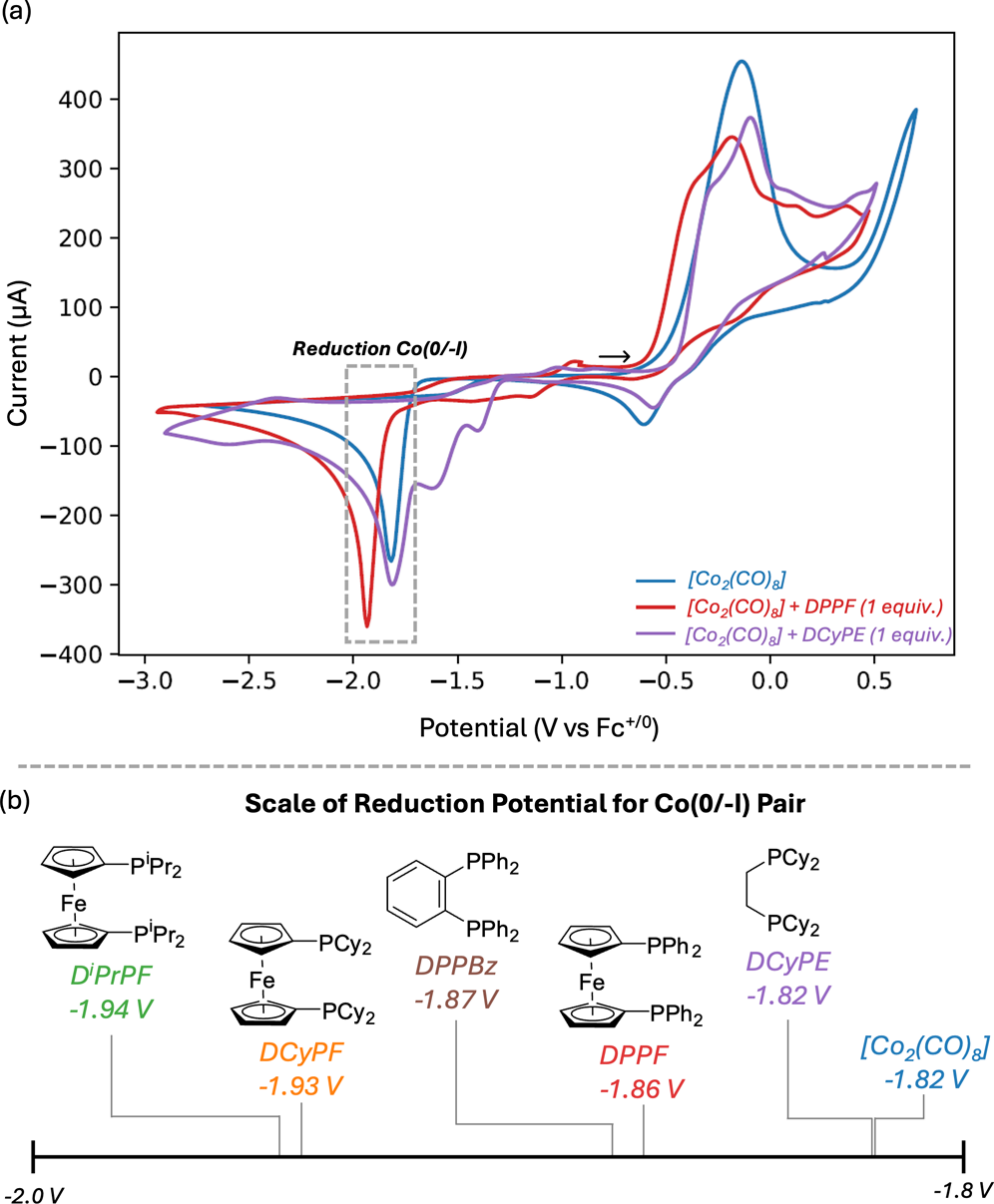
(a) Cyclic voltammetry comparison of [Co_2_(CO)_8_] and *in situ* formed complexes with DCyPE and DPPF (10 mM). (b) Analysis of the reduction potential for the Co(0)/Co­(−I) couple measured for solution mixtures of [Co_2_(CO)_8_] with bis­(phosphine) ligands (1.0 equiv.).

By comparison, limited evidence of Co­(−I) or Co­(I) formation was present for the ferrocenyl bis­(phosphine) class of ligands (DPPF, D^i^PrPF, and DCyPF). Instead, CV traces aligned closely with starting material [Co_2_(CO)_8_], albeit at more negative potentials, which suggested that complexation of these ligands might occur without disproportionation.

Analysis of redox potentials for these *in situ* generated cobalt complexes may be informative for application in catalysis.^25^ From the CV analysis, the reduction potential from Co(0) to Co­(−I) with varied ligands is highlighted (Figure 1b). Within the field of hydroformylation, catalyst speciation is frequently debated. Some researchers suggest that the anion is a key catalytic species, while others report that Co­(I) and Co­(II) species are important on-the-cycle intermediates.^13^

To gain further insight into the structures of *in* *situ* generated complexes, further reactions and spectroscopic analysis were conducted. The 1:1 reaction of DCyPE and [Co_2_(CO)_8_] proceeded spontaneously at room temperature and formed a single resonance in the ^31^P NMR spectrum (CDCl_3_) at δ 110.2 ppm. In combination with a sharp ^59^Co NMR resonance consistent with [Co­(CO)_4_]^−^ (δ_Co_ = −2995.3 ppm), the product could be assigned as [(κ^2^-DCyPE)­Co­(CO)_3_]^+^­[Co­(CO)_4_]^−^ (**1a**) (Figure 2). Reactions using two equivalents of ligand gave rise to new ^31^P NMR resonances around 67.5 ppm while retaining the ^59^Co NMR resonance, suggesting further substitution at the Co­(I) center to form complex [(κ^2^-DCyPE)_2_Co­(CO)]^+^­[Co­(CO)_4_]^−^ (**1b**). Reaction of [Co_2_(CO)_8_] with DPPBz showed comparable shifts by ^31^P NMR and ^59^Co NMR spectroscopy. IR spectroscopic analysis of the formed DPPBz complex closely agreed with previously synthesized [(κ^2^-DPPBz)­Co­(CO)_3_]^+^[BF_4_]^−^, suggesting [(κ^2^-DPPBz)­Co­(CO)_3_]^+^­[Co­(CO)_4_]^−^ (**2a**) had been formed.^12^ The speciation of generated complexes was consistent in DMF and THF/2-MeTHF.

**Figure 2:**
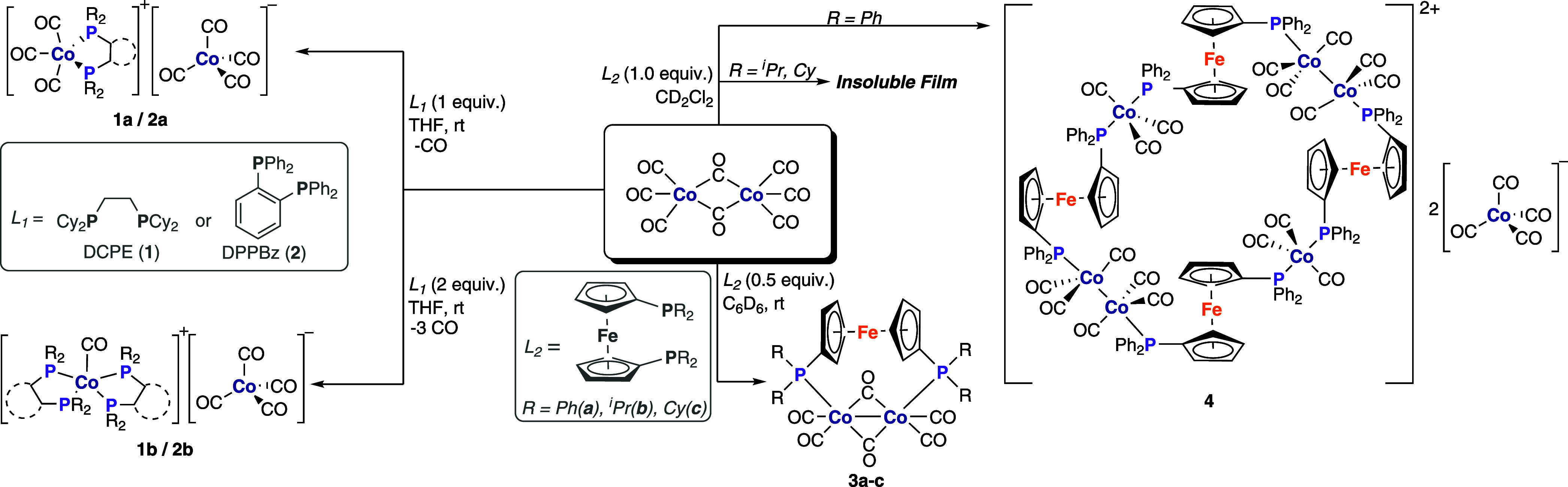
Diverse reactivity of [Co_2_(CO)_8_] with different bis­(phosphine) ligands.

By comparison, ferrocene-containing ligands (DPPF, D^i^PrPF, and DCyPF) showed different reactivity. At both 1:1 and 2:1 ratios of bis­(phosphine):[Co_2_(CO)_8_] and across multiple solvents, formation of a solid film from solution was observed. This insoluble material likely occurs due to the ability of these bis­(phosphine) ligands to bridge metal centers, creating a coordination polymer. These reactions were then investigated at 1:2 ratios of bis­(phosphine):[Co_2_(CO)_8_]. At this stoichiometry, homogeneous samples were obtained. New resonances were observed by ^31^P NMR spectroscopy (C_6_D_6_) at δ_P_ = 40.6 (DPPF), 44.3 (D^i^PrPF), and 36.5 ppm (DCyPF). These resonances were also observed in THF in lower yields, suggesting an unstable intermediate may be forming.

Analysis of the reaction mixtures by ^59^Co NMR spectroscopy confirmed the absence of [Co­(CO)_4_]^−^. Line broadening in the ^31^P NMR spectra suggested coupling between the NMR-active ^31^P and ^59^Co nuclei. In combination, these data indicate that disproportionation of [Co_2_(CO)_8_] does not occur, which is in agreement with the CV analysis.

To characterize reaction products further, we grew X-ray-quality single crystals. The product of the reaction between DCyPE and [Co_2_(CO)_8_] was confirmed as disproportionation product **1a** by X-ray diffraction (Figure 3). Solid-state analysis of the related hydride complex [(DCyPE)­Co­(CO)_2_H] has been previously reported by Chirik and co-workers.^8^

**Figure 3:**
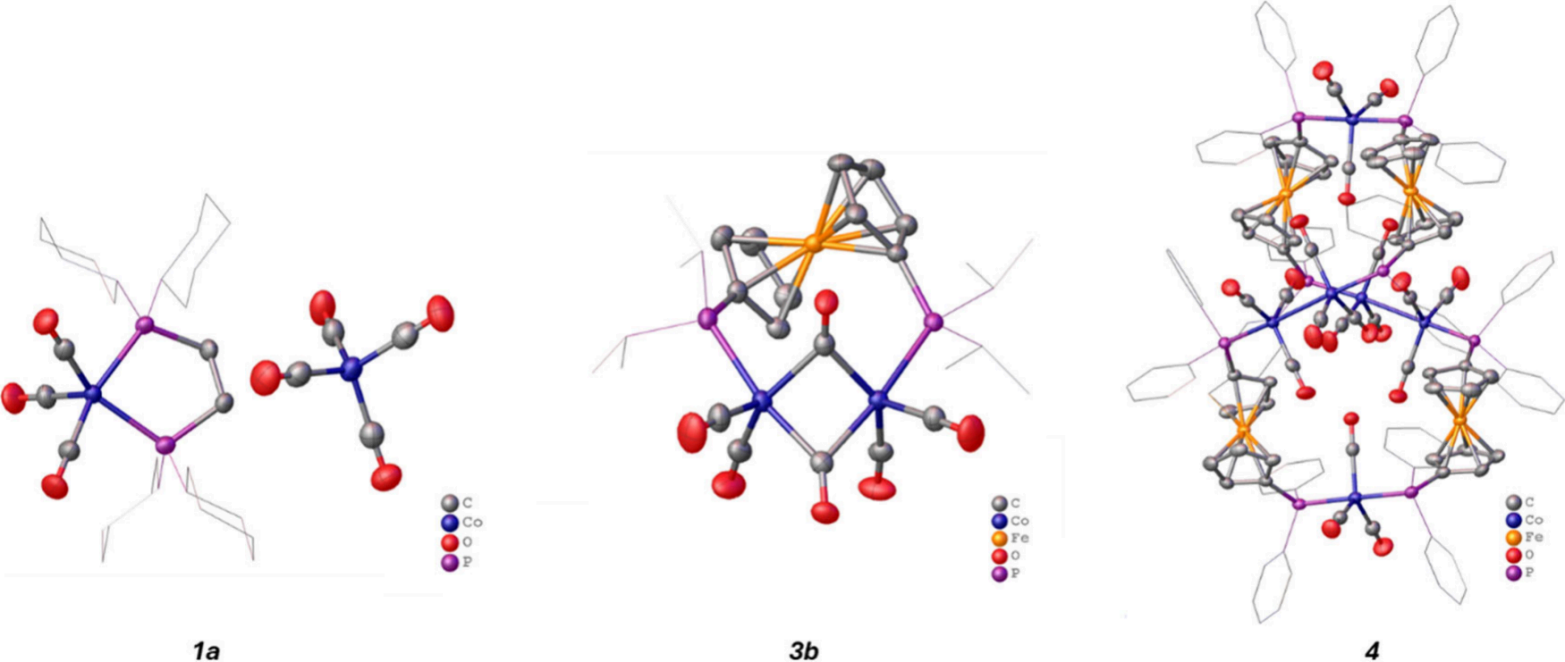
X-ray diffraction structures of **1a**, **3b**, and **4**. Hydrogen atoms are omitted for clarity in all structures. Cobaltate counterions and included solvent are omitted in **4**.

The cobalt–phosphine bond lengths of **1a** were consistent, taking values of 2.2501(13) Å and 2.2455(12) Å. Co­(I)–C bond lengths were consistent around 1.78 Å except for a single Co­(I)–C bond, which was elongated to 1.831(5) Å. This elongation likely arises due to a carbonyl ligand occupying the axial site of a square-based pyramidal geometry (τ_5_ = 0.1).

Separately, a single crystal was grown from the reaction of D^i^PrPF with [Co_2_(CO)_8_] in Me-THF and subsequently analyzed by X-ray diffraction (Figure 3, 3b). The crystal structure **3b** had the formula [(μ_2_ **-**D^i^PrPF)­Co_2_(CO)_6_], with the bis­(phosphine) bridging between two cobalt centers. **3b** forms via ligand exchange with 2.0 equiv. of CO at [Co_2_(CO)_8_].

The structure is reminiscent of the major isomer [Co_2_(CO)_8_] adopted in the solution state (*C*_2*v*_ symmetry). The Co–Co distance of **3b** (2.5388(4) Å) closely aligns with that of CO-bridged [Co_2_(CO)_8_] (2.55361(4)–2.5392(4) Å), which would suggest that there is no direct Co–Co bonding despite close proximity of the metal centers.^26^ Retained bridging Co–C bonds were asymmetrical, with each cobalt coordinating via a shorter (1.916(2) to 1.939(2) Å) and relatively longer bond (1.962(2) to 1.966(2) Å). Terminal Co–C bonds were consistent in length between 1.770(2) and 1.801(2) Å. Co–P bond lengths were within standard deviation at 2.3045(5) and 2.3051(5) Å, respectively, suggesting minimal conformational strain despite a twisted, chiral structure.

Further study via ^31^P and DOSY NMR spectroscopy confirmed that species corresponding to **3b** exist in solution for each DPPF (**3a**), D^i^PrPF (**3b**), and DCyPF (**3c**) ligand analogue (Figures S34–S43).

Finally, treatment of [Co_2_(CO)_8_] with DPPF in CD_2_Cl_2_ gave rise to a red crystalline solid, compound **4**, following heating to 50 °C for 1 h. Analysis by X-ray diffraction gave the chemical formula [Co_6_Fe_4_P_8_C_154_H_112_O_18_]^2+^­[Co­(CO)_4_]^−^_2_ (Figure 3). The structure corresponds to a multimetallic macrocycle containing three unique cobalt environments at formal oxidation states of −I, 0, and I as well as four ferrocene units with inequivalent phosphine environments.

In all environments, ferrocenyl bis­(phosphines) were bound in monodentate motifs. Co­(I)–P bond lengths were slightly elongated versus Co(0)–P bonds (2.2299(5) vs 2.1886(5) Å). These values correlated closely with Co(0)–P bond lengths found in crystalline [Co_2_(CO)_7_(PPh_3_)] (2.1976(10) Å).^27^ The Co–Co bond lengths were 2.6798(3) Å, closely aligned with the metal–metal bonded isomer of [Co_2_(CO)_8_] (2.700(2) Å) (*D*_3*d*_ symmetry) and were notably longer than those observed in **3b**.^28^ DOSY NMR spectroscopic analysis suggested that this isolated species is present in solution at 1:1 mixtures of ligand to [Co_2_(CO)_8_] (Figures S34–S37). Conversely, D^i^PrPF and DCyPF ligands gave no evidence for cluster formation by DOSY analysis, suggesting that the phenyl substitution in DPPF is crucial for mediating macrocycle formation.

In summary, we report the synthesis and characterization of a series of new cobalt carbonyl complexes bearing bis­(phosphine) ligands. CV was employed as a probe for speciation, while NMR and IR spectroscopic analyses further corroborated speciation. Collectively, these data revealed three distinct classes of complexes that arise from structural differences in the bis­(phosphine) ligands. In systems containing non-ferrocenyl ligands, namely, DCyPE and DPPBz, disproportionation of [Co_2_(CO)_8_] was observed, leading to the formation of ionic cobalt species. In contrast, ferrocenyl bis­(phosphine) ligands gave rise to more diverse architectures, including neutral dicobalt complexes, and, notably for DPPF, a unique hexacobalt macrocyclic assembly. Ongoing studies are directed toward elucidating the reactivity and functional potential of these cobalt–phosphine systems for future applications.

## Supplementary Material



## Notes

Detailed experimental procedures, X-ray crystallography, NMR and IR spectroscopy, cyclic voltammetry (PDF)

## ABBREVIATIONS

DPPF: 1,1′-Bis­(diphenylphosphino)­ferrocene
D^i^PrPF: 1,1′-Bis­(di-*iso*propylphosphino)­ferrocene
DCyPF: 1,1′-Bis­(dicyclohexylphosphino)­ferrocene
DCyPE: 1,2-Bis­(dicyclohexylphosphino)­ethane
DPPBz: 1,2-Bis­(diphenylphosphino)­benzene
Fc: Ferrocene, bis­(η^5^-cyclopentadienyl)­iron
CV: Cyclic Voltammetry
