# Supplementary material for: Flash Communication: Reactivity of Bis(phosphine) Ligands with Dicobalt Octacarbonyl
Source: Organometallics. 2026 Jul 17;45(15):1759–63. doi: 10.1021/acs.organomet.6c00176 (PMC13463686; doi:10.1021/acs.organomet.6c00176)
Supplement: Supplementary file 1 [file om6c00176_si_001.pdf]

Supporting Information for

Flash Communication: Reactivity of *Bis*(phosphine) Ligands with Dicobalt Octacarbonyl

Rowan M. Bailey, Tsz-Kan Ma, Dominykas Dautoras, Benedek Stadler, Álvaro García-Romero, Andrew J. P. White, James A. Bull, Mark R. Crimmin, and Philip W. Miller**

Department of Chemistry, Molecular Sciences Research Hub, Imperial College London, 82 Wood Lane, Shepards Bush, London, W12 0BZ, UK

* corresponding author email:

Mark R. Crimmin: m.crimmin@imperial.ac.uk

Philip W. Miller: philip.miller@imperial.ac.uk

Contents

General Experimental 3

Synthetic Procedures 3

General Procedure for Cobalt Complex Synthesis: 3

In situ NMR and FT-IR spectroscopic characterisation 4

Synthesis and Isolation of Cobalt Complexes 5

Synthesis of **1a** - $[(DCPE)Co(CO)_4]^+[Co(CO)_4]^-$ 5

Synthesis of **3b** - $[(\mu^2\text{-}D^iPrPF)Co_2(CO)_6]$ 5

Synthesis of **4** - $[(DPPF)_4Co_6(CO)_{18}]^{2+}[Co(CO)_4]^-_2$ 6

Cyclic Voltammetry 6

General Procedure for Cyclic Voltammetry Analysis: 6

Cyclic Voltammogram Data 7

In-Situ NMR Spectra 14

Complexes using the DCPE Ligand 14

Complexes using the DPPBz Ligand 15

Complexes using the DPPF ligand 15

Complexes using the D^{i}PrPF Ligand 16

Complexes using the DCyPF Ligand 16

FT-IR Analysis 17

Complexes using the DCPE Ligand 17

Complexes using the DPPBz Ligand 18

Complexes using the DPPF Ligand 19

Complexes using the D^{i}PrPF Ligand 20

Complexes using the DCyPF Ligand 21

NMR Spectra for Isolated Compounds 22

DOSY Experiments 26

Single Crystal X-ray Diffraction Data 31

The X-ray crystal structure of **1a** 32

The X-ray crystal structure of **3b** 32

The X-ray crystal structure of **4** 33

References 34

General Experimental

Unless otherwise specified, reactions were carried out using standard Schlenk line and glovebox techniques under an N_2 atmosphere. Glassware was dried for a minimum of 12 h at 100 °C prior to use. Chemicals were purchased from commercial sources (Merck, Alfa Aesar, VWR, Fischer, Fluorochem) and analysed by NMR spectroscopy and Gas Chromatography (GC) to ensure purity was sufficient. All solvents used in the study were degassed *via* three freeze-pump-thaw cycles and dried over 3 Å molecular sieves, then taken into a MBRAUN Labmaster glovebox. Solid samples were dried using a high vacuum line prior to cycling into the same glovebox. [$Co_2(CO)_8$] was stored in a -35 °C freezer in the glovebox due to its light and temperature sensitivity. This compound was only handled in a glovebox or using Schlenk techniques. A portable CO detector was used as an additional safety precaution.

^{1}H, ^{31}P and ^{59}Co NMR spectra were obtained using a Bruker 400 MHz spectrometer. Chemical shifts (δ) were referenced relative to NMR solvent residual peaks and data was analysed using a MestReNova software package. FT-IR measurements were taken on an Agilent Cary630 spectrometer placed within an MBraun glovebox.

Cyclic voltammograms were recorded at room temperature using a Metrohm Autolab PGSTAT204 workstation. Data analysis was performed with Nova 2.0 software. A three-electrode undivided electrochemical cell was employed, comprising a glassy carbon disc electrode (2 mm diameter) as the working electrode, a platinum wire as the counter electrode, and a silver wire as the pseudo-reference electrode.

Before each measurement, the glassy carbon electrode was polished on a polishing pad with a water-alumina slurry using figure-eight motions, then rinsed with deionized water, then acetone. The solution of interest was sparged with argon for 2 minutes before data collection. Unless otherwise stated, the scan direction was initiated in the positive direction from 0 V. Ferrocene was used as an internal standard after each measurement, and all potentials were referenced to the ferrocenium/ferrocene redox couple. The current is reported in μA, and applied voltages were corrected using positive feedback iR compensation.

CAUTION: Dicobalt octacarbonyl [$Co_2(CO)_8$] is a highly toxic, air-sensitive organometallic compound that can release carbon monoxide upon decomposition. It is harmful if inhaled, ingested, or absorbed through the skin, and may cause irritation to the respiratory system and skin. The material should be handled strictly under inert atmosphere using appropriate containment (e.g. glovebox or Schlenk techniques).

Synthetic Procedures

General Procedure for Cobalt Complex Synthesis:

In a N_2 filled glovebox, [$Co_2(CO)_8$] (240 mg, 0.7 mmol) was weighed into a vial and dissolved in THF or 2-MeTHF (14 mL). *Bis*(phosphine) ligand (0.1 mmol, 1 equiv.) was weighed into a separate vial and dissolved in THF or 2-MeTHF (2 mL). A portion of the cobalt stock solution (2 mL) was added into the phosphine stock solution and subsequently stirred. Following 10 minutes of stirring, samples were transferred into a J. Young NMR tube containing a C_6D_6 capillary insert and analysed by ^{31}P and ^{59}Co NMR spectroscopy. Where analysis suggested incomplete reaction, samples were heated in an oil bath to 60 °C for 1 h and reanalysed by the same NMR spectroscopic methods. Upon reaction completion, samples were cycled back into a glovebox and analysed by FT-IR spectroscopy (ATR, thin film).

The samples were then filtered into small vials using PTFE syringe filters (0.45 μm pore size, 13 mm) and stored in the glovebox freezer to promote crystallisation. As appropriate, addition of n-pentane was used to further promote crystal growth.

In situ NMR and FT-IR spectroscopic characterisation

1a - $[(DCPE)Co(CO)_4]^+[Co(CO)_4]^-$: ^{31}P NMR (162 MHz, THF-H_8) δ 107.6. **^{59}Co NMR (95 MHz, THF-H_8)** δ -3004. **FTIR (v_{max}, cm^{-1})** 2070, 2002, 1960, 1924, 1880, 1737.

1b - $[(DCPE)_2Co(CO)]^+[Co(CO)_4]^-$: ^{31}P NMR (162 MHz, THF-H_8) δ 68.0, 64.5. **^{59}Co NMR (95 MHz, THF-H_8)** δ -3005. **FTIR (v_{max}, cm^{-1})** 1923, 1893.

2a - $[(DPPBz)Co(CO)_3]^+[Co(CO)_4]^-$: ^{1}H NMR (400 MHz, THF-H_8) δ 7.64 – 7.51 (m, 8H), 7.50 – 7.28 (m, 12H), 7.22 (m, 1H), 7.12 (m, 3H). **^{31}P NMR (162 MHz, THF-H_8)** δ 63.8. **^{59}Co NMR (95 MHz, THF-H_8)** δ -3008. **FTIR (v_{max}, cm^{-1})** 2051, 1982, 1928, 1883, 1786, 1732.

2b - $[(DPPBz)_2Co(CO)]^+[Co(CO)_4]^-$: ^{1}H NMR (400 MHz, THF-H_8) δ 7.53 (d, J = 8.1 Hz, 4H), 7.43 (d, J = 7.2 Hz, 2H), 7.39 (d, J = 7.1 Hz, 3H), 7.31 (s, 8H), 7.26 – 7.12 (m, 19H), 7.08 (t, J = 7.5 Hz, 6H), 6.87 (t, J = 7.6 Hz, 2H), 6.69 (d, J = 10.8 Hz, 4H). **^{31}P NMR (162 MHz, THF-H_8)** δ 60.6. **^{59}Co NMR (95 MHz, THF-H_8)** δ -3008. **FTIR (v_{max}, cm^{-1})** 1927, 1882, 1732.

3a – $[(\mu^2$-DPPF$)Co_2(CO)_6]$: ^{1}H NMR (500 MHz, C_6D_6) δ 7.77 (m, 2H), 7.63 (m, 3H), 7.42 (m, 3H), 7.10 – 7.02 (m, 3H), 7.01 – 6.96 (m, 4H), 6.92 (m, 5H), 4.64 (s, 1H), 4.53 – 4.47 (m, 1H), 4.20 (dd, J = 2.0, 2.0 Hz, 2H), 3.90 – 3.80 (m, 4H).**^{31}P NMR (202 MHz, C_6D_6)** δ 40.6. **^{1}H DOSY NMR:** 8.7 x10^{-10} $m^2 s^{-1}$. **FTIR (v_{max}, cm^{-1})** 2075, 2019, 1972, 1948, 1786.

3b - $[(\mu^2$-D^{i}PrPF$)Co_2(CO)_6]$: ^{1}H NMR (500 MHz, C_6D_6) δ 4.37 (s, 4H), 3.96 (t, J = 2.0 Hz, 4H), 2.10 (m, 4H), 1.11 – 1.00 (m, 12H), 0.99 - 0.86 (m, 12H). **^{31}P NMR (202 MHz, C_6D_6)** δ 44.3. **FTIR (v_{max}, cm^{-1})** 2052, 2026, 1982, 1962, 1942, 1859, 1781.

3c - $[(\mu^2$-DCyPF$)Co_2(CO)_6]$: ^{1}H NMR (500 MHz, C_6D_6) δ 4.50 (s, 4H), 4.02 (s, 4H), 2.07 – 1.99 (m, 7H), 1.93 – 1.84 (m, 4H), 1.72 – 1.67 (m, 4H), 1.63 – 1.58 (m, 4H), 1.53 – 1.48 (m, 4H), 1.46 – 1.41 (m, 4H), 1.32 – 1.22 (m, 5H), 1.17 – 1.02 (m, 12H). **^{31}P NMR (202 MHz, C_6D_6)** δ 36.5. **^{1}H DOSY NMR:** 8.5 x10^{-10} $m^2 s^{-1}$. **FTIR (v_{max}, cm^{-1})** 2053, 2024, 1982, 1961, 1855, 1782.

4 - $[(DPPF)_4Co_6(CO)_{18}]^{2+}[Co(CO)_4{}^-{}_2]$: ^{1}H NMR (500 MHz, C_6D_6) δ 7.58 (m, 8H), 6.95 – 6.85 (m, 72H), 4.35 (s, 20H), 4.10 (s, 12H). **^{31}P NMR (202 MHz, C_6D_6)** δ 61.71, 61.07. **^{59}Co NMR (95 MHz, THF-H_8)** δ -3007. **FTIR (v_{max}, cm^{-1})** 2035, 1973, 1945, 1785.

Synthesis and Isolation of Cobalt Complexes

Synthesis of 1a - $[(DCPE)Co(CO)_4]^+[Co(CO)_4]^-$

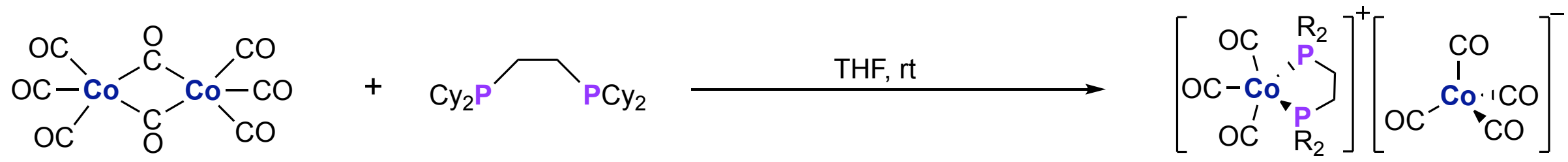

In an N_2 filled glovebox 1,2-*bis*(dicyclohexylphosphino)ethane (DCPE) (10.6 mg, 0.025 mmol) and $[Co_2(CO)_8]$ (8.5 mg, 0.025 mmol) were weighed into a vial and $CDCl_3$ (0.5 mL) was added. The mixture was transferred into a J. Young NMR tube and analysed by ^{1}H, ^{31}P and ^{59}Co NMR spectroscopy where a major product was observed at 110.2 ppm (^{31}P NMR). Single crystals suitable for X-ray diffraction were obtained by storing the solution at room temperature overnight. **Yield:** (17.4 mg, 0.023 mmol, 91%) **^{1}H NMR (400 MHz, $CDCl_3$)** δ 2.26 (m, 4H), 2.12 (br s, 4H), 1.95 (br s, 8H), 1.92 – 1.77 (m, 12H), 1.36 (m, 20H). **$^{31}P\{H\}$ NMR (162 MHz, $CDCl_3$):** δ 110.2 ppm. **^{59}Co NMR (94.9 MHz, CD_2Cl_2):** δ -2995.34 (s).

Synthesis of 3b - $[(\mu^2\text{-D}^i\text{PrPF})Co_2(CO)_6]$

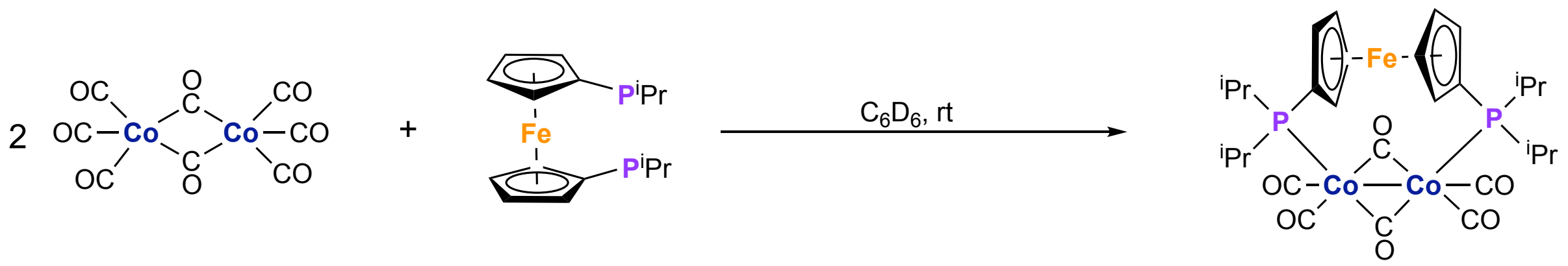

In an N_2 filled glovebox 1,1'-*bis*(di*iso*propylphosphino)ferrocene (D^{i}PrPF) (42 mg, 0.1 mmol, 0.5 equiv.) and $[Co_2(CO)_8]$ (68 mg, 0.2 mmol) were weighed into a vial and THF (6 mL) was added. An aliquot (0.5 mL) of the mixture was transferred into a J. Young NMR tube with a C_6D_6 capillary and analysed by ^{31}P and ^{59}Co NMR spectroscopy where a new major product was observed at 44.3 ppm (^{31}P NMR). The sample was filtered into a fresh vial and stored at -35 °C under anaerobic conditions. After 1 month in the freezer, orange cubic single crystals suitable for X-ray diffraction had grown. **Yield:** (51.2 mg, 0.073 mmol, 73 %) (95% purity by ^{1}H NMR analysis). **^{1}H NMR (500 MHz, C_6D_6)** δ 4.37 (s, 4H), 3.96 (t, J = 1.9 Hz, 4H), 2.10 (dt, J = 7.1, 3.4 Hz, 4H), 1.11 – 1.00 (m, 12H), 0.95 (dq, J = 14.4, 7.1 Hz, 12H). **^{31}P NMR (162 MHz, C_6D_6):** δ 44.32 (br s). **^{59}Co NMR (94.9 MHz, C_6D_6)**: δ No resonances were detected. **^{1}H DOSY NMR:** 1.05 x10^{-9} m^2 s^{-1}

Synthesis of 4 - $[(DPPF)_4Co_6(CO)_{18}]^{2+}[Co(CO)_4]^-_2$

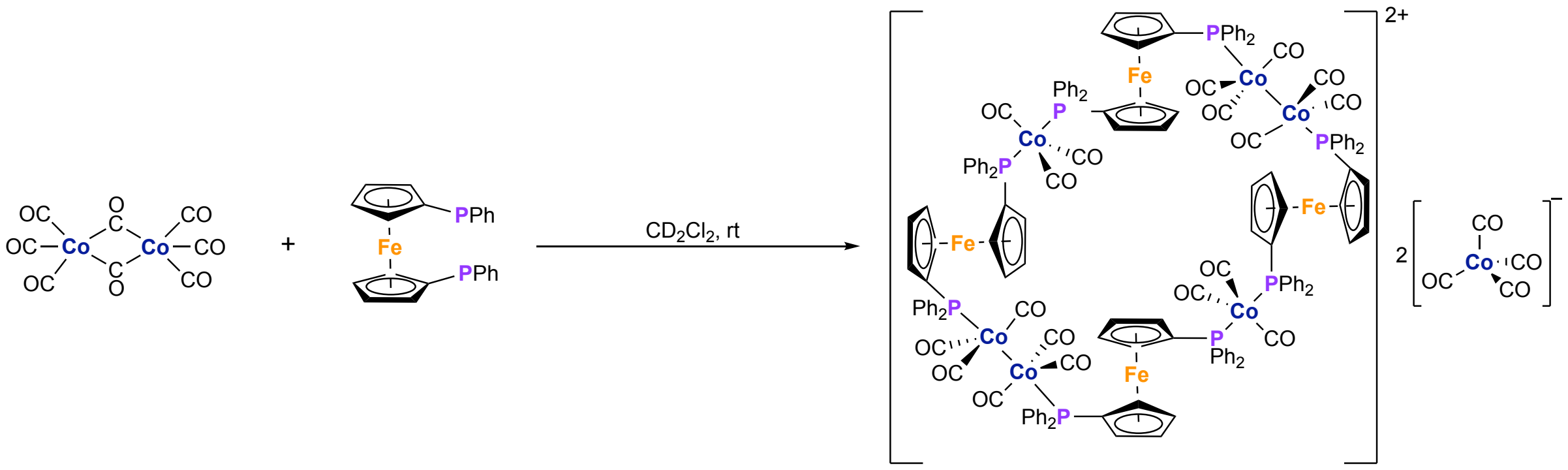

In an N_2 filled glovebox 1,1'-*bis*(diphenylphosphino)ferrocene (DPPF) (13.9 mg, 0.025 mmol) and $[Co_2(CO)_8]$ (8.5 mg, 0.025 mmol) were weighed into a vial and CD_2Cl_2 (0.5 mL) was added. The mixture was transferred into a J. Young NMR tube and analysed by ^1H, ^{31}P and ^{59}Co NMR spectroscopy where a major product was observed at 53 ppm (^{31}P NMR). Crystals suitable for X-ray diffraction were grown by storing the NMR tube at room temperature for 3 weeks. Alternatively, crystals could be grown by heating the mixture to 50 °C briefly and then storing at room temperature overnight. **Yield**: (2.5 mg, 0.00072 mmol, 11 %). **^1H NMR (400 MHz, CD_2Cl_2)** δ 8.11 (br s, 6H), 7.99 (br s, 8H), 7.74 (br s, 1H), 7.66 (br s, 3H), 7.55 (m, 16H), 7.49 – 7.33 (m, 24H), 7.32 – 7.16 (m, 16H), 6.86 (q, 6H), 4.86 (br s, 3H), 4.61 (m, 5H), 4.53 – 4.42 (m, 6H), 4.41 – 4.32 (m, 4H), 4.30 – 4.02 (m, 6H), 3.93 (s, 5H), 3.85 – 3.56 (m, 3H). **$^{31}P\{H\}$ NMR (162 MHz, CD_2Cl_2):** δ 62.5 (br s). **^{59}Co NMR (94.9 MHz, CD_2Cl_2):** δ -3004.6 (s). **^1H DOSY NMR:** 4.96 x10^{-10} m^2 s^{-1}

Cyclic Voltammetry

General Procedure for Cyclic Voltammetry Analysis:

In a N_2 filled glovebox, $[Co_2(CO)_8]$ (67.5 mg, 0.4 mmol) was weighed into a vial and dissolved in DMF (2 mL). *Bis*(phosphine) ligand (0.04 or 0.08 or 0.16 mmol, 0.5 or 1 or 2 equiv.) was weighed into a separate vial and dissolved in DMF (2 mL). A portion of the cobalt stock solution (2 mL) was subsequently added to the corresponding phosphine stock solution, and the mixture was stirred for 10 minutes. Samples were then transferred into a J. Young NMR tube containing a C_6D_6 capillary insert and heated in an oil bath to 60 °C for 1 h. The samples were cooled to room temperature and analysed by ^{31}P and ^{59}Co NMR spectroscopy. Separately, a solution of tetrabutylammonium hexafluorophosphate (775 mg, 2 mmol) in DMF (10 mL) and solution of ferrocene (7.4 mg, 0.04 mmol) in DMF (2 mL) were prepared.

Background conductivity was confirmed using 2 mL of electrolyte solution. Following this, 1 mL of the electrolyte solution was removed from the voltammetry cell and analyte solution (1 mL) was added under a flow of argon. Argon was bubbled through all solutions for 2 minutes prior to sample analysis. Once 3 scans had been measured, ferrocene solution (0.5 mL) was added to the cell for calibration. This procedure was repeated for all samples tested.

Cyclic Voltammogram Data

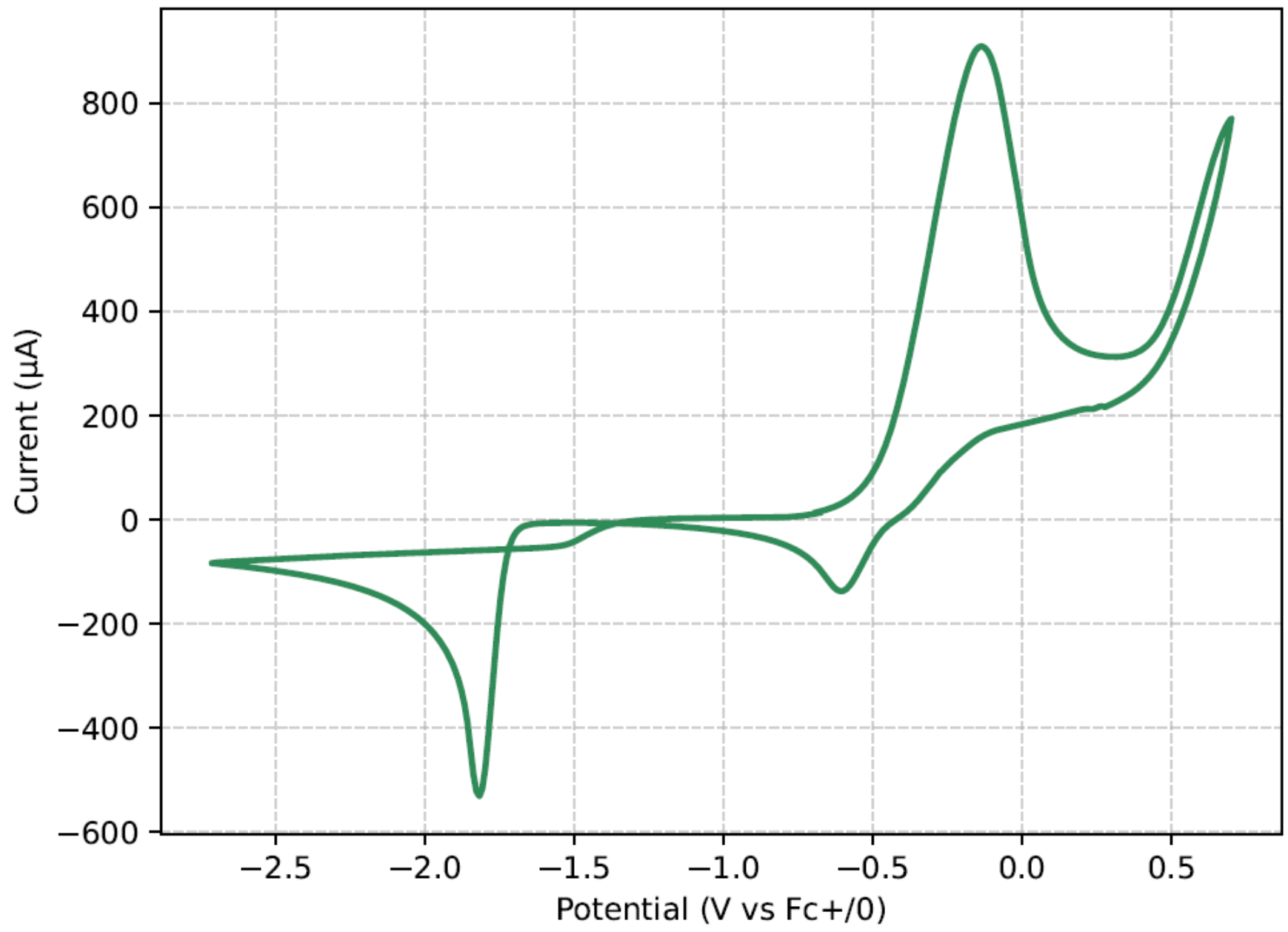

Figure S1. *Cyclic voltammogram of [$Co_2(CO)_8$] (10 mM). Conditions: NBu_4PF_6 (0.10 M in DMF). Purged with Ar. Scan rate: 100 mVs^{-1}. Scan direction: Positive direction from 0 V.*

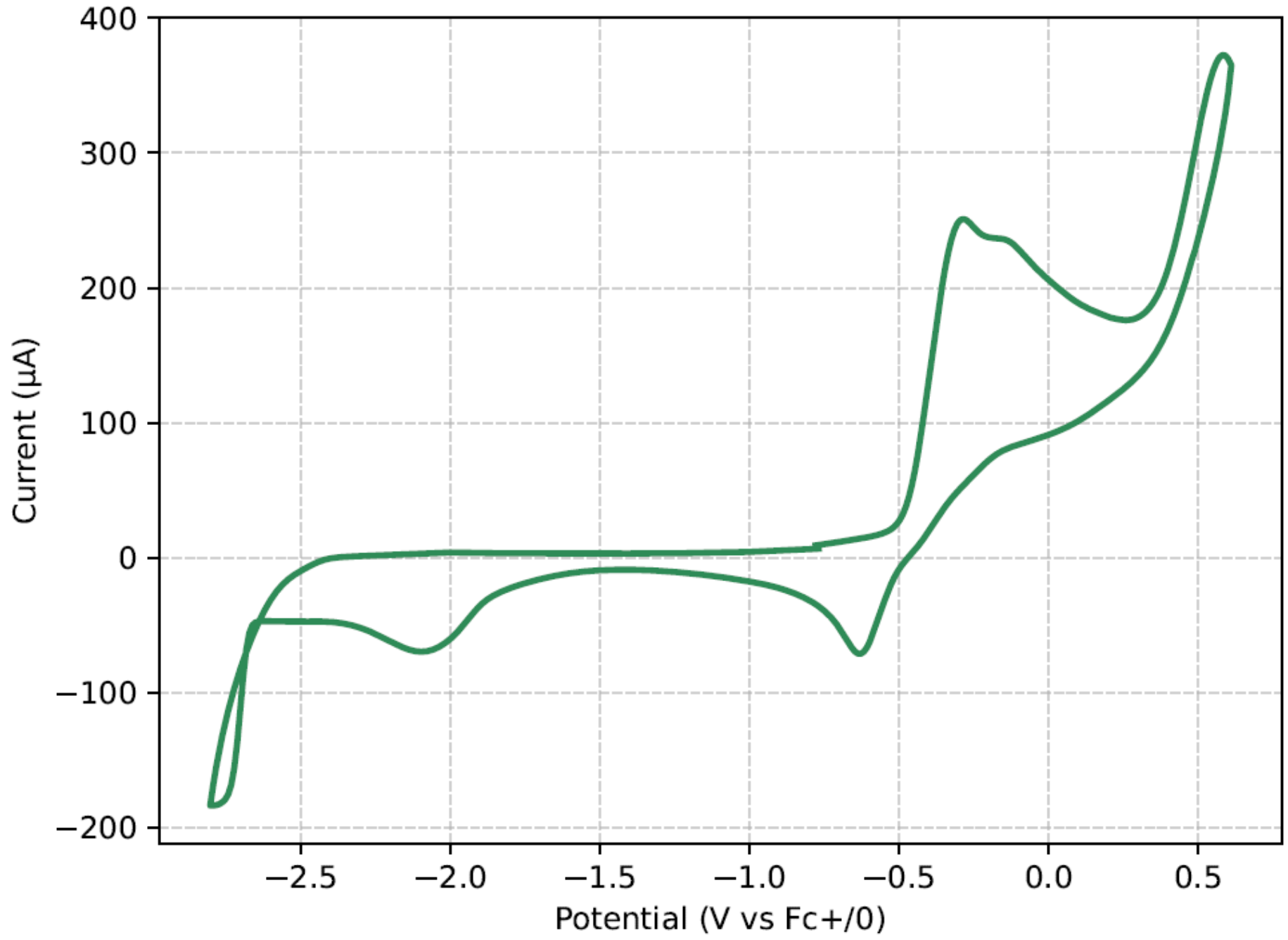

Figure S2. *Cyclic voltammogram of K[$Co(CO)_4$] (10 mM). Conditions: NBu_4PF_6 (0.10 M in DMF). Purged with Ar. Scan rate: 100 mVs^{-1}. Scan direction: Positive direction from 0 V.*

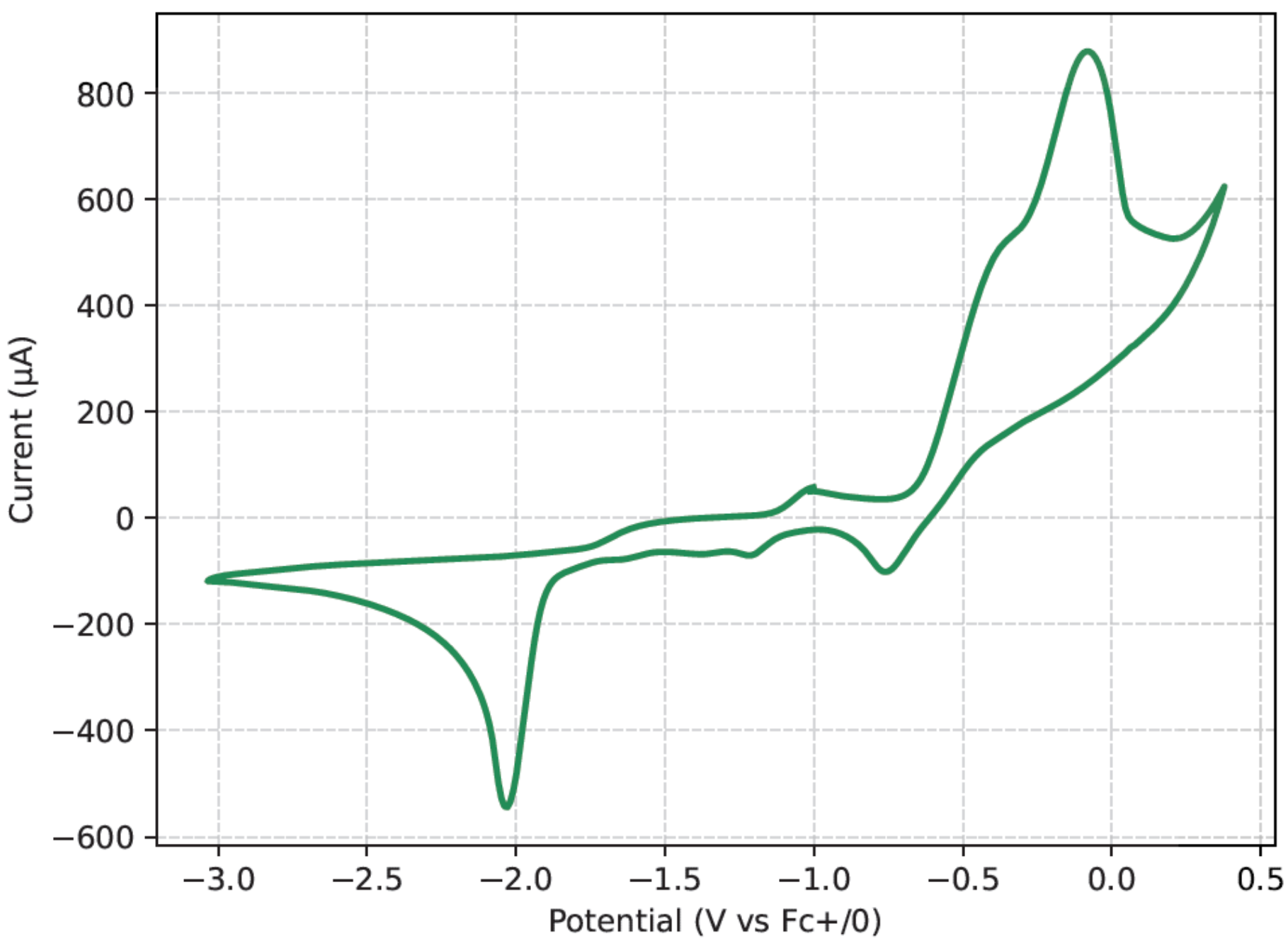

Figure S3. *Cyclic voltammogram of [$Co_2(CO)_8$] (10 mM) and DPPF (5 mM, 0.5 equiv.). Conditions: NBu_4PF_6 (0.10 M in DMF). Purged with Ar. Scan rate: 100 mVs^{-1}. Scan direction: Positive direction from 0 V.*

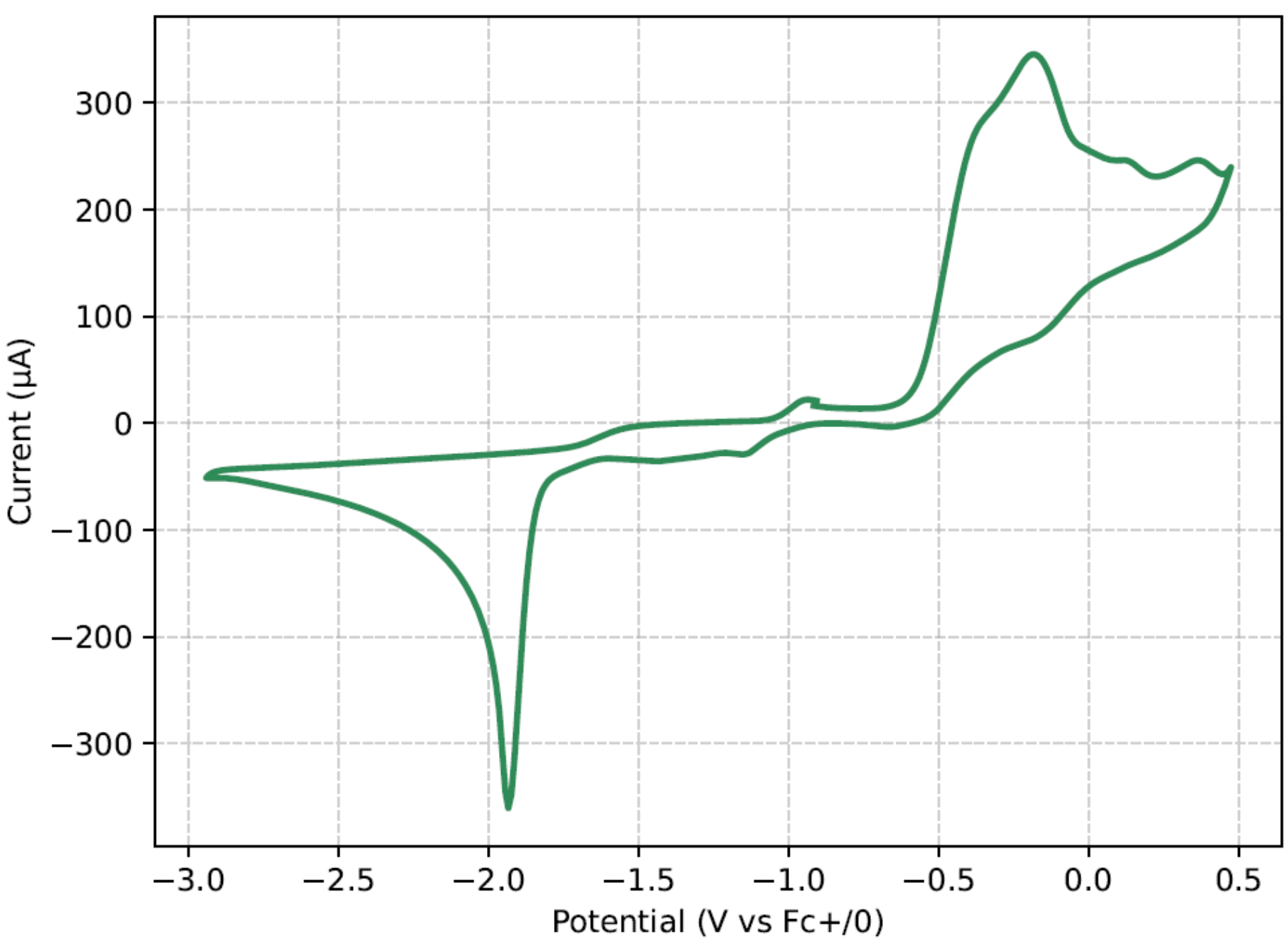

Figure S4. *Cyclic voltammogram of [$Co_2(CO)_8$] (10 mM) and DPPF (10 mM, 1 equiv.). Conditions: NBu_4PF_6 (0.10 M in DMF). Purged with Ar. Scan rate: 100 mVs^{-1}. Scan direction: Positive direction from 0 V.*

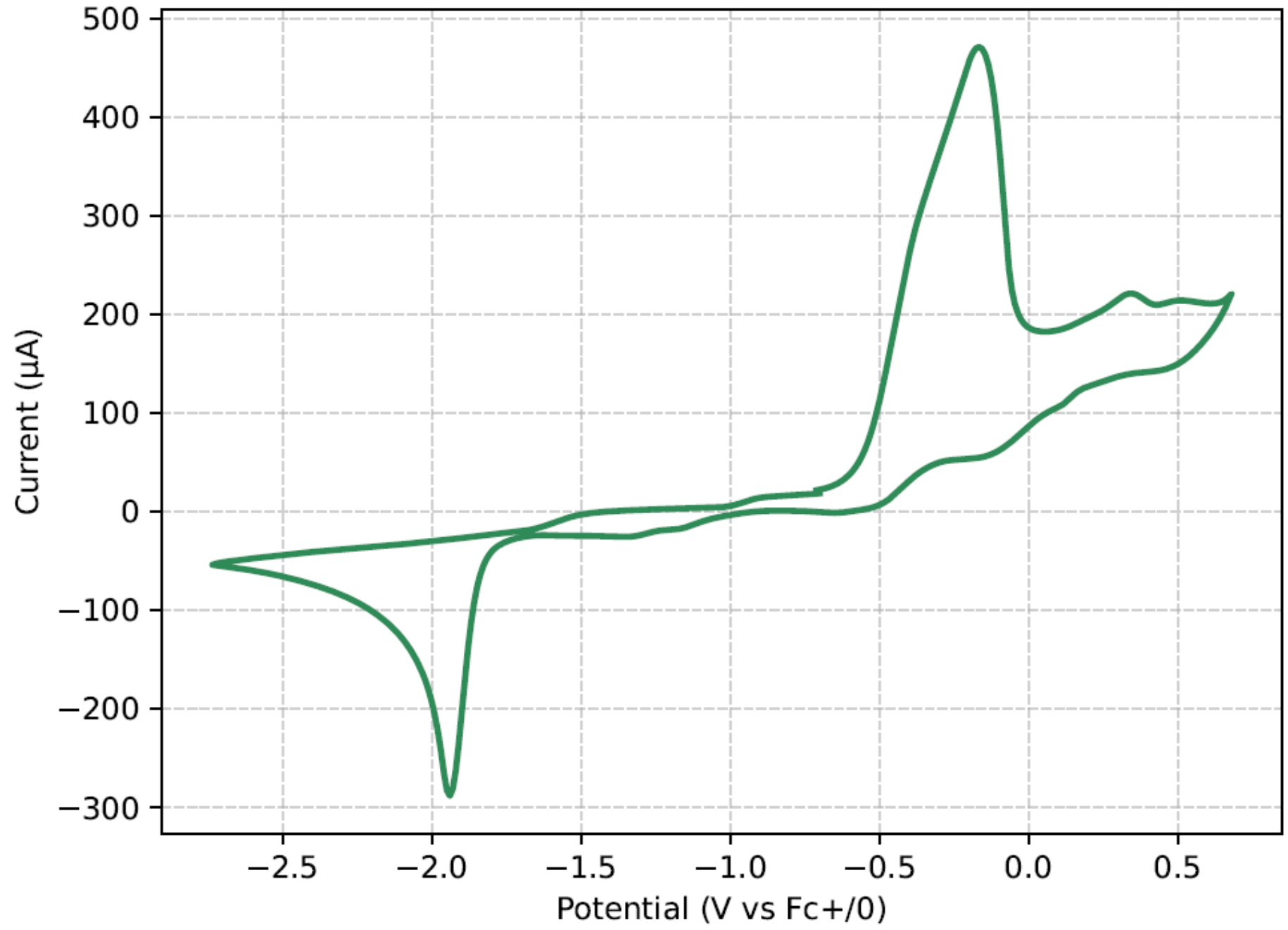

Figure S5. *Cyclic voltammogram of [$Co_2(CO)_8$] (10 mM) and DPPF (20 mM, 2 equiv.). Conditions: NBu_4PF_6 (0.10 M in DMF). Purged with Ar. Scan rate: 100 mVs^{-1}. Scan direction: Positive direction from 0 V.*

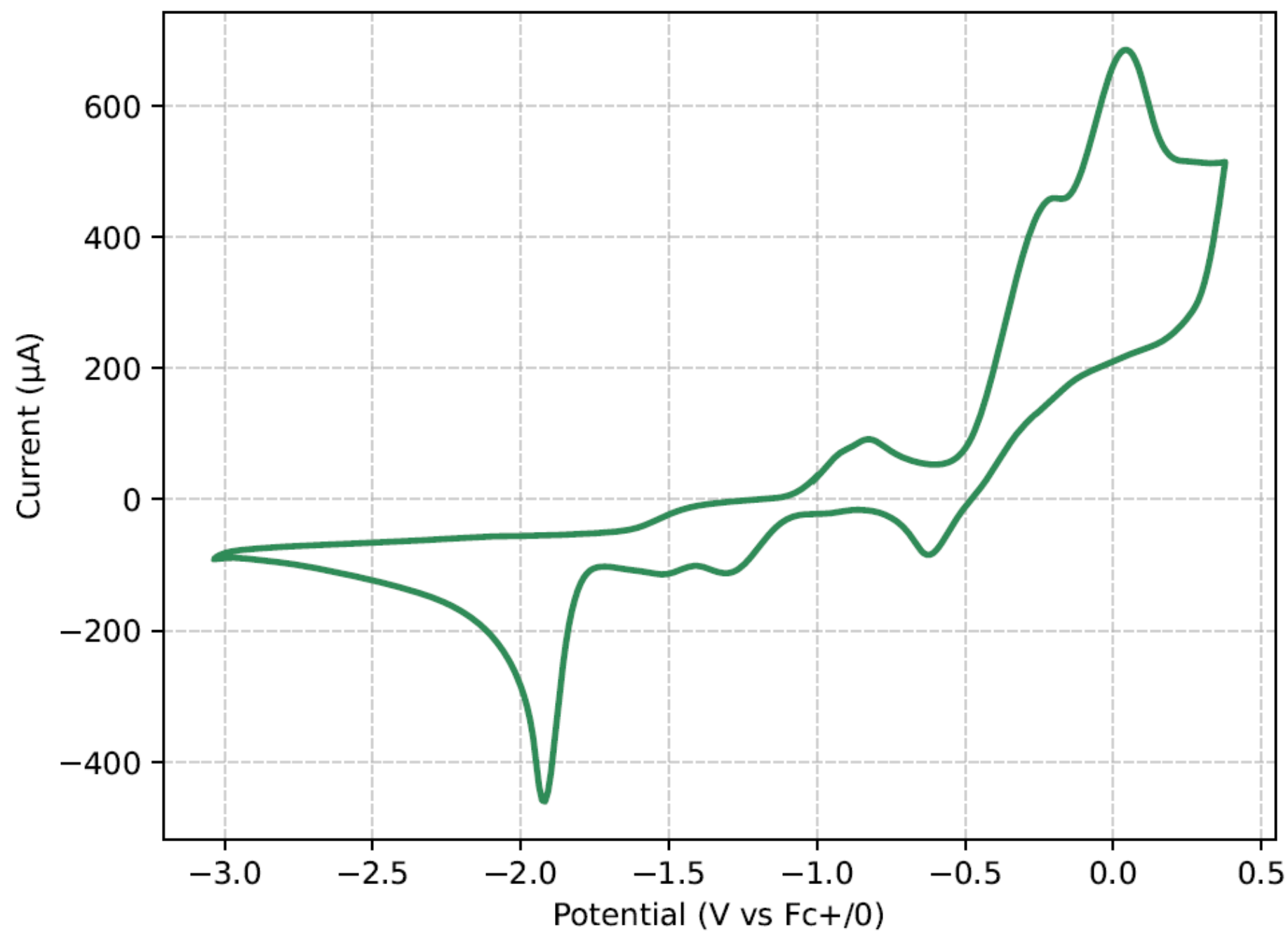

Figure S6. *Cyclic voltammogram of [$Co_2(CO)_8$] (10 mM) and DiPrPF (5 mM, 0.5 equiv.). Conditions: NBu_4PF_6 (0.10 M in DMF). Purged with Ar. Scan rate: 100 mVs^{-1}. Scan direction: Positive direction from 0 V.*

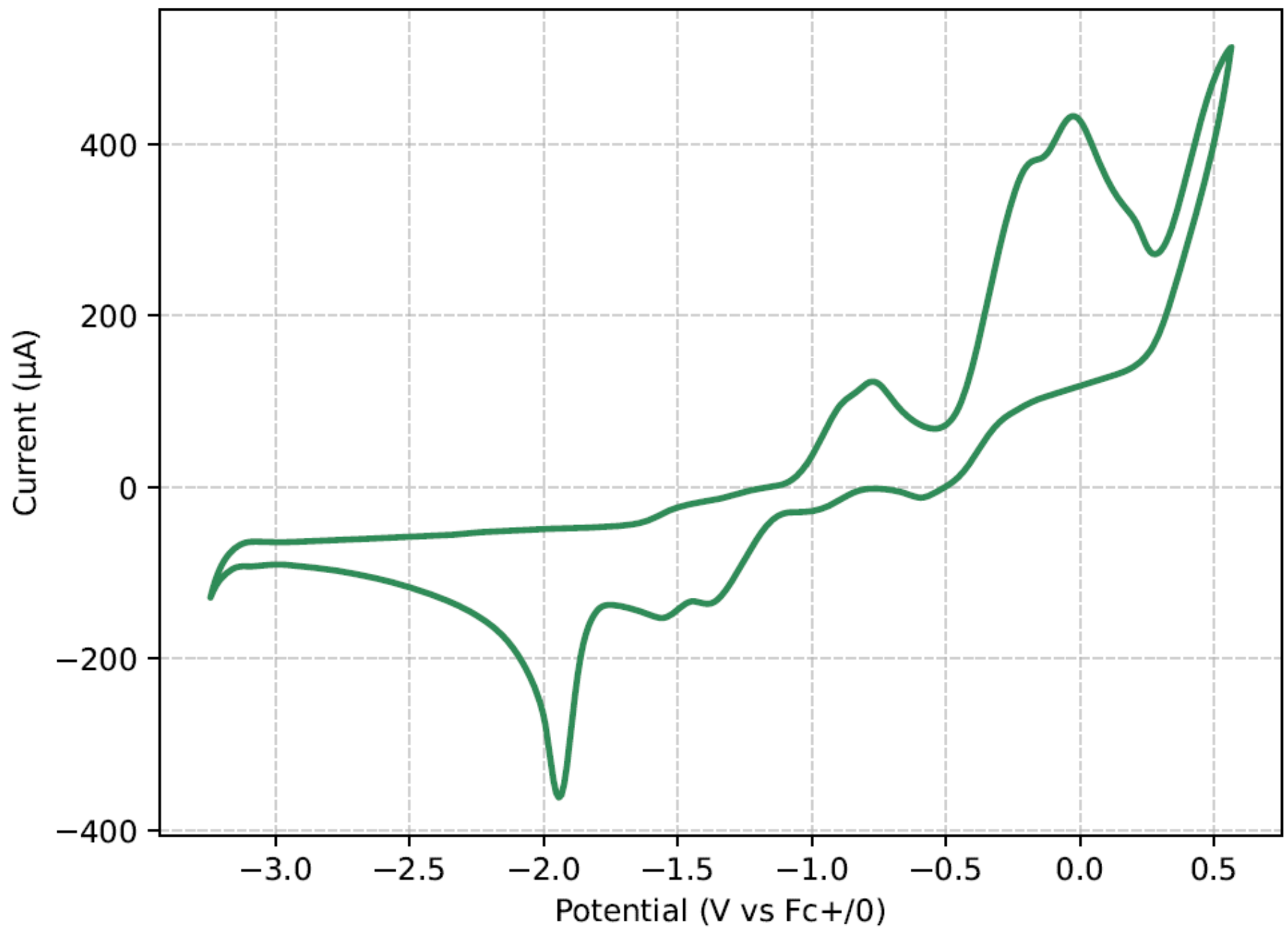

Figure S7. *Cyclic voltammogram of [$Co_2(CO)_8$] (10 mM) and D^{i}PrPF (10 mM, 1 equiv.). Conditions: NBu_4PF_6 (0.10 M in DMF). Purged with Ar. Scan rate: 100 mVs^{-1}. Scan direction: Positive direction from 0 V.*

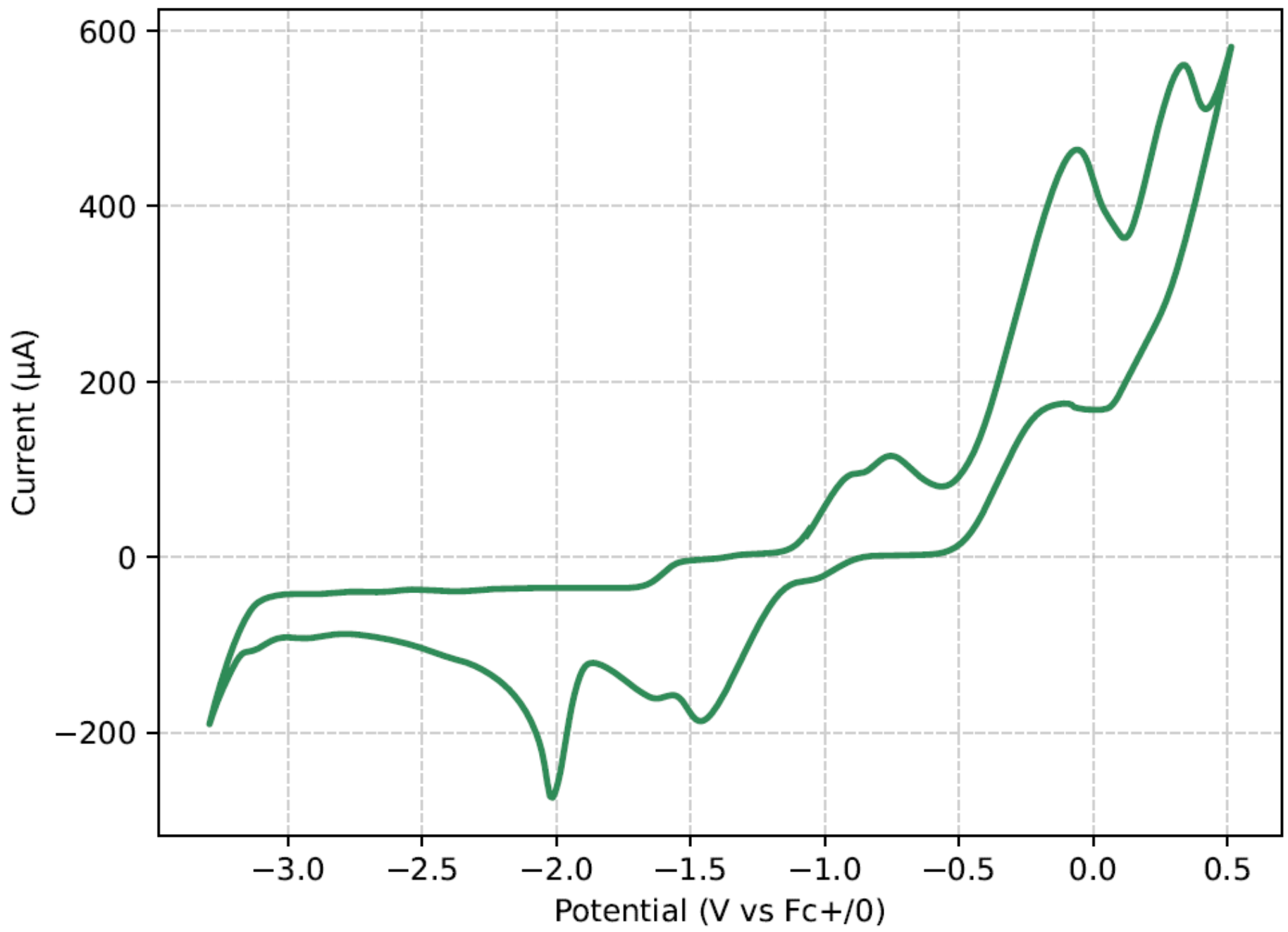

Figure S8. *Cyclic voltammogram of [$Co_2(CO)_8$] (10 mM) and D^{i}PrPF (20 mM, 2 equiv.). Conditions: NBu_4PF_6 (0.10 M in DMF). Purged with Ar. Scan rate: 100 mVs^{-1}. Scan direction: Positive direction from 0 V.*

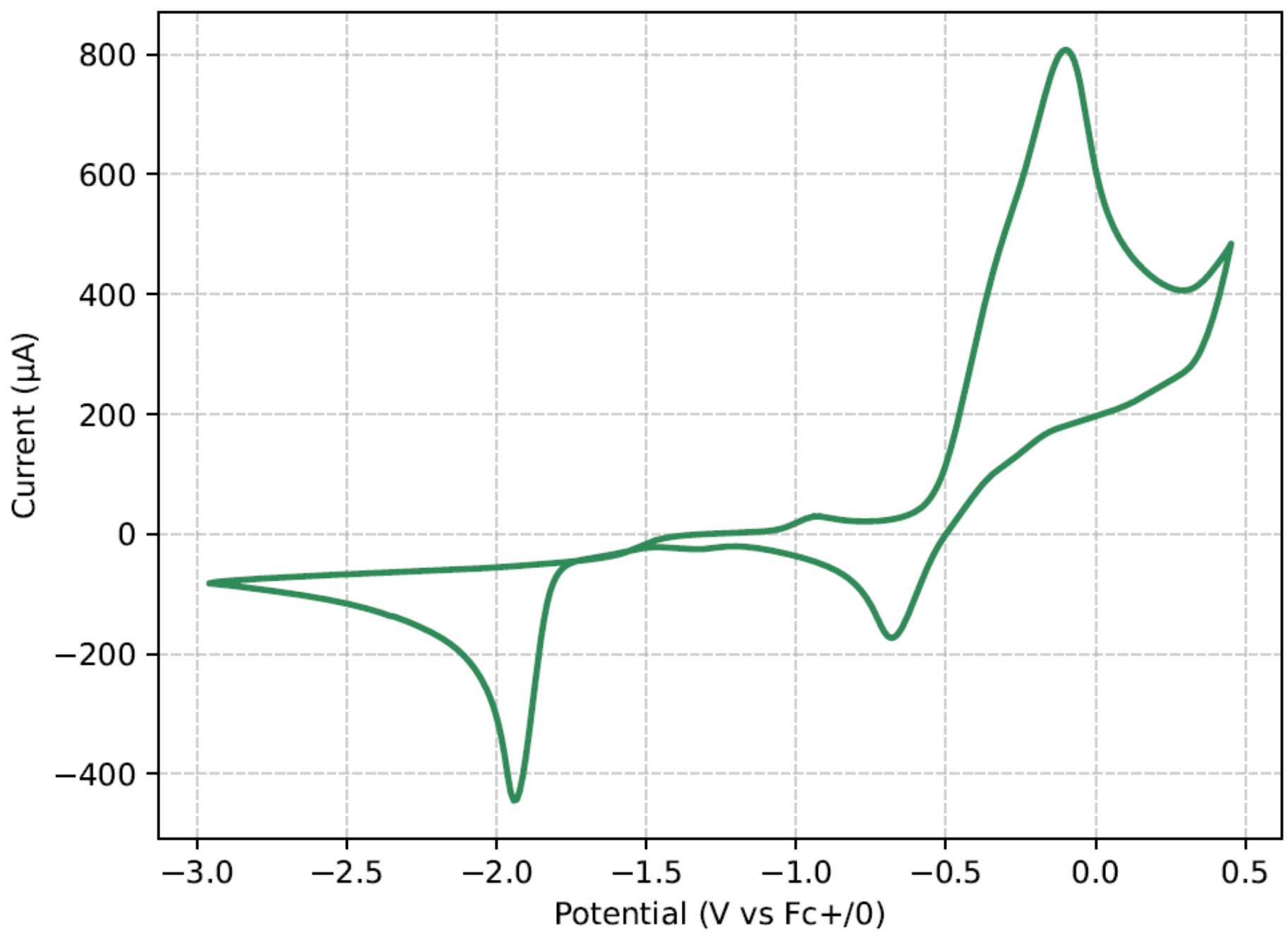

Figure S9. *Cyclic voltammogram of [$Co_2(CO)_8$] (10 mM) and DCyPF (5 mM, 0.5 equiv.). Conditions: NBu_4PF_6 (0.10 M in DMF). Purged with Ar. Scan rate: 100 mVs^{-1}. Scan direction: Positive direction from 0 V.*

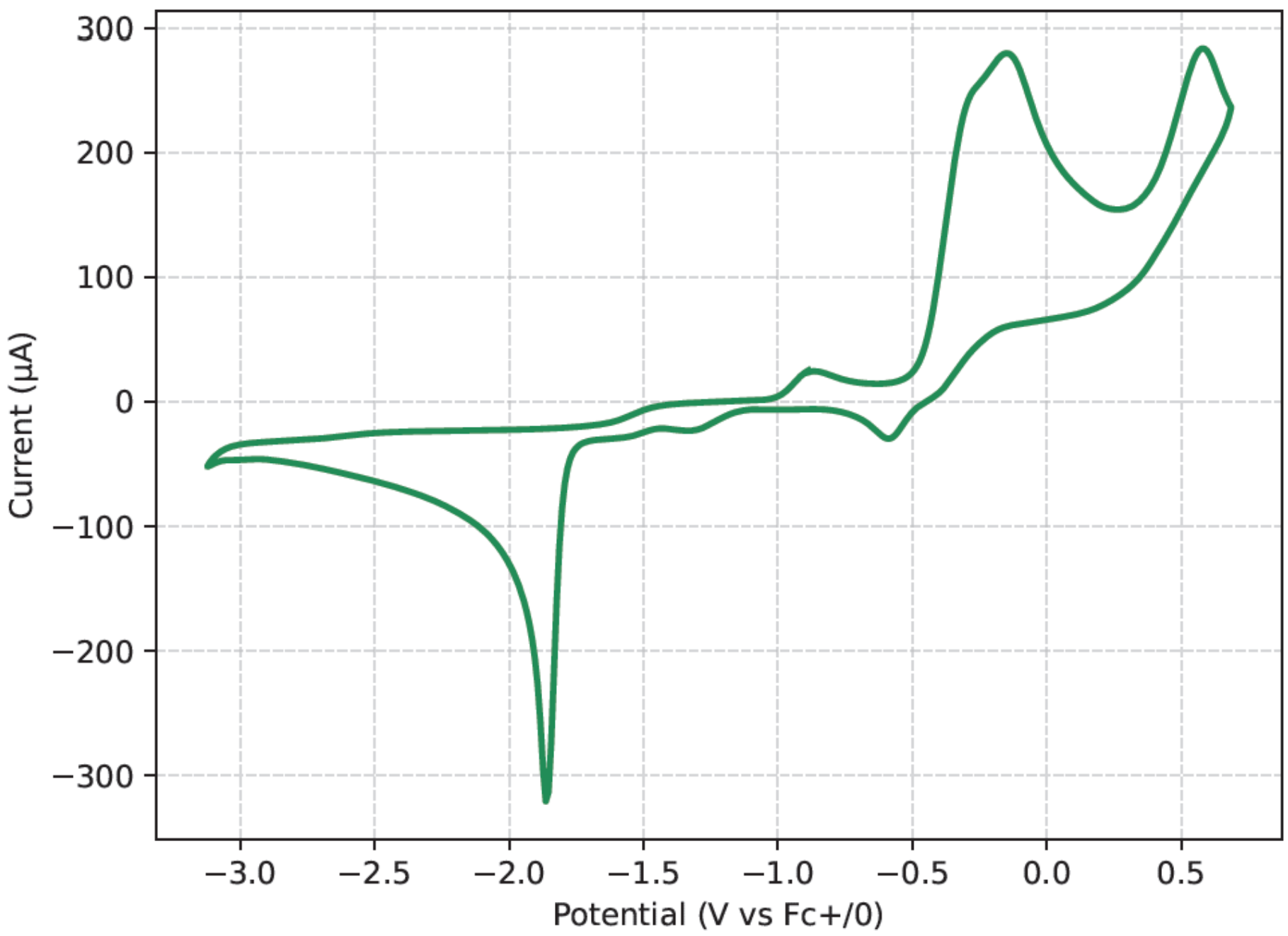

Figure S10. *Cyclic voltammogram of [$Co_2(CO)_8$] (10 mM) and DCyPF (10 mM, 1 equiv.). Conditions: NBu_4PF_6 (0.10 M in DMF). Purged with Ar. Scan rate: 100 mVs^{-1}. Scan direction: Positive direction from 0 V.*

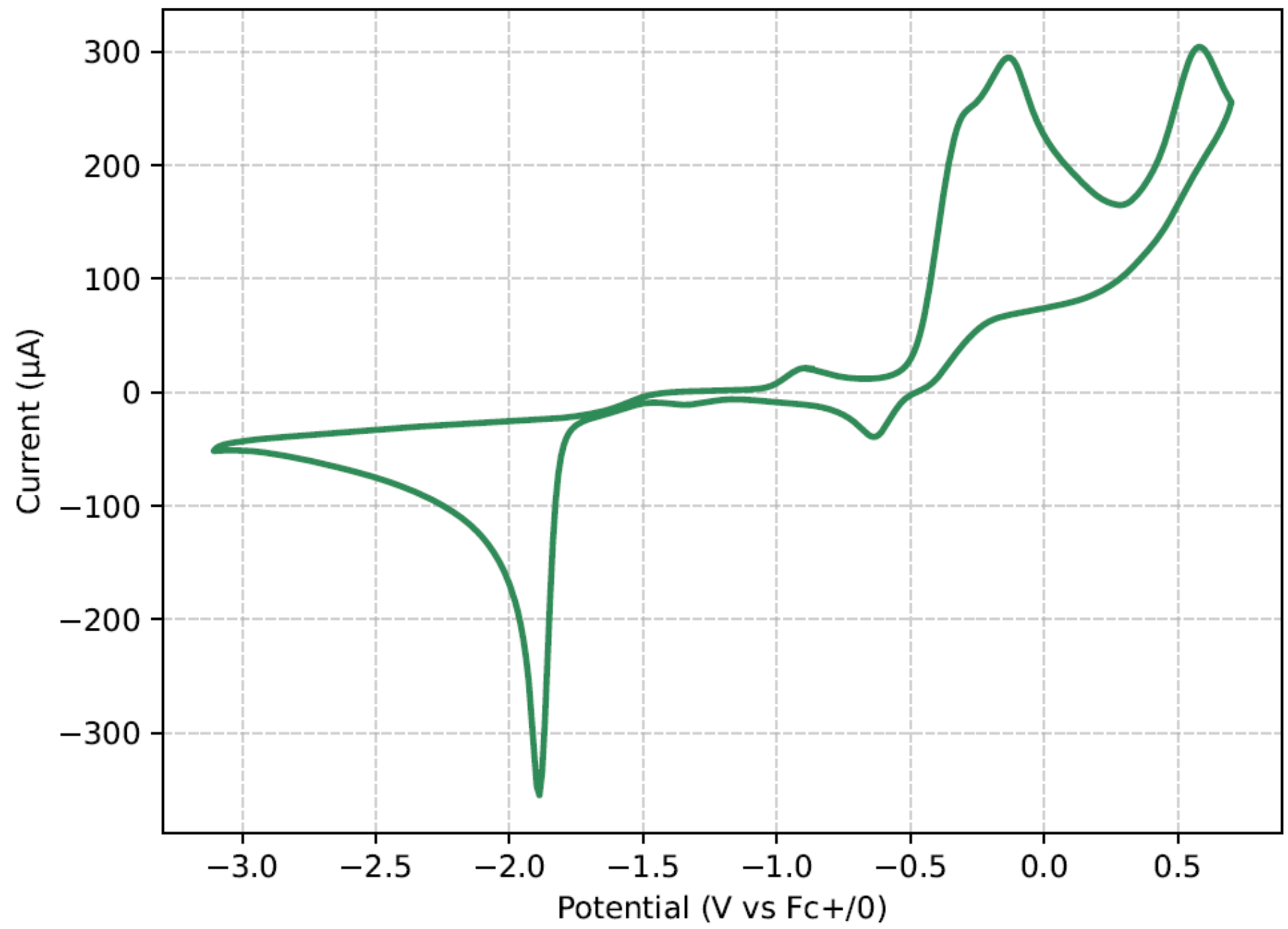

Figure S11. *Cyclic voltammogram of [$Co_2(CO)_8$] (10 mM) and DCyPF (20 mM, 2 equiv.). Conditions: NBu_4PF_6 (0.10 M in DMF). Purged with Ar. Scan rate: 100 mVs^{-1}. Scan direction: Positive direction from 0 V.*

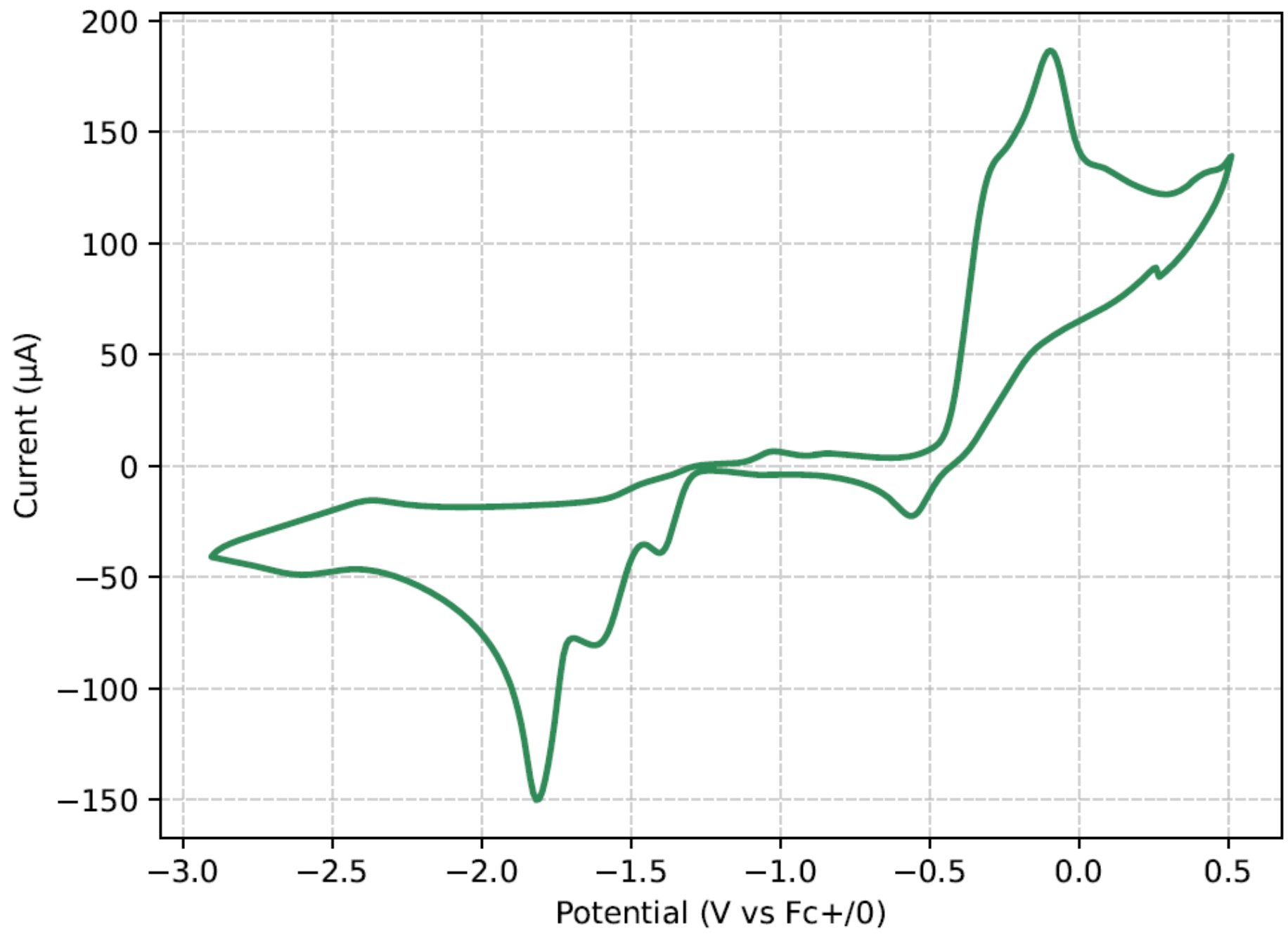

Figure S12. *Cyclic voltammogram of [$Co_2(CO)_8$] (10 mM) and DCPE (10 mM, 1 equiv.). Conditions: NBu_4PF_6 (0.10 M in DMF). Purged with Ar. Scan rate: 100 mVs^{-1}. Scan direction: Positive direction from 0 V.*

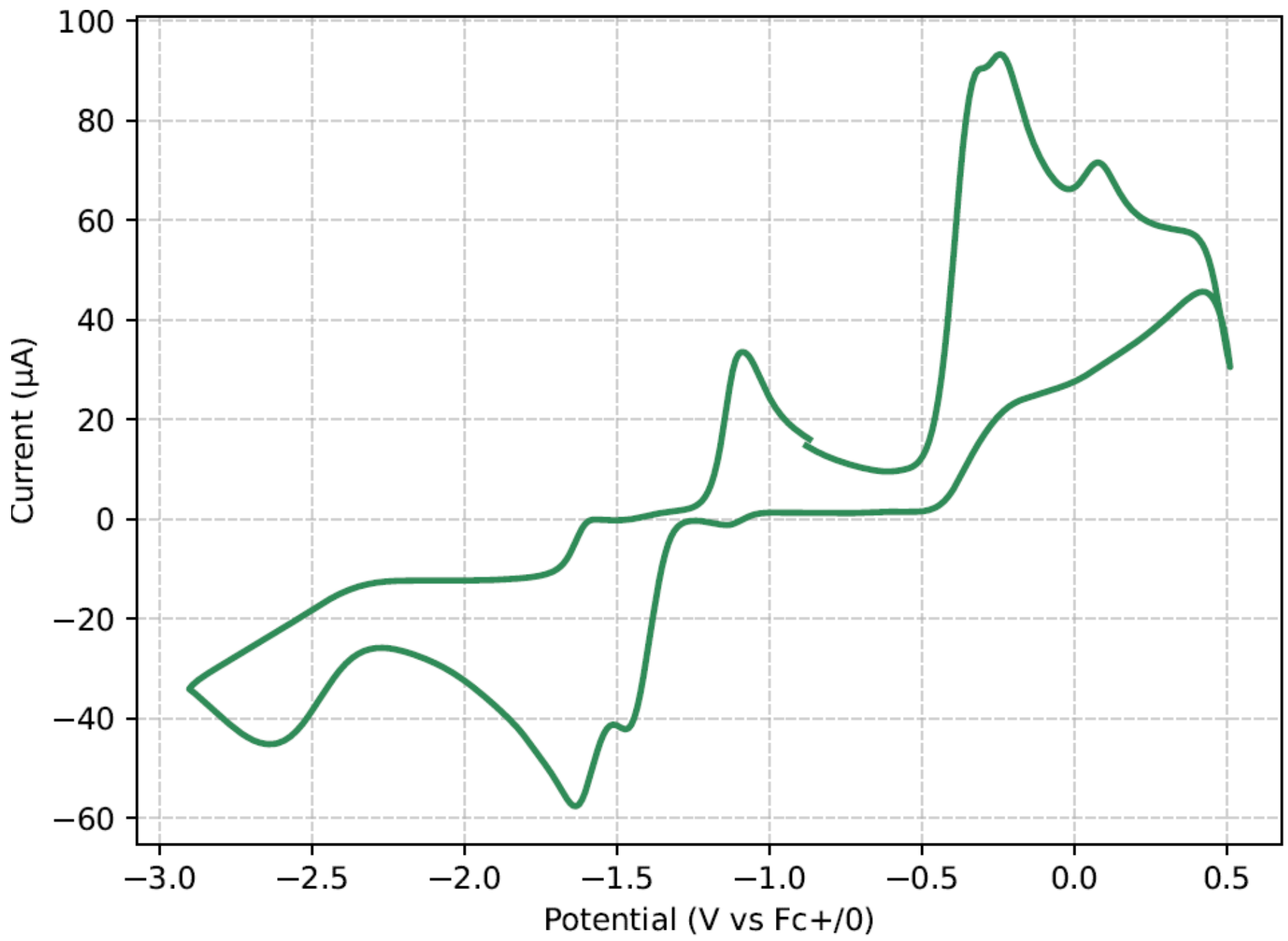

Figure S13. *Cyclic voltammogram of [$Co_2(CO)_8$] (10 mM) and DCPE (20 mM, 2 equiv.). Conditions: NBu_4PF_6 (0.10 M in DMF). Purged with Ar. Scan rate: 100 mVs^{-1}. Scan direction: Positive direction from 0 V.*

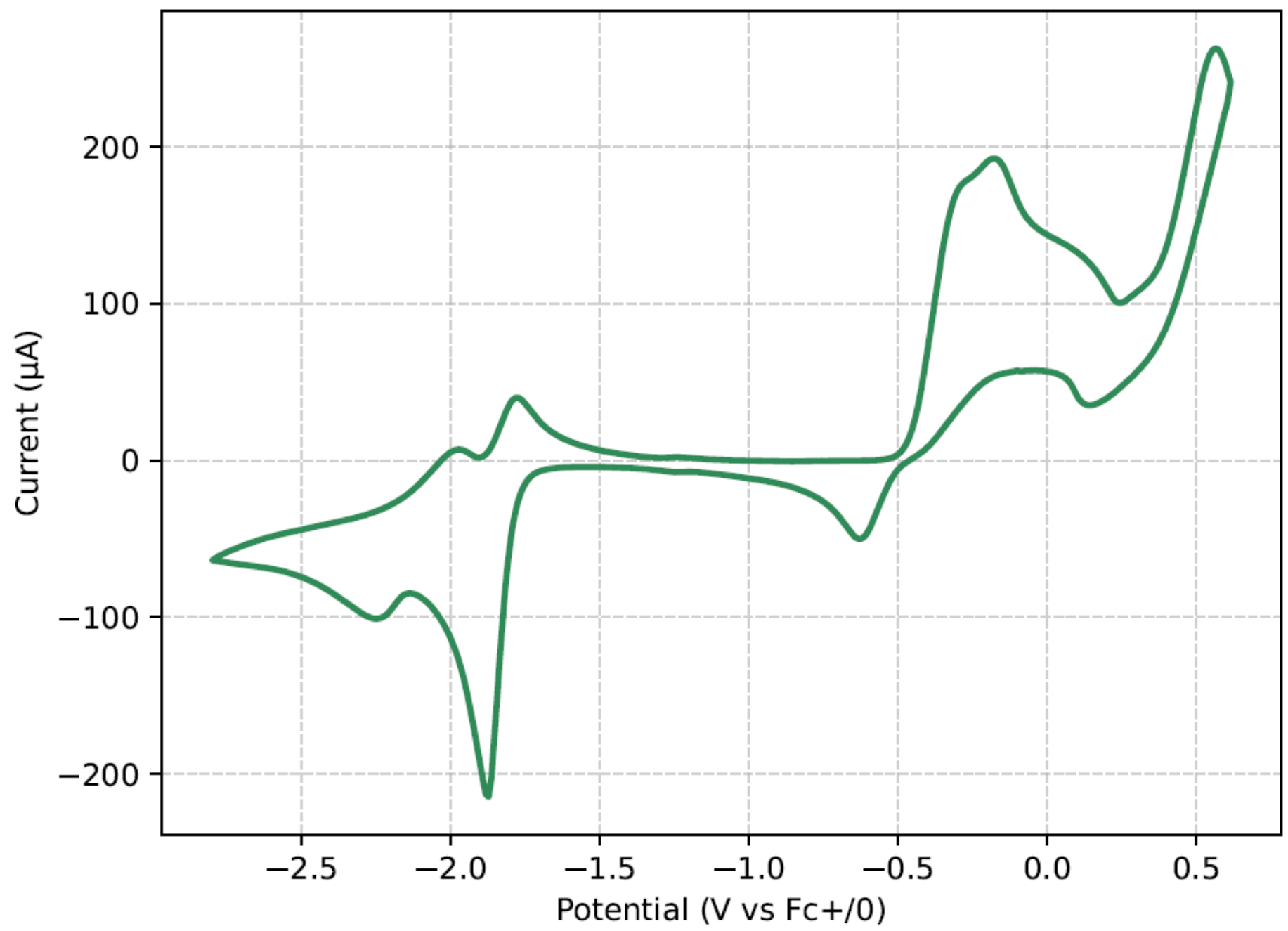

Figure S14. *Cyclic voltammogram of [$Co_2(CO)_8$] (10 mM) and DPPBz (10 mM, 1 equiv.). Conditions: NBu_4PF_6 (0.10 M in DMF). Purged with Ar. Scan rate: 100 mVs^{-1}. Scan direction: Positive direction from 0 V.*

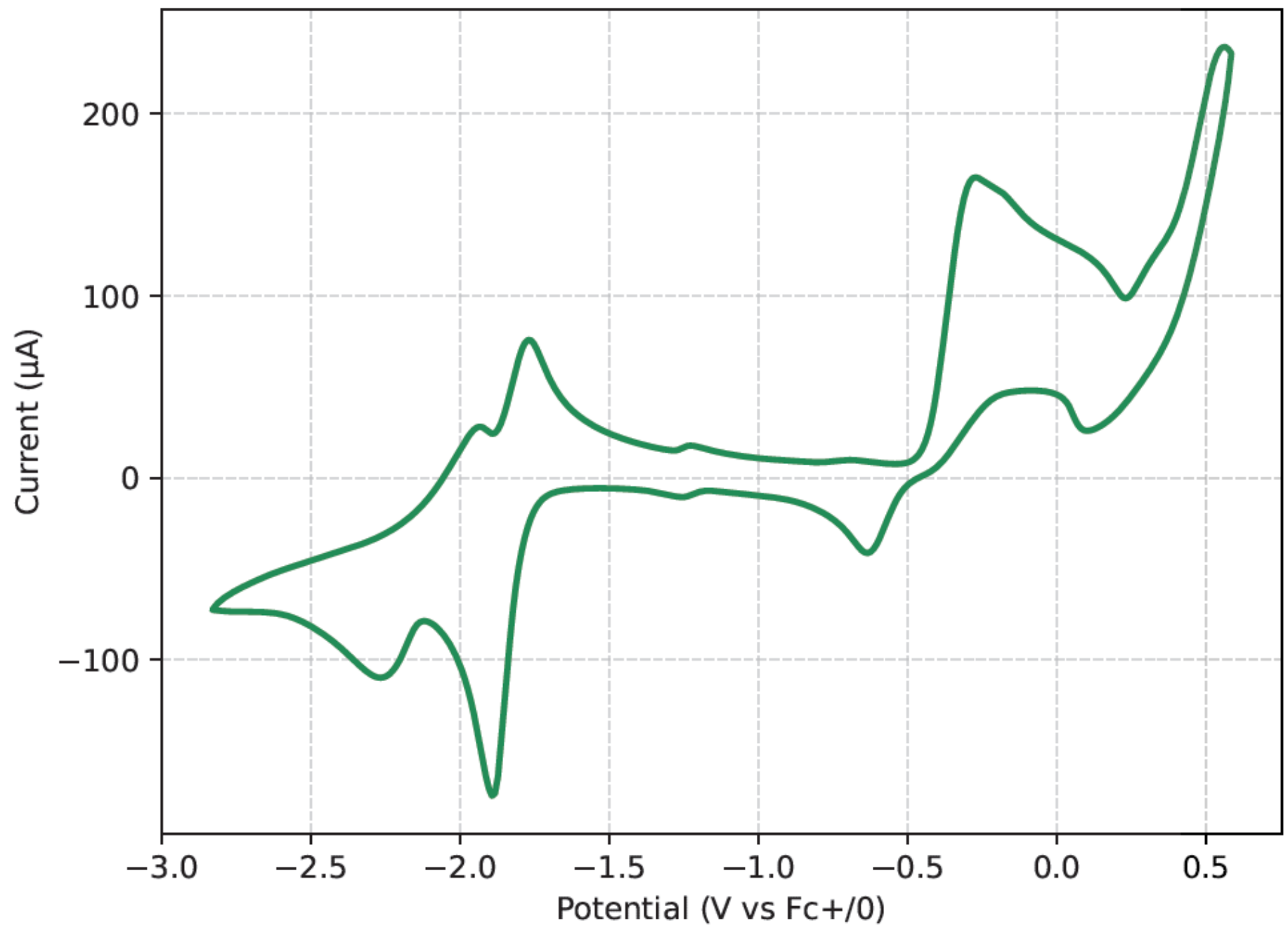

Figure S15. *Cyclic voltammogram of [$Co_2(CO)_8$] (10 mM) and DPPBz (20 mM, 2 equiv.). Conditions: NBu_4PF_6 (0.10 M in DMF). Purged with Ar. Scan rate: 100 mVs^{-1}. Scan direction: Positive direction from 0 V.*

In-Situ NMR Spectra

Complexes using the DCPE Ligand

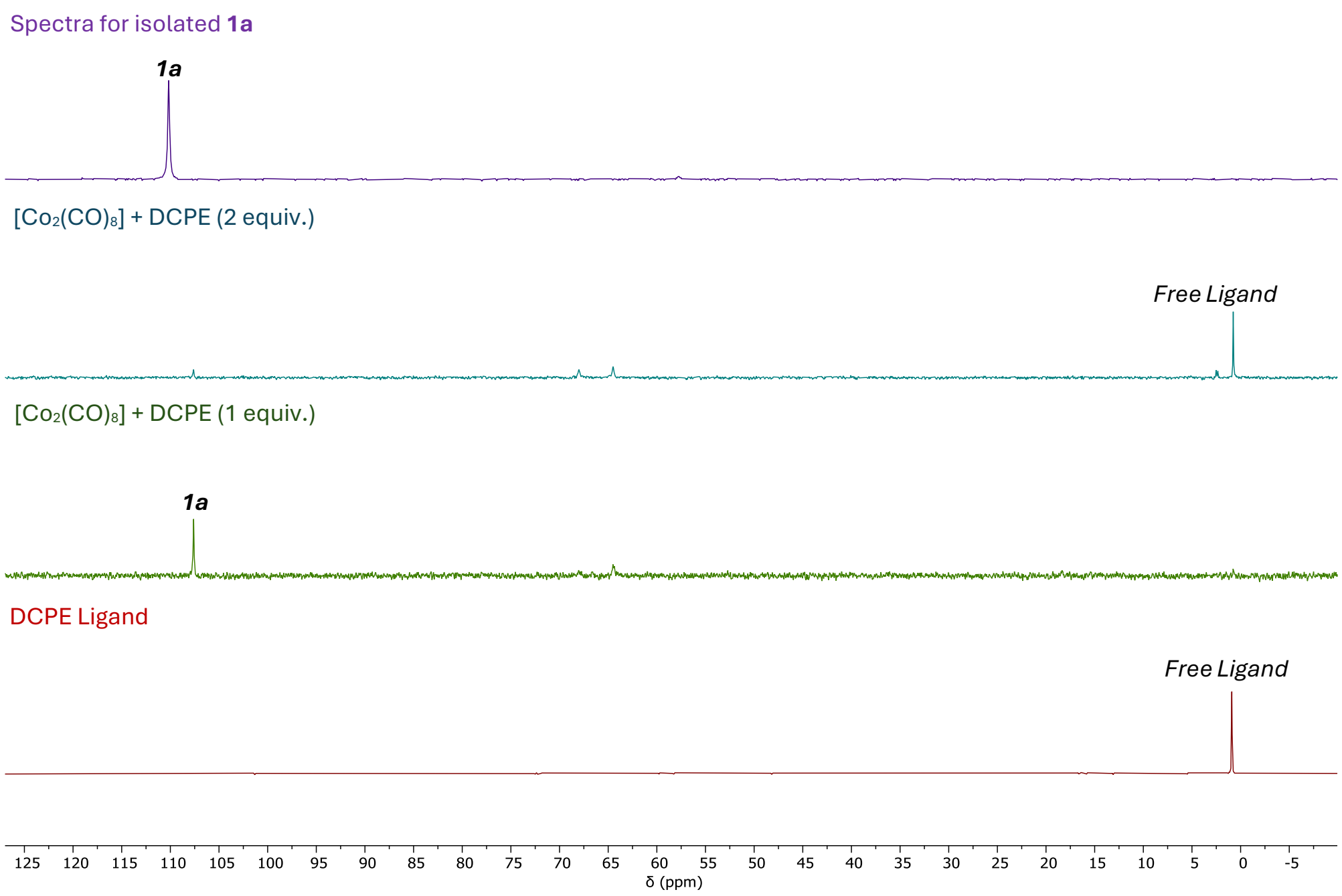

Figure S16. *^{31}P NMR spectra recorded in THF solvent for free ligand DCPE and various ratios of ligand:[$Co_2(CO)_8$].*

Complexes using the DPPBz Ligand

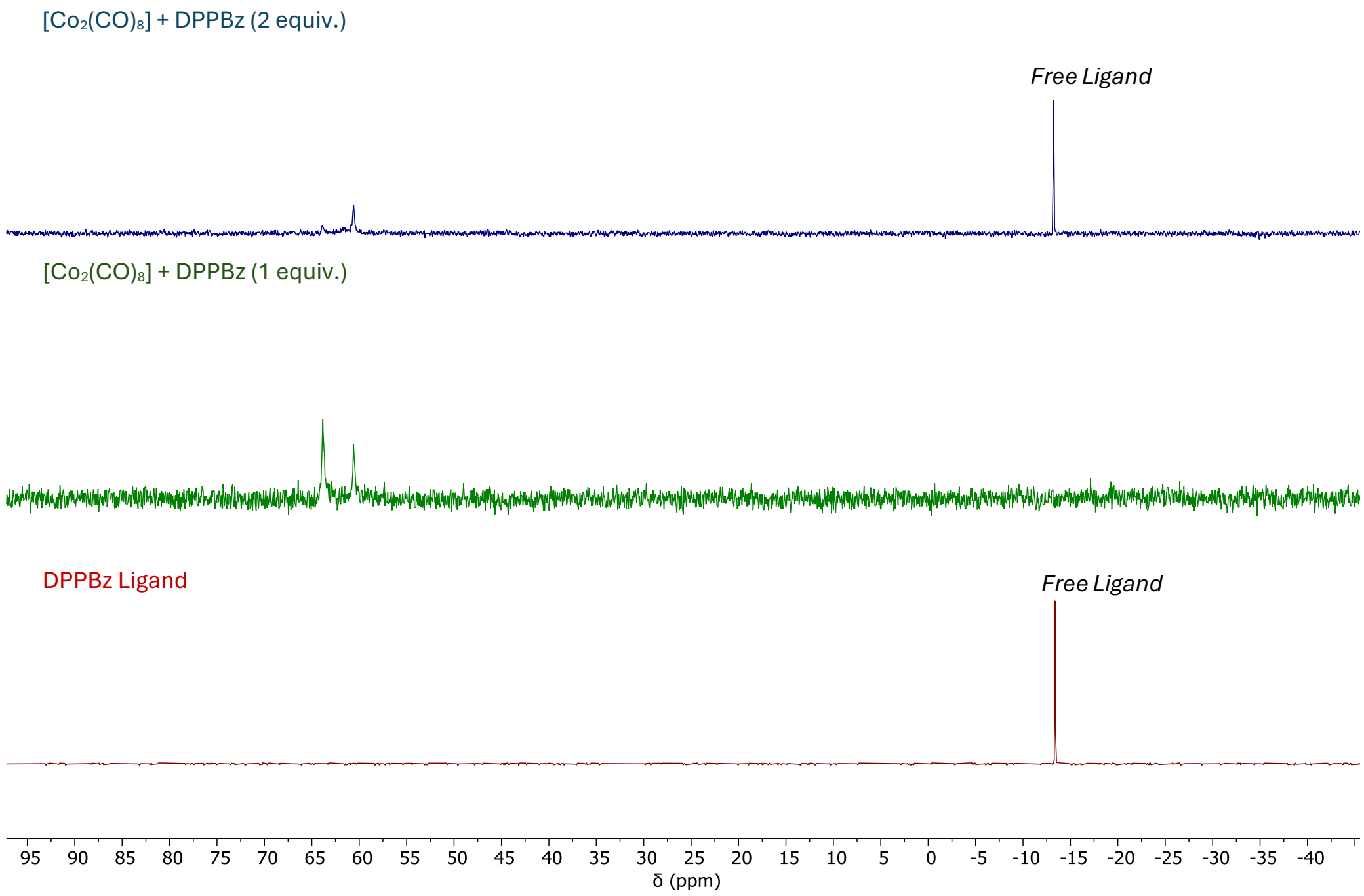

Figure S17. *^{31}P NMR spectra recorded in THF solvent for free ligand DPPBz and various ratios of ligand:[$Co_2(CO)_8$].*

Complexes using the DPPF ligand

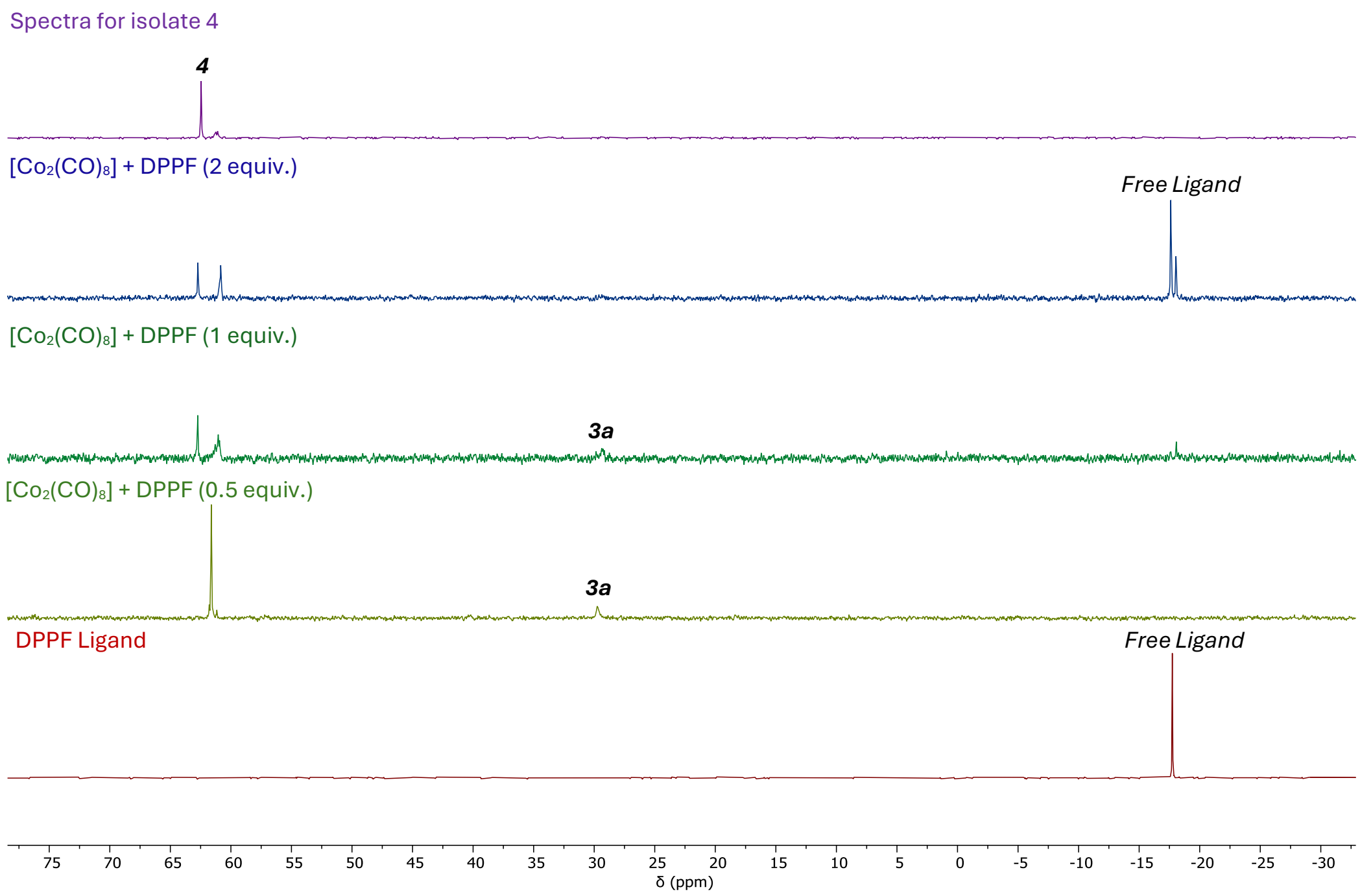

Figure S18. *^{31}P NMR spectra recorded in THF solvent for free ligand DPPF and various ratios of ligand:[$Co_2(CO)_8$].*

Complexes using the D^{i}PrPF Ligand

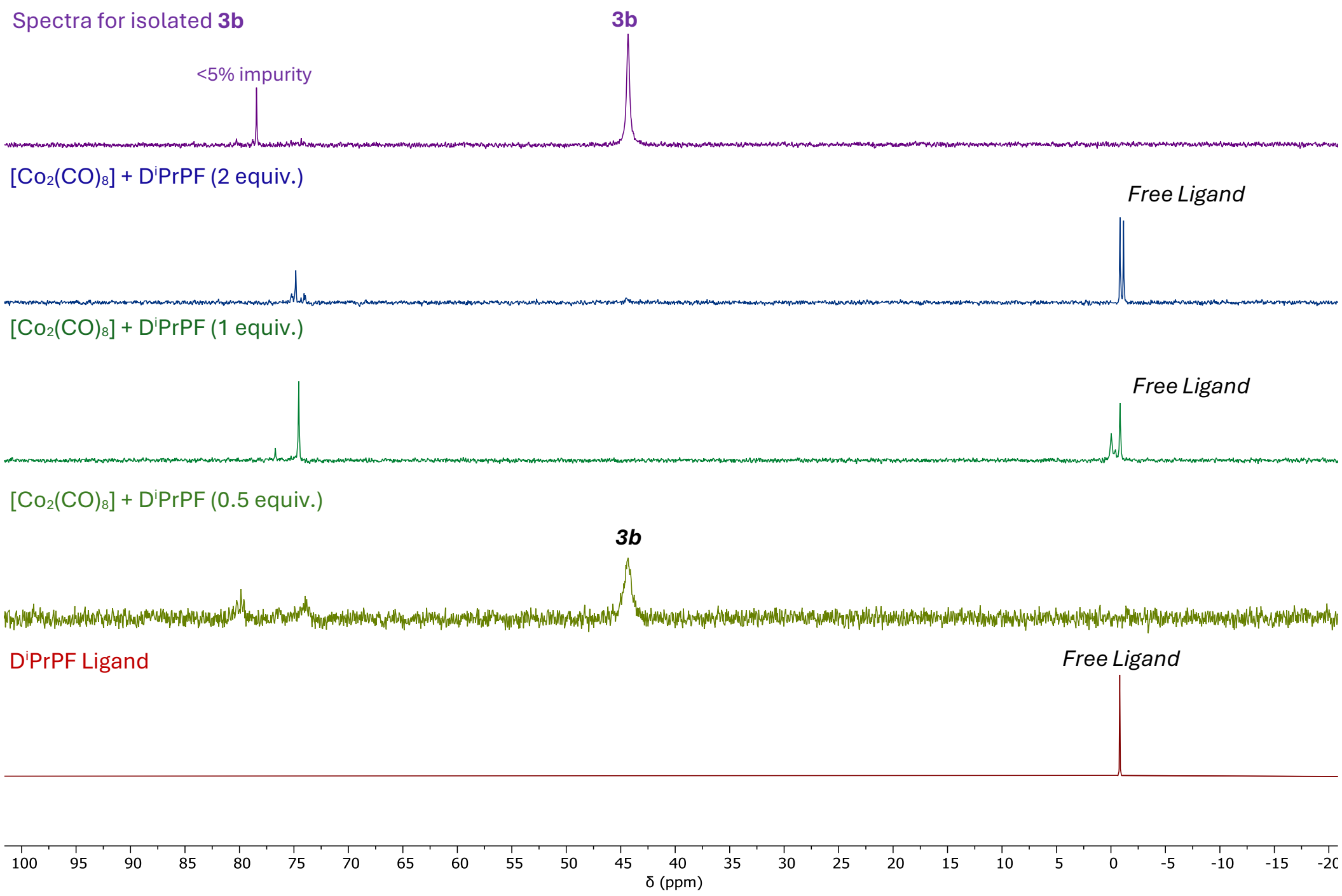

Figure S19. *^{31}P NMR spectra recorded in THF solvent for free ligand DiPrPF and various ratios of ligand:[$Co_2(CO)_8$].*

Complexes using the DCyPF Ligand

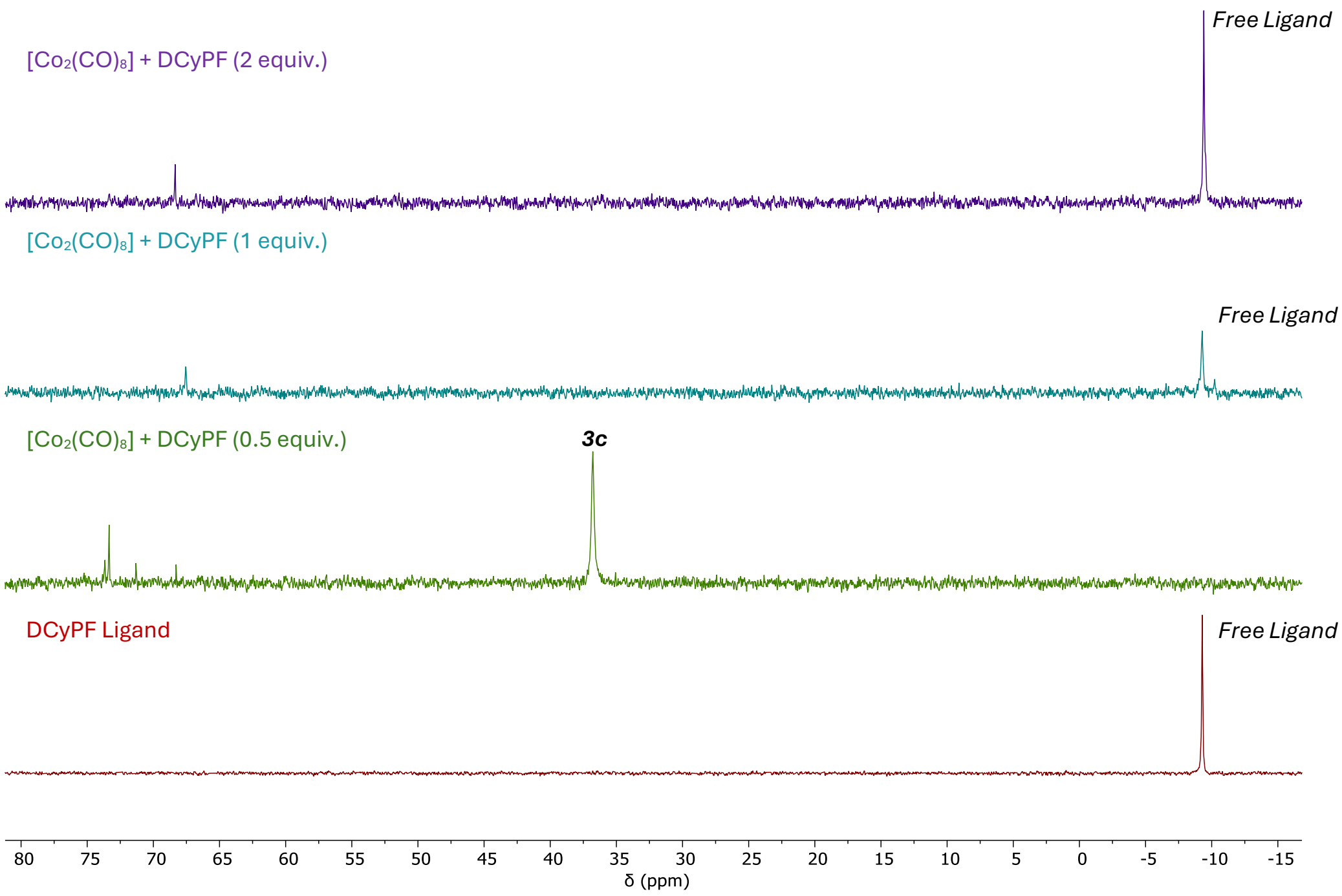

Figure S20. *^{31}P NMR spectra recorded in THF solvent for free ligand DCyPF and various ratios of ligand:[$Co_2(CO)_8$]. Loss of product peak intensity is aligned with insoluble polymer build up at higher ligand loadings.*

FT-IR Analysis

Complexes using the DCPE Ligand

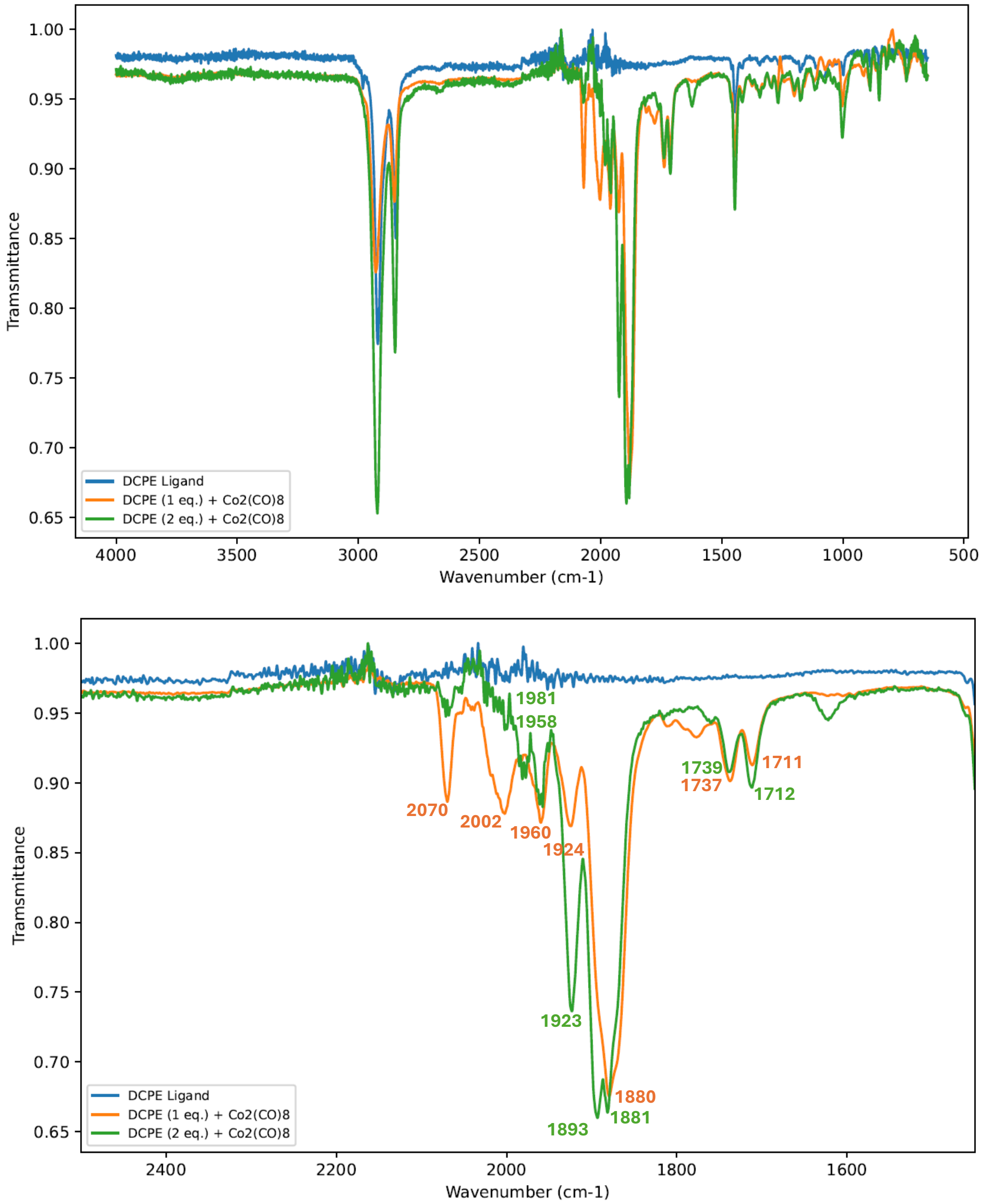

Figure S21. *IR spectra of varied loading DCPE additions to [$Co_2(CO)_8$]. Full spectra (top), carbonyl zoomed spectra (bottom).*

Complexes using the DPPBz Ligand

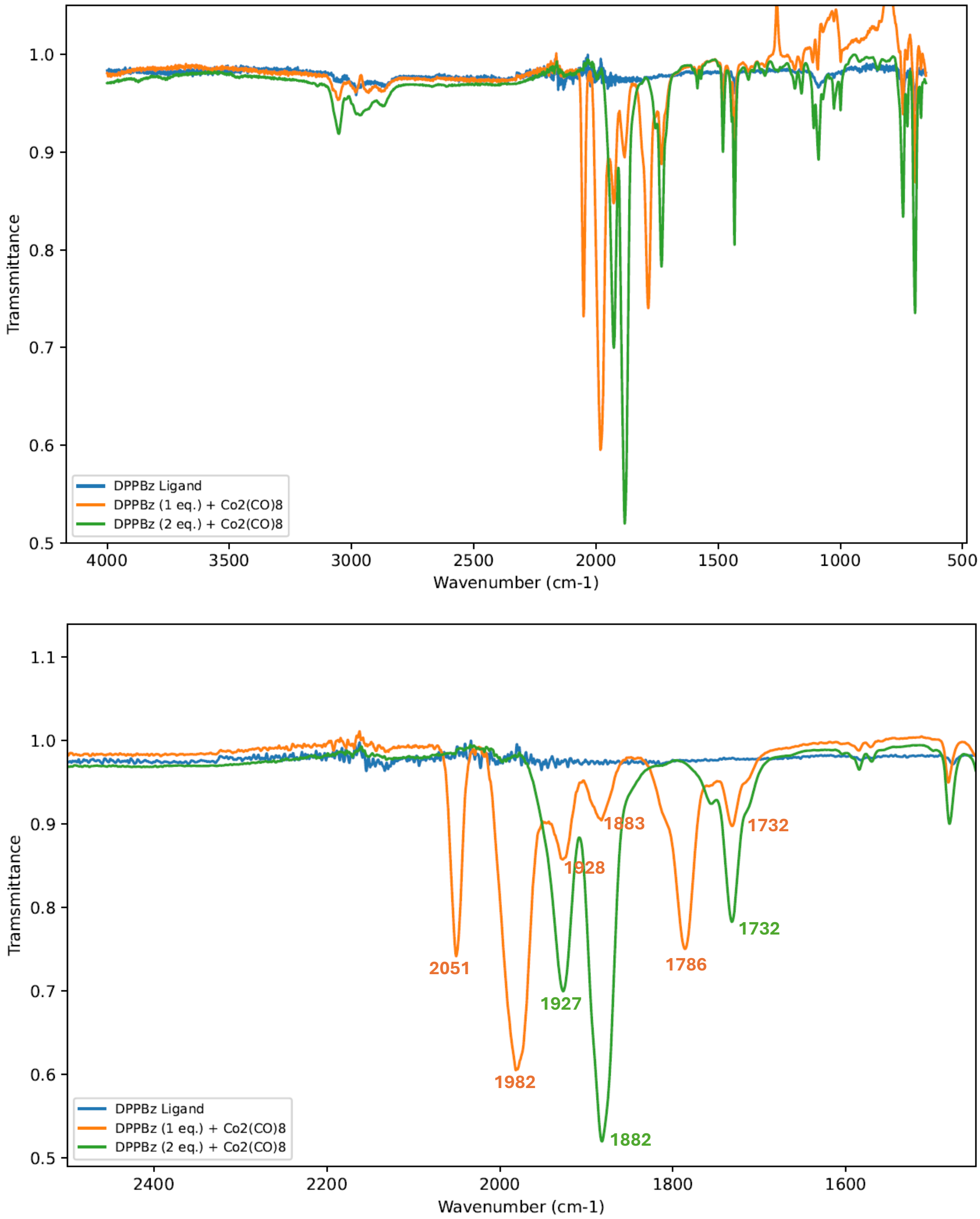

Figure S22. *IR spectra of varied loading DPPBz additions to [$Co_2(CO)_8$].*

Complexes using the DPPF Ligand

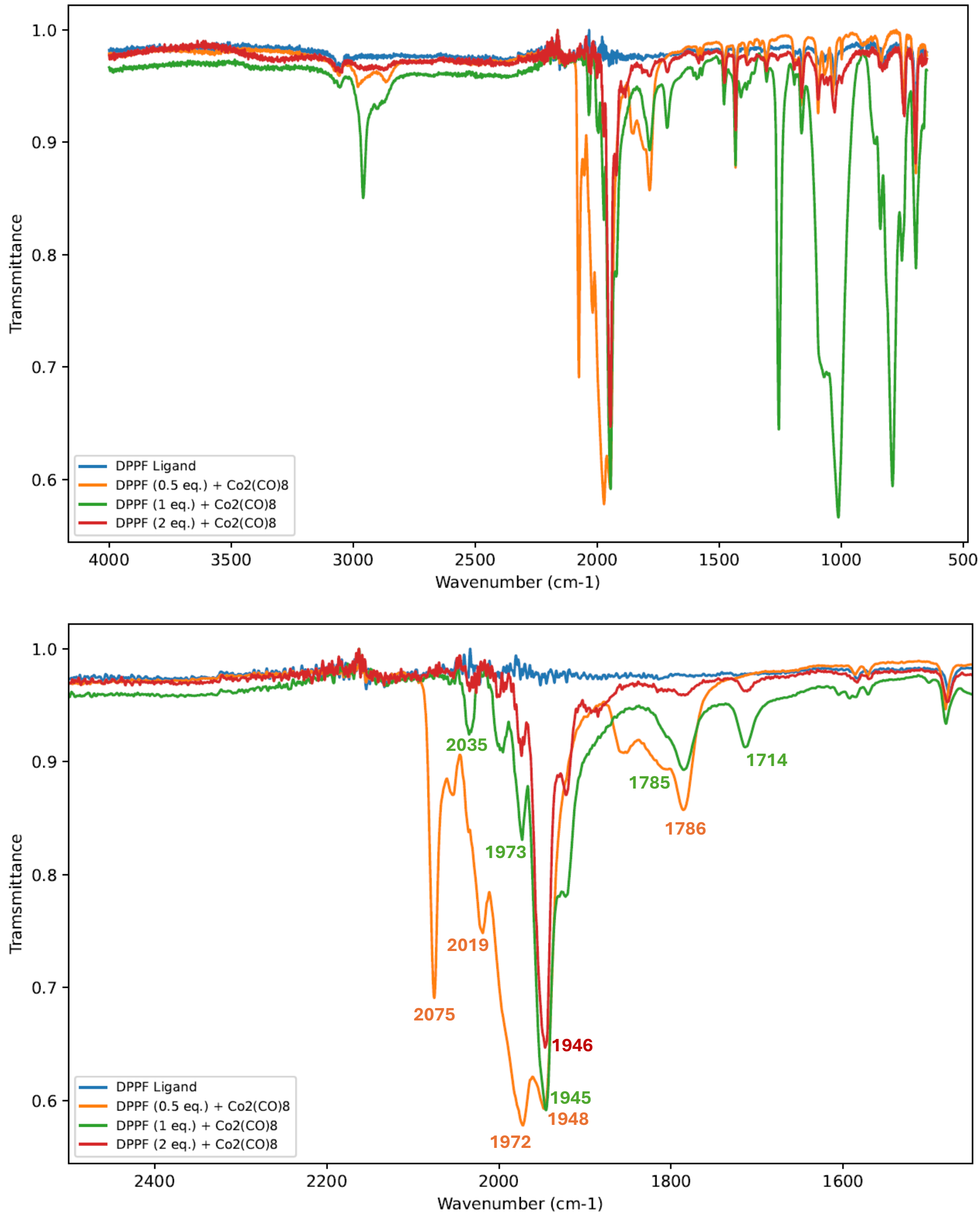

Figure S23. *IR spectra of varied loading DPPF additions to [$Co_2(CO)_8$]. Full spectra (top), carbonyl zoomed spectra (bottom). Due to solubility, removal of residual THF from film preparation was not possible for the green trace shown.*

Complexes using the D^{i}PrPF Ligand

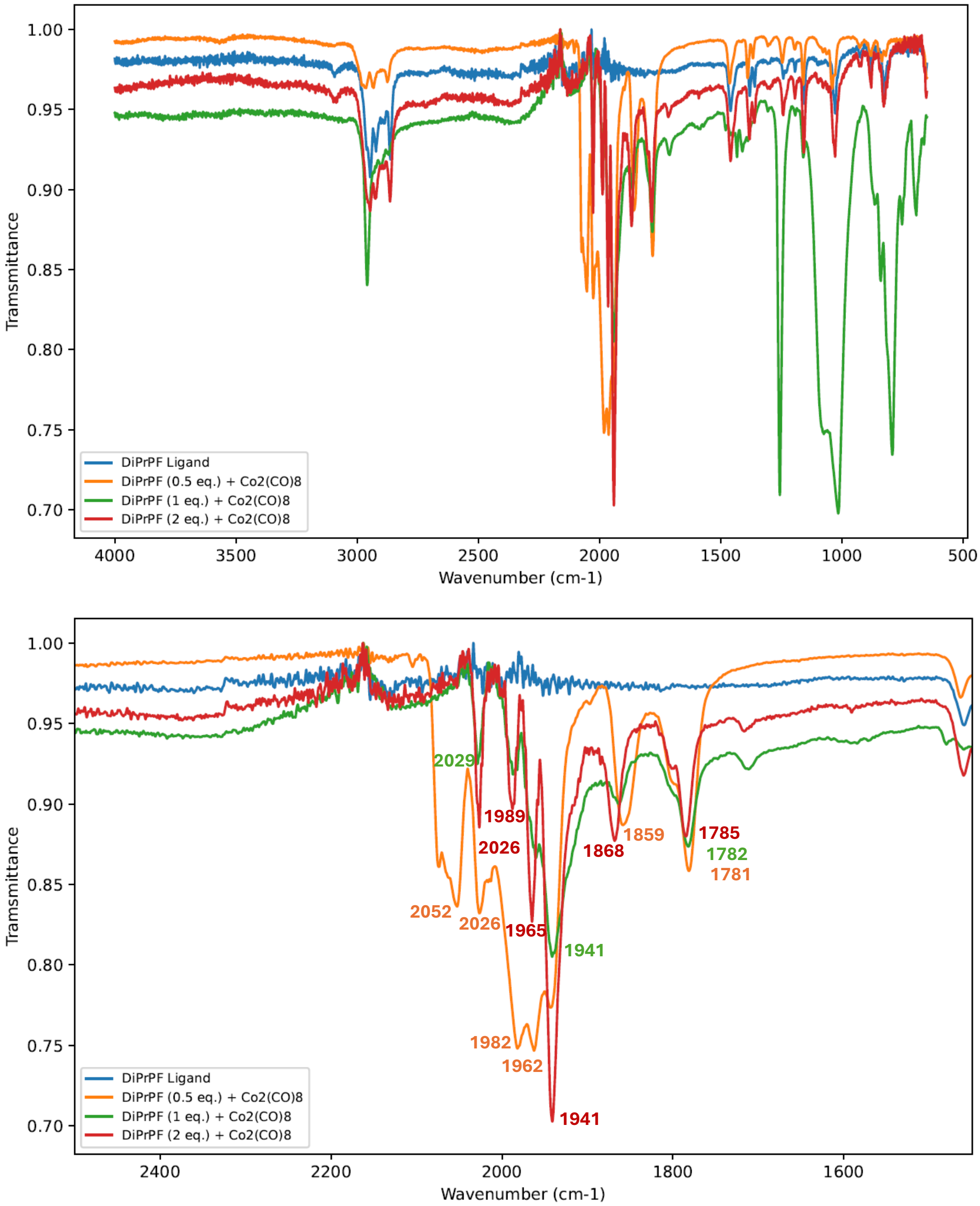

Figure S24. *IR spectra of varied loading DiPrPF additions to [$Co_2(CO)_8$]. Full spectra (top), carbonyl zoomed spectra (bottom). Due to solubility, removal of residual THF from film preparation was not possible for the green trace shown.*

Complexes using the DCyPF Ligand

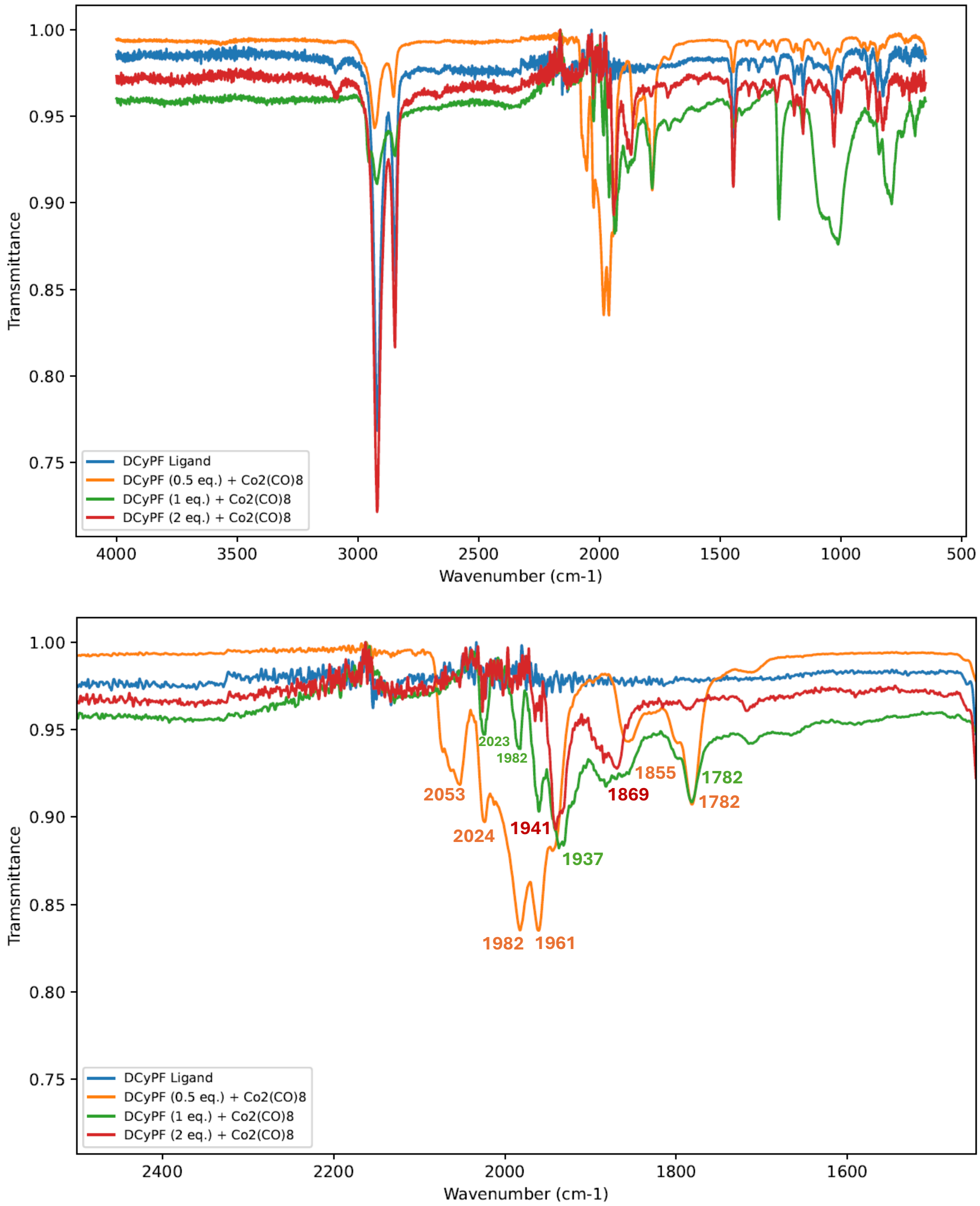

Figure S25. *IR spectra of varied loading DCyPF additions to [$Co_2(CO)_8$]. Full spectra (top), carbonyl zoomed spectra (bottom). Due to solubility limitations, lower intensity signals were obtained following film preparation*

NMR Spectra for Isolated Compounds

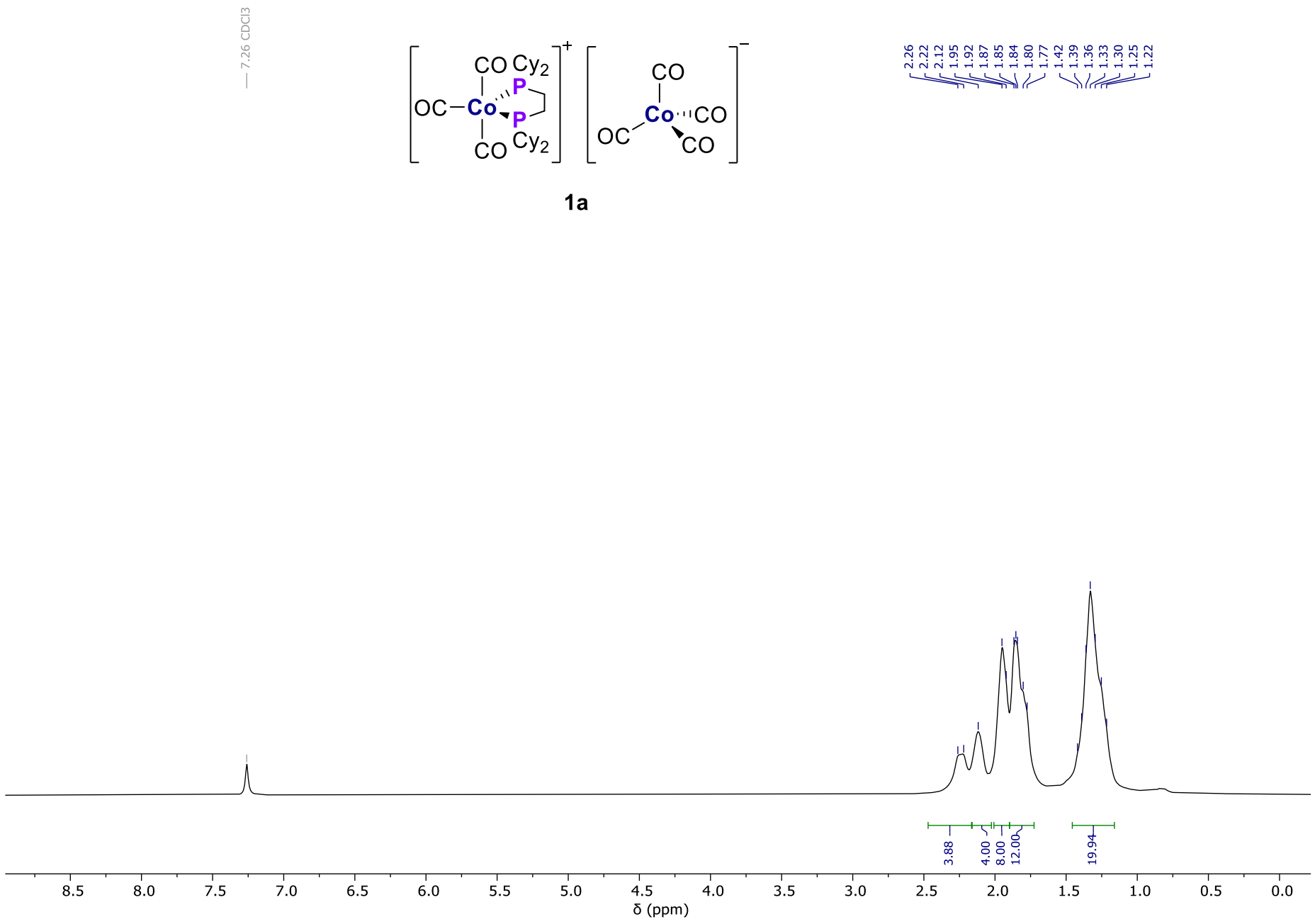

1a

Figure S26. *^{1}H NMR spectra for **1a** (400 MHz, CD_2Cl_2, 298 K).*

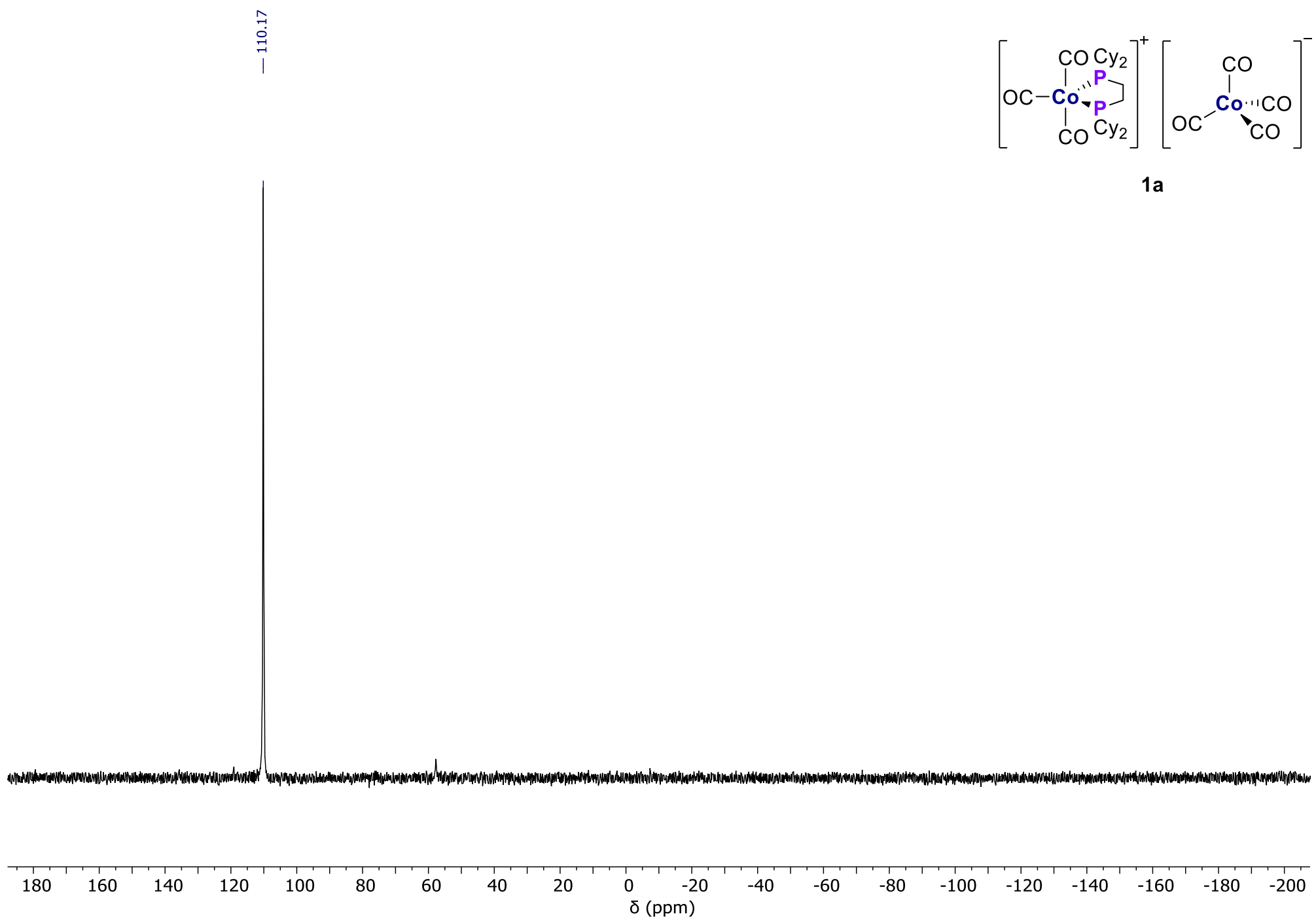

1a

Figure S27. *^{31}P NMR spectra for **1a** (162 MHz, CD_2Cl_2, 298 K).*

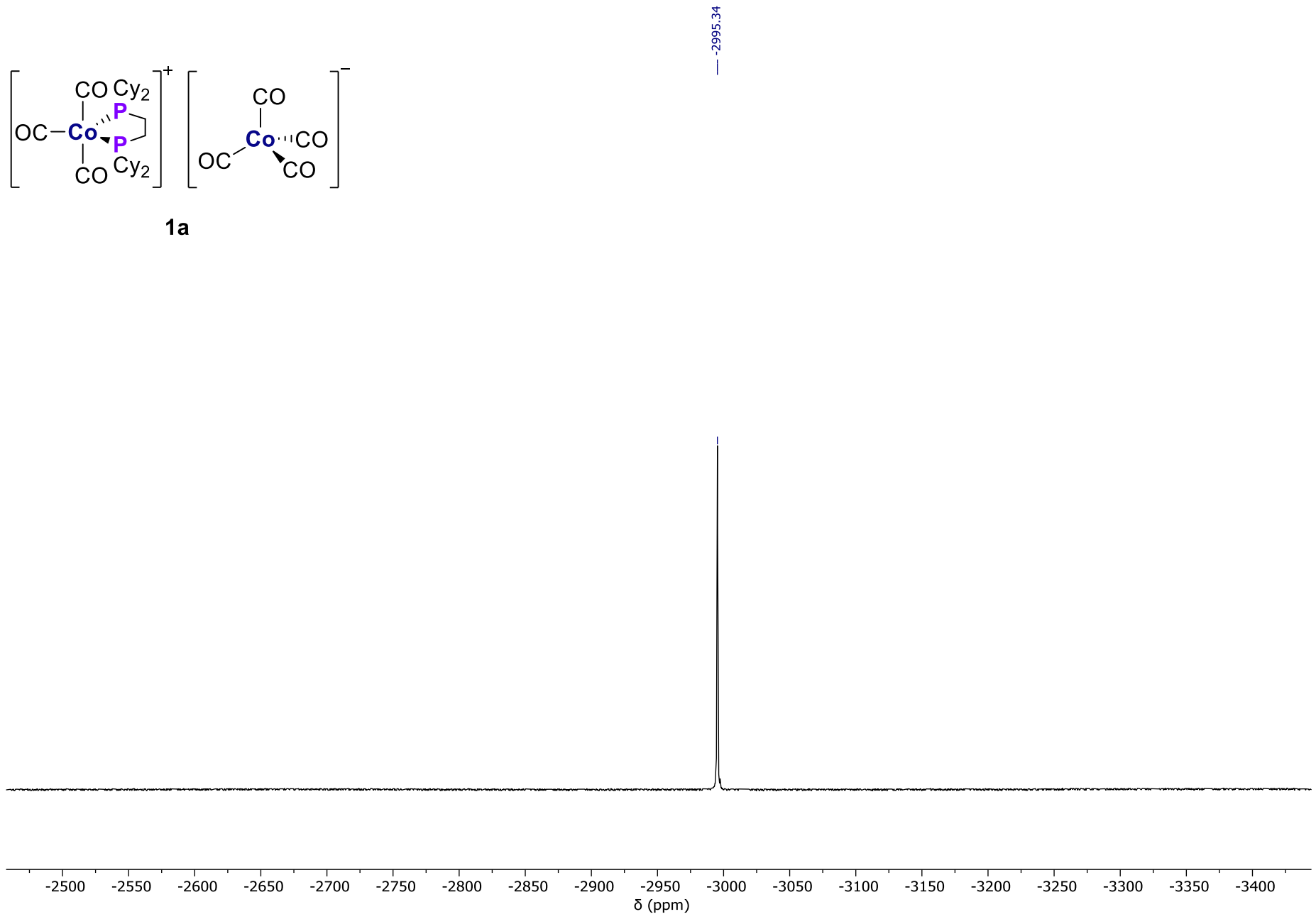

Figure S28. *^{59}Co NMR spectra for **1a** (94.9 MHz, CD_2Cl_2, 298 K).*

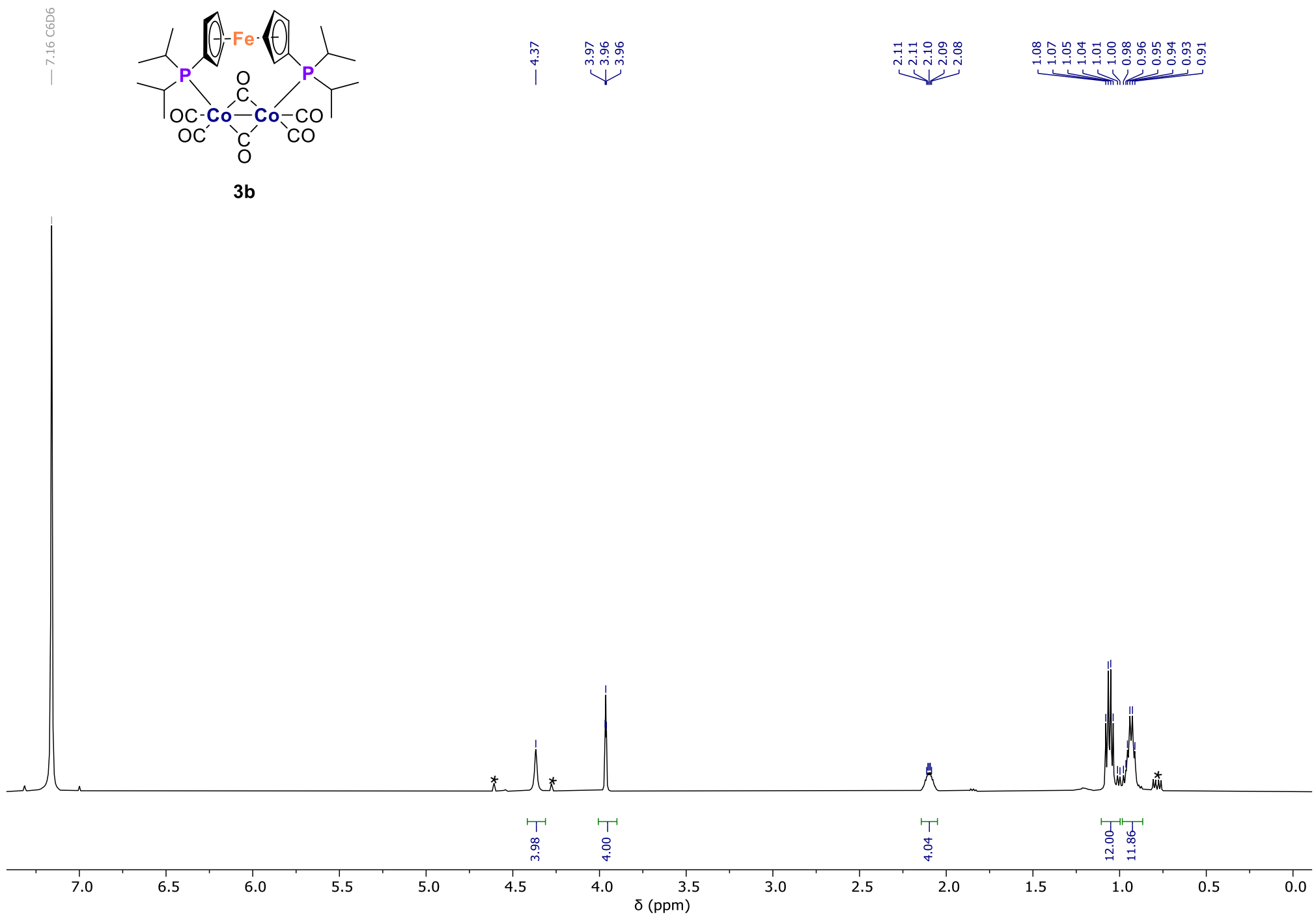

Figure S29. *^{1}H NMR spectra for **3b** (400 MHz, C_6D_6, 298 K). * Evidence of side product from reaction mixture (<5%). DOSY analysis confirms this as 'oversubstituted complex' likely bearing 2 equivalents of D^{i}PrPF.*

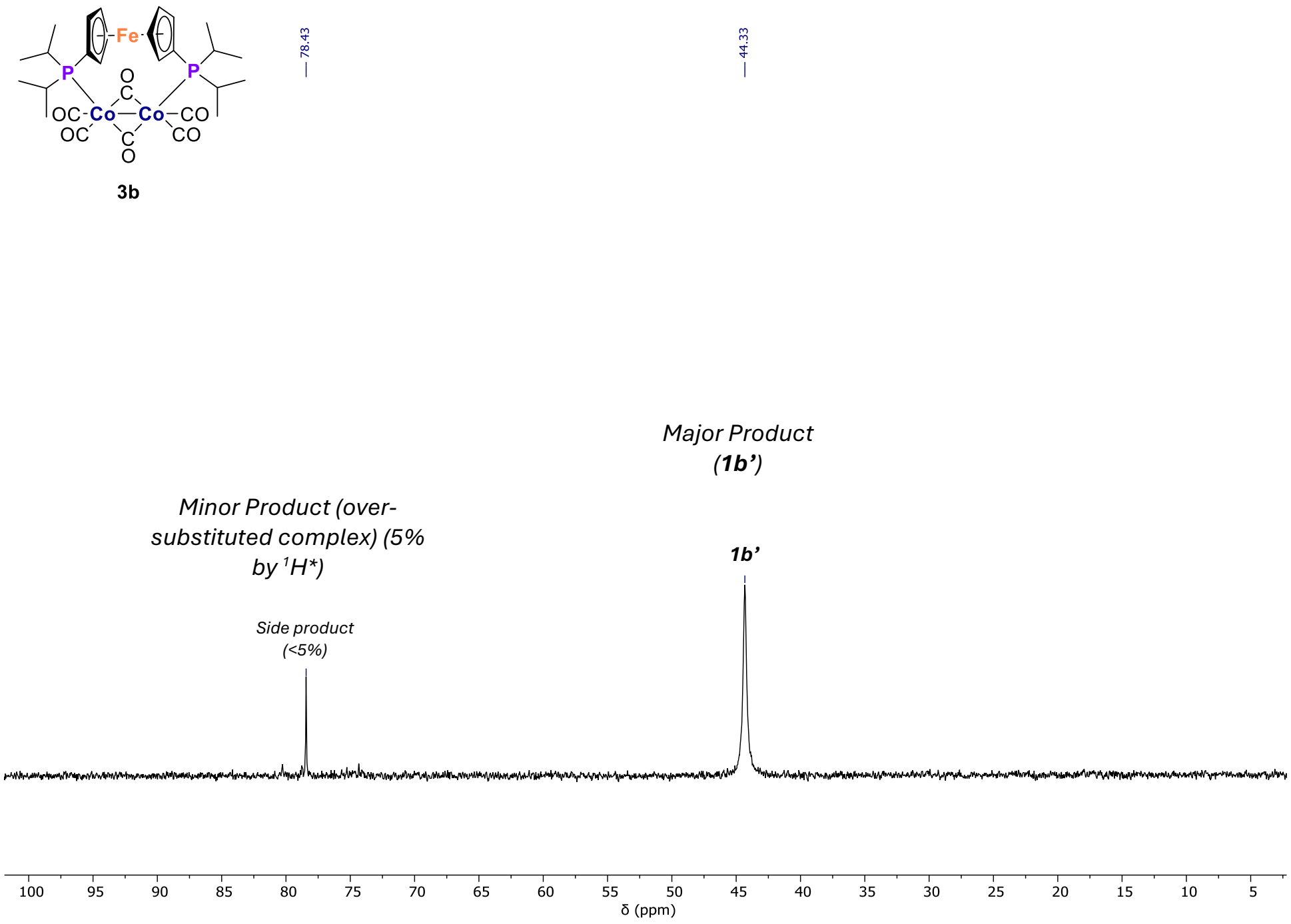

Figure S30. *^{31}P NMR spectra for **3b** (162 MHz, C_6D_6, 298 K). *Higher mass complex confirmed by DOSY experiment. A mass >1000 g mol^{-1} is in alignment with additional equivalents of D^{i}PrPF ligand on complex.*

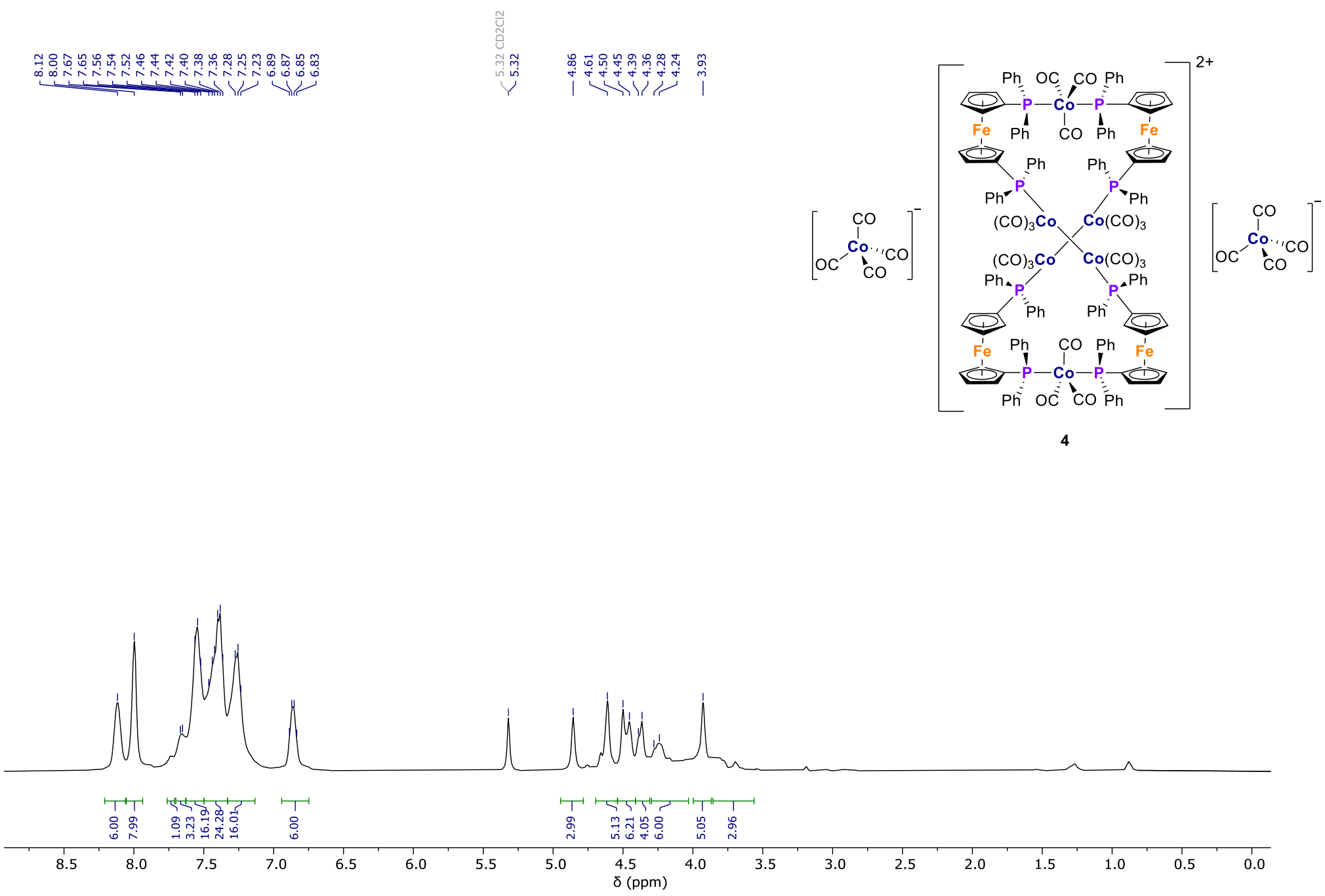

Figure S31. *^{1}H NMR spectra of **4** (400 MHz, CD_2Cl_2, 298 K).*

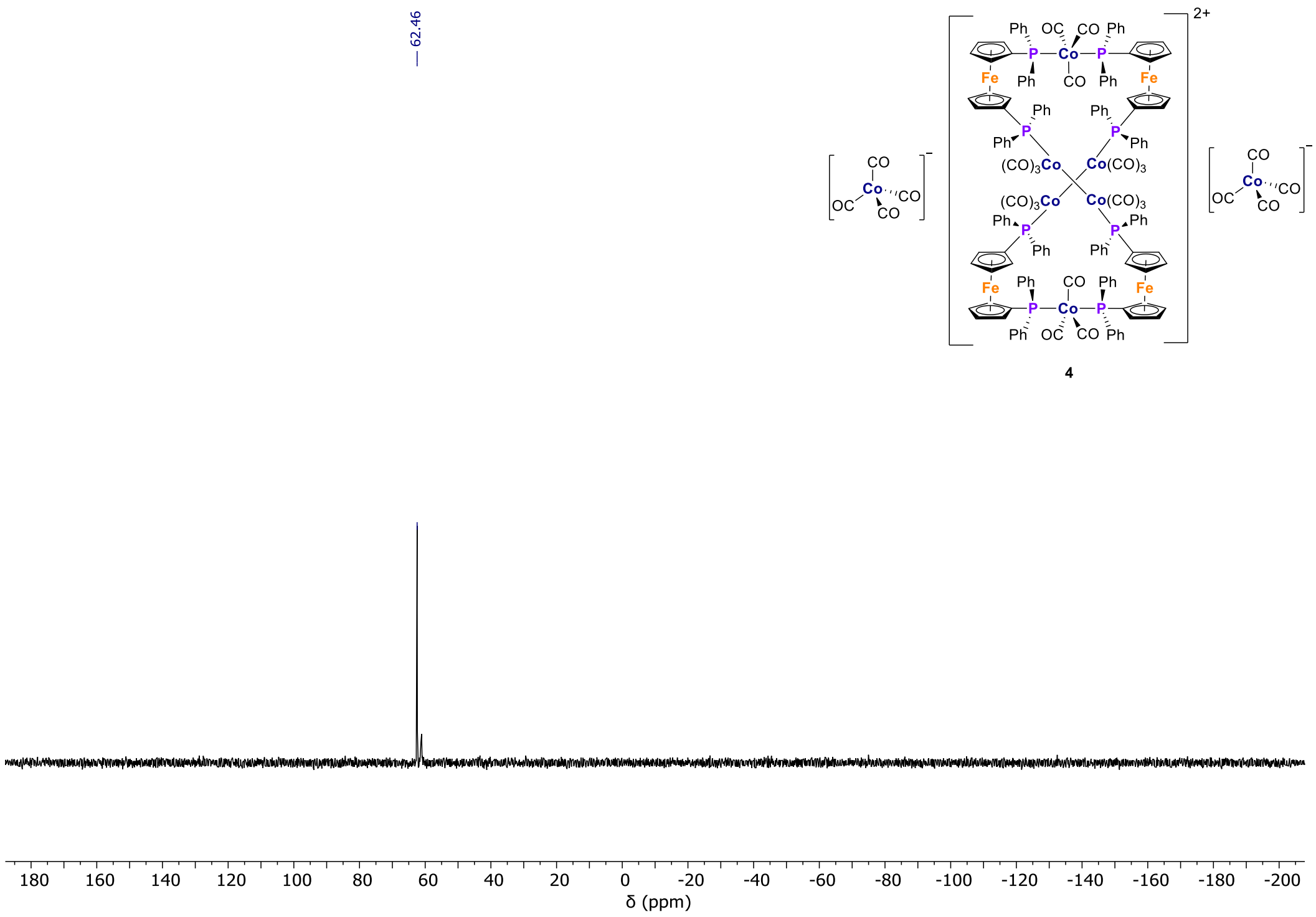

Figure S32. *^{31}P NMR spectra for **4** (162 MHz, CD_2Cl_2, 298 K).*

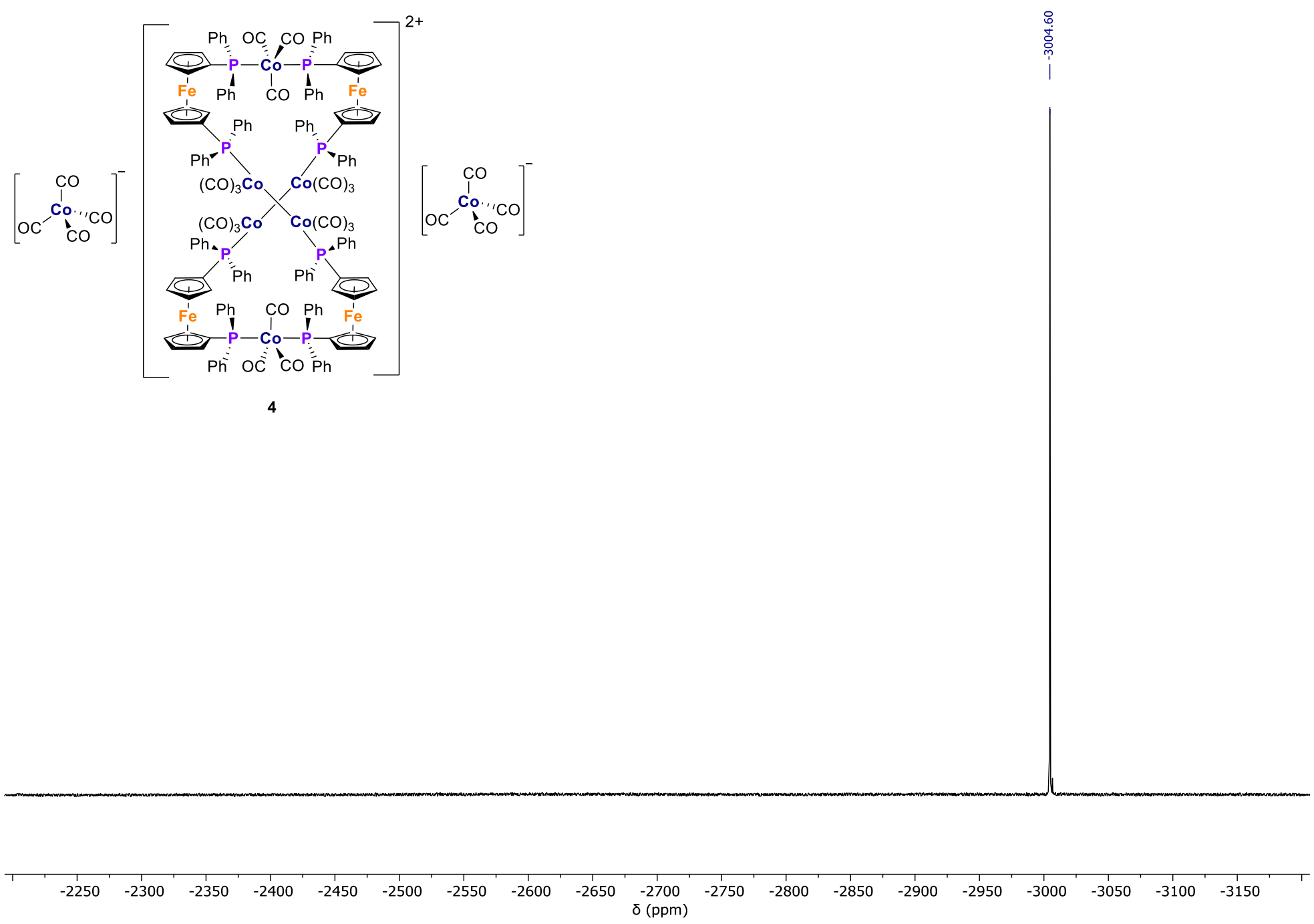

Figure S33. *[59]Co NMR spectra for **4** (94.9 MHz, CD_2Cl_2, 298 K*

DOSY Experiments

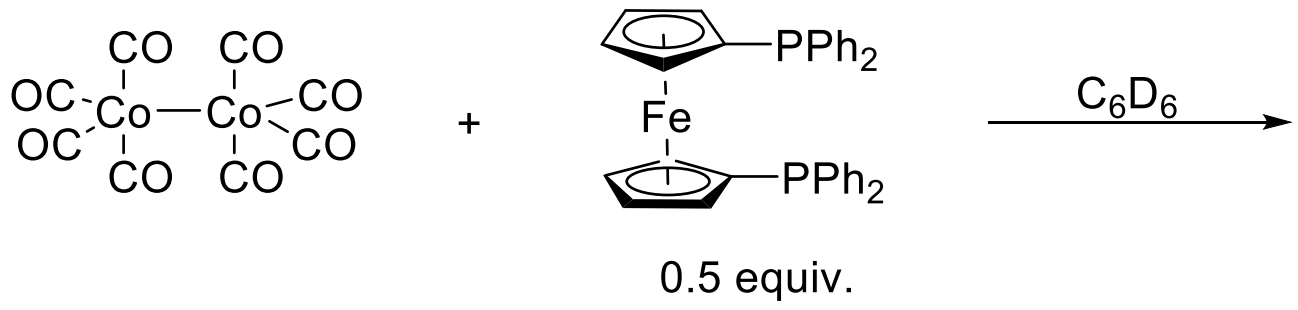

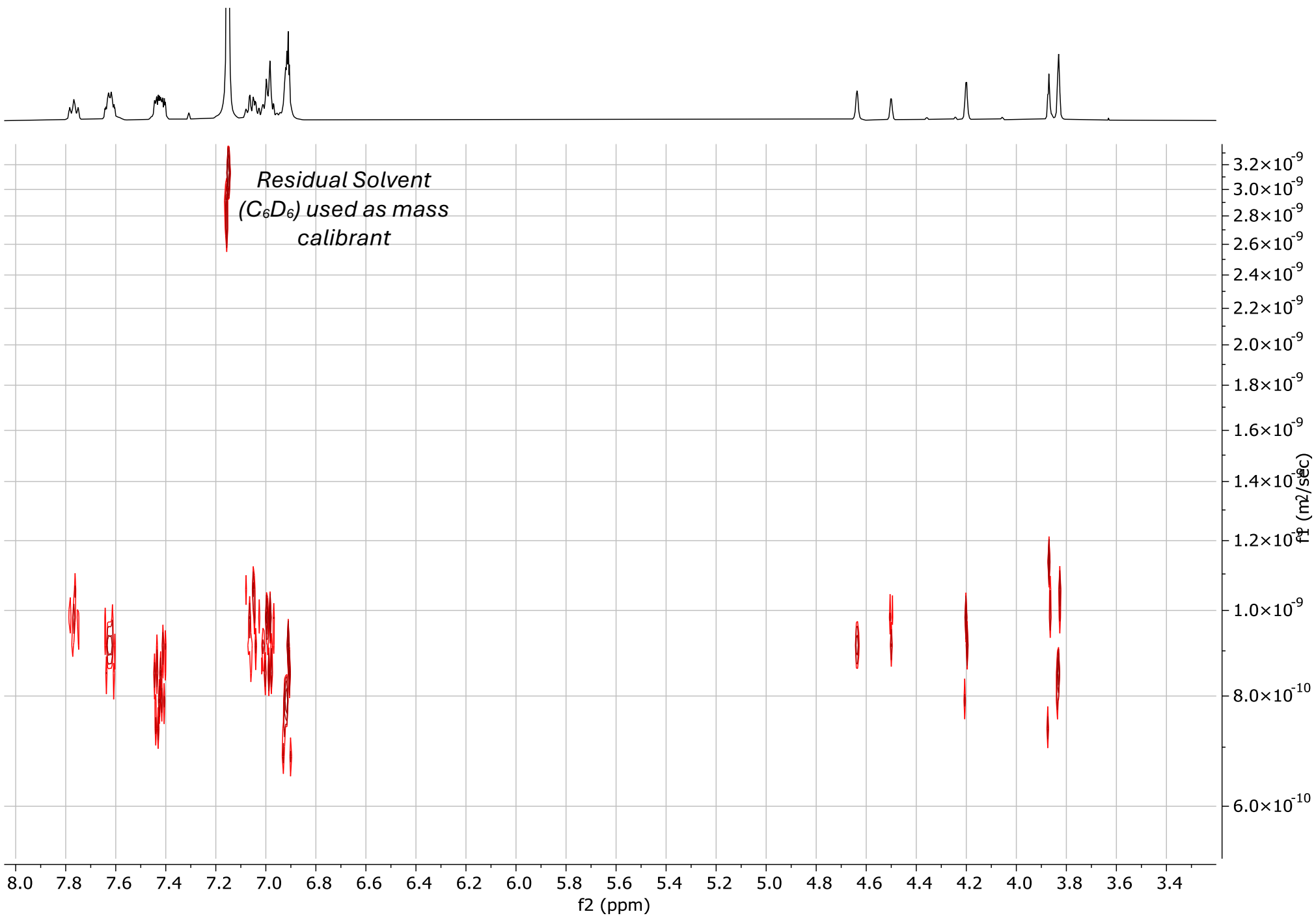

Figure S34. *2D DOSY spectra for mixture of [$Co_2(CO)_8$] (6.8 mg, 0.2 mmol) and DPPF (5.5 mg ,0.1 mmol, 0.5 equiv.) in C_6D_6 (2 mL). Correction versus residual solvent C_6H_6 using Stalke DOSY MW software[1, 2] gives mass range between 709 g mol^{-1} and 1353 g mol^{-1}. The expected mass for the DPPF derivative of **3b** (**3a**) is 840.32 g mol^{-1} (shown).*

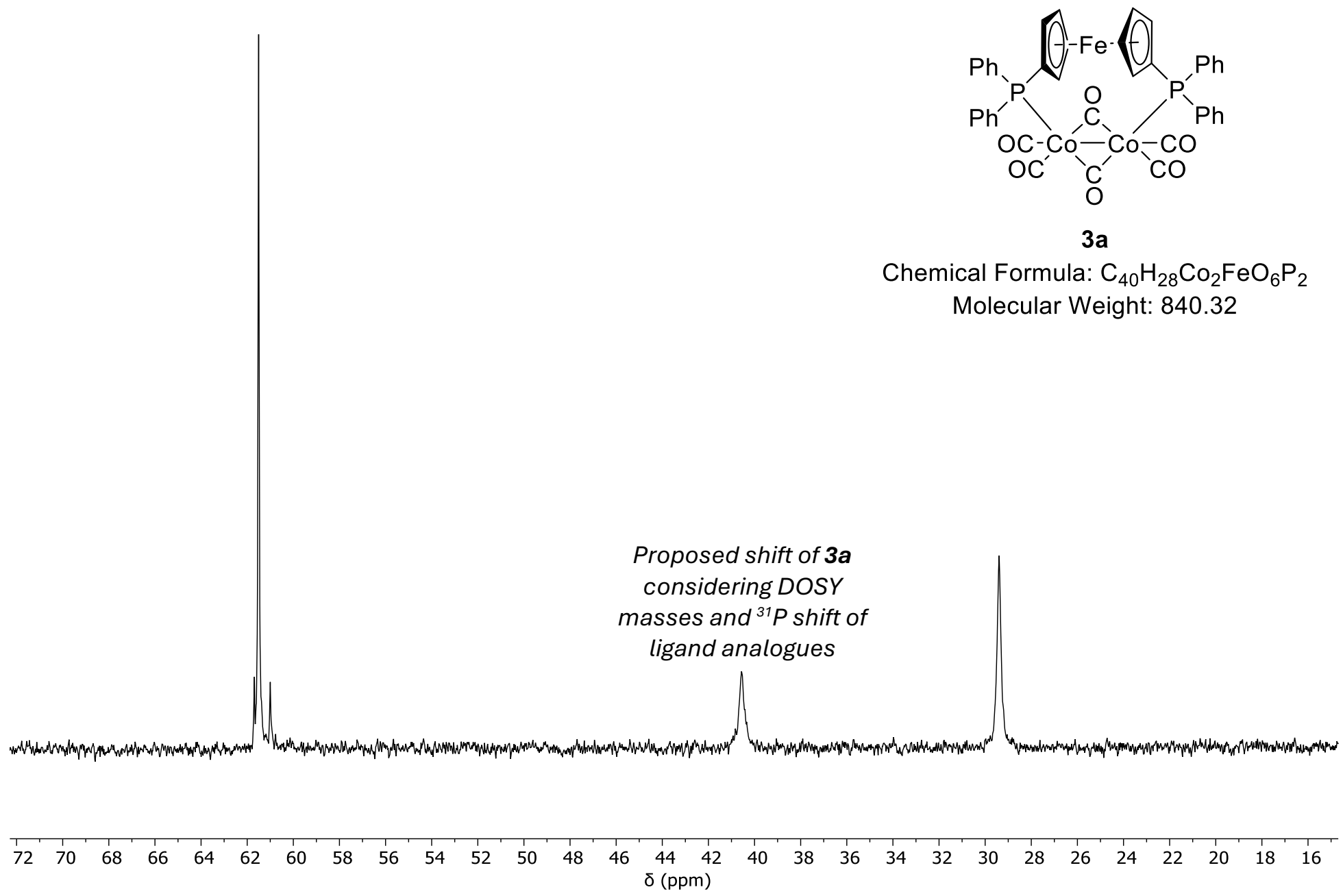

Figure S35. *^{31}P NMR spectra for reaction of [$Co_2(CO)_8$] with DPPF (0.5 equiv.) in C_6D_6.*

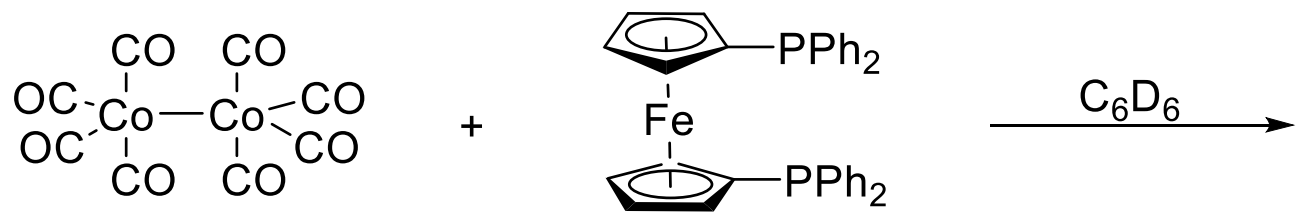

1.0 equiv.

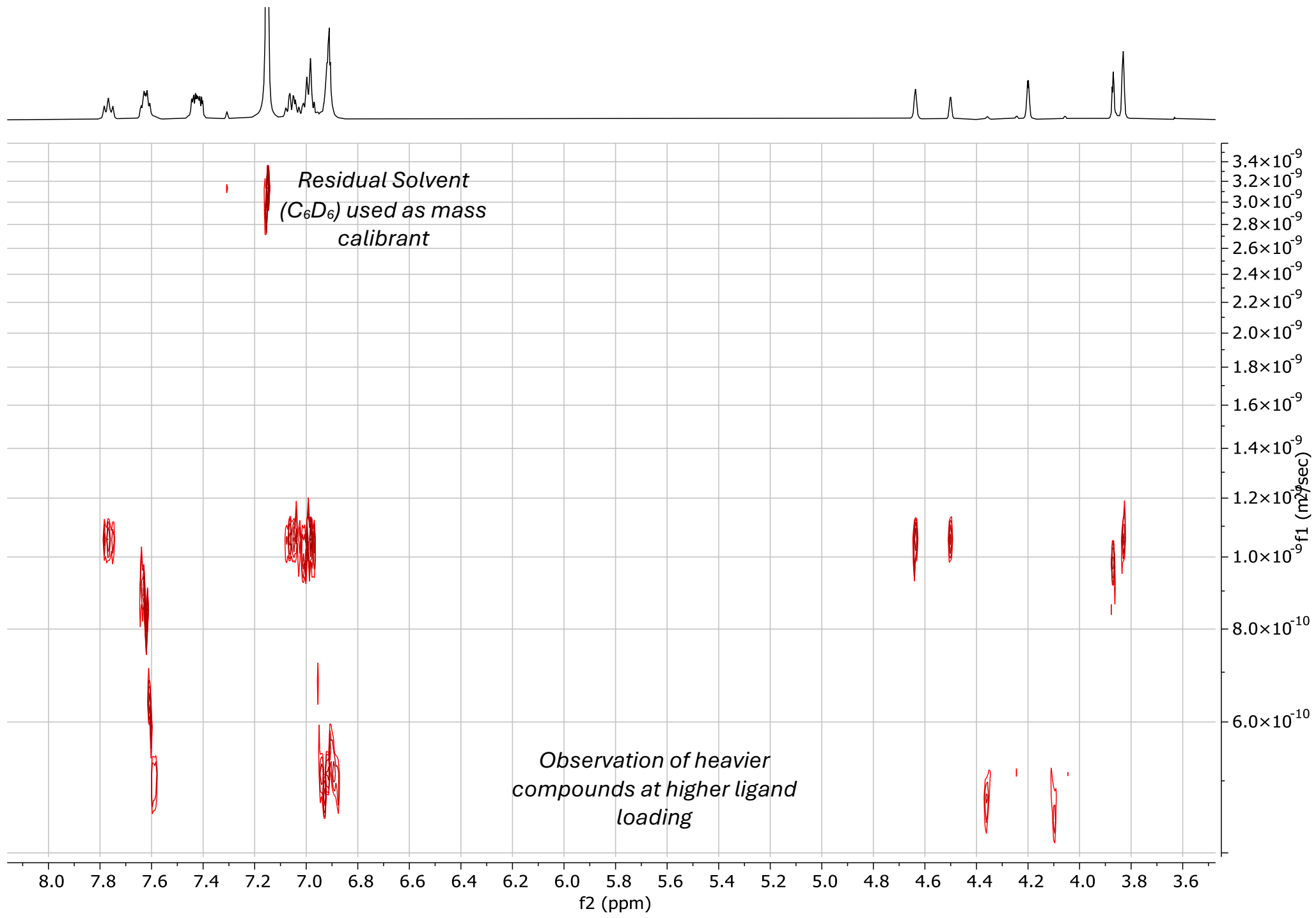

Figure S36. *2D DOSY spectra for mixture of [$Co_2(CO)_8$] (6.8 mg, 0.2 mmol) and DPPF (11 mg ,0.2 mmol,1.0 equiv.) in C_6D_6 (2 mL). Correction versus residual solvent C_6H_6 using Stalke DOSY MW software gives mass range between 728 g mol^{-1} and 4007 g mol^{-1}. The expected mass for the DPPF derivative of **4** is 3417.23 g mol^{-1}. The specific diffusion rate pertaining to a diffusion of 4.96 x10^{-10}m^2 sec^{-1} gives a predicted mass of 3320 g mol^{-1}, suggesting that the large cluster can exist both in solution and the solid phase.*

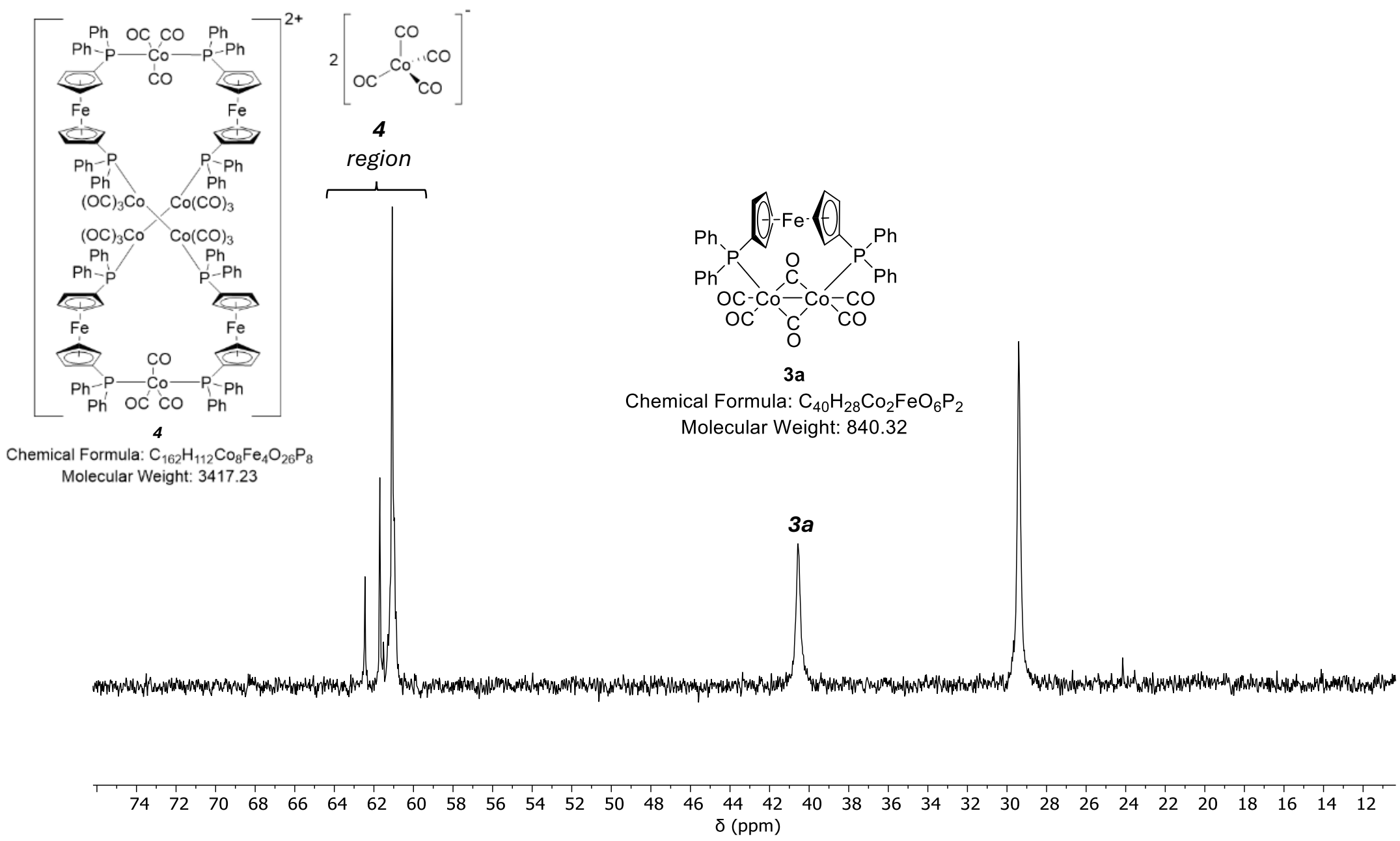

Figure S37. *^{31}P NMR spectra for reaction of [$Co_2(CO)_8$] with DPPF (1.0 equiv.) in C_6D_6.*

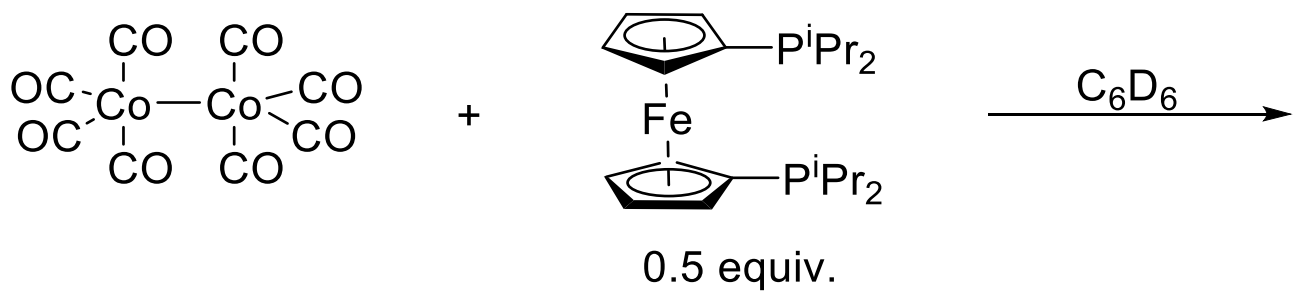

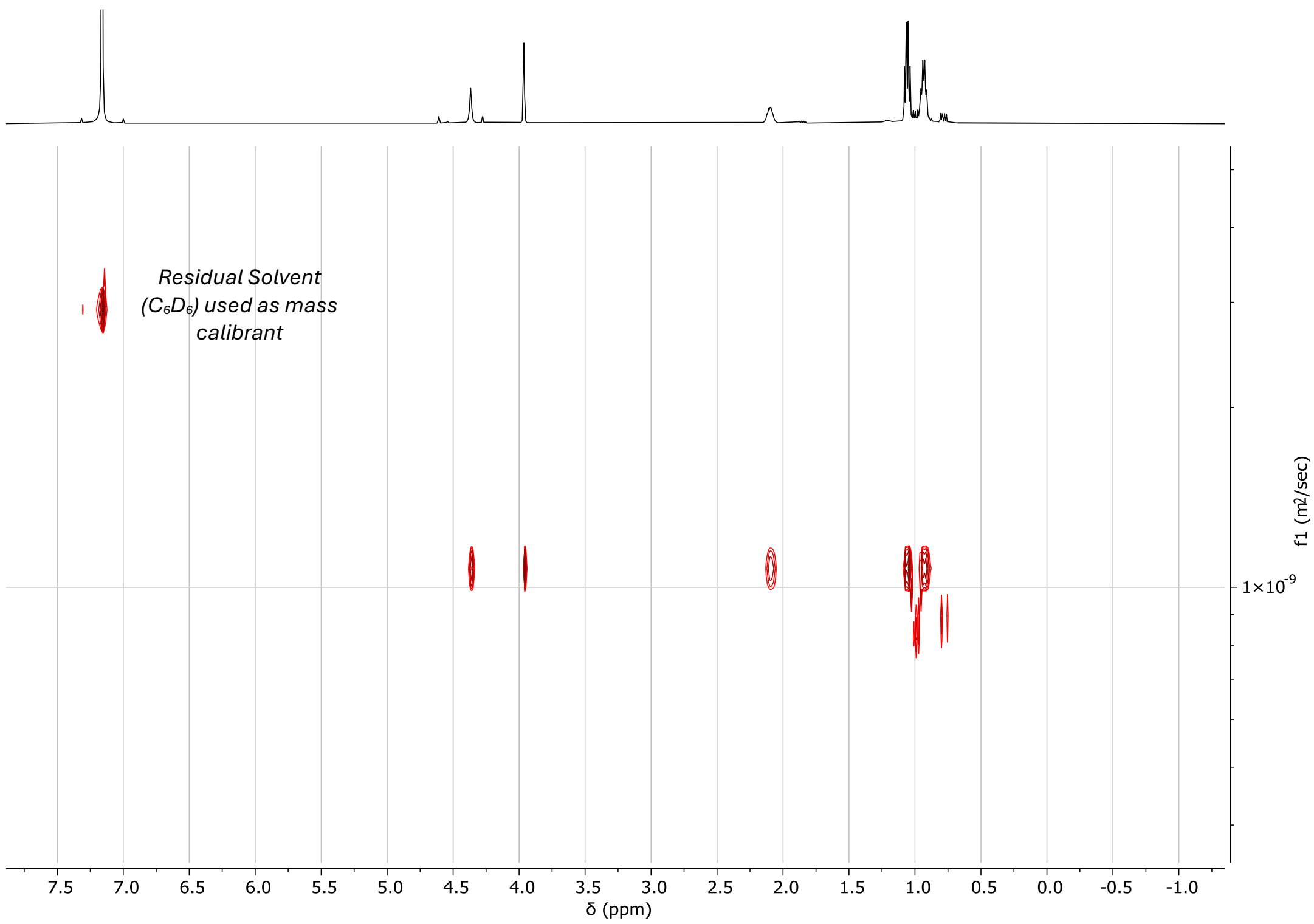

Figure S38. *2D DOSY spectra for **3b** ([(D^{i}PrPF)$Co_2(CO)_6$]). Sample prepared from D^{i}PrPF (4.2 mg, 0.01 mmol, 0.5 equiv.) and [$Co_2(CO)_8$] (6.8 mg, 0.02 mmol) in 2 mL C_6D_6. Correction versus residual solvent C_6H_6 using Stalke DOSY MW software gives mass 669 g mol^{-1} (DOSY) versus 704.25 g mol^{-1} (crystal structure), mass is within expected 10% error for DOSY analysis. Comparable testing of 1:1 mixture D^{i}PrPF and [$Co_2(CO)_8$] gave the same in-solution diffusion coefficients, however notable formation of precipitate was observed in this instance by comparison.*

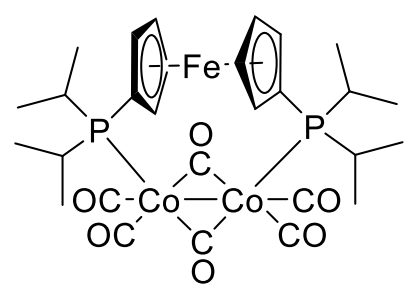

3b
Chemical Formula: $C_{28}H_{36}Co_2FeO_6P_2$
Molecular Weight: 704.25

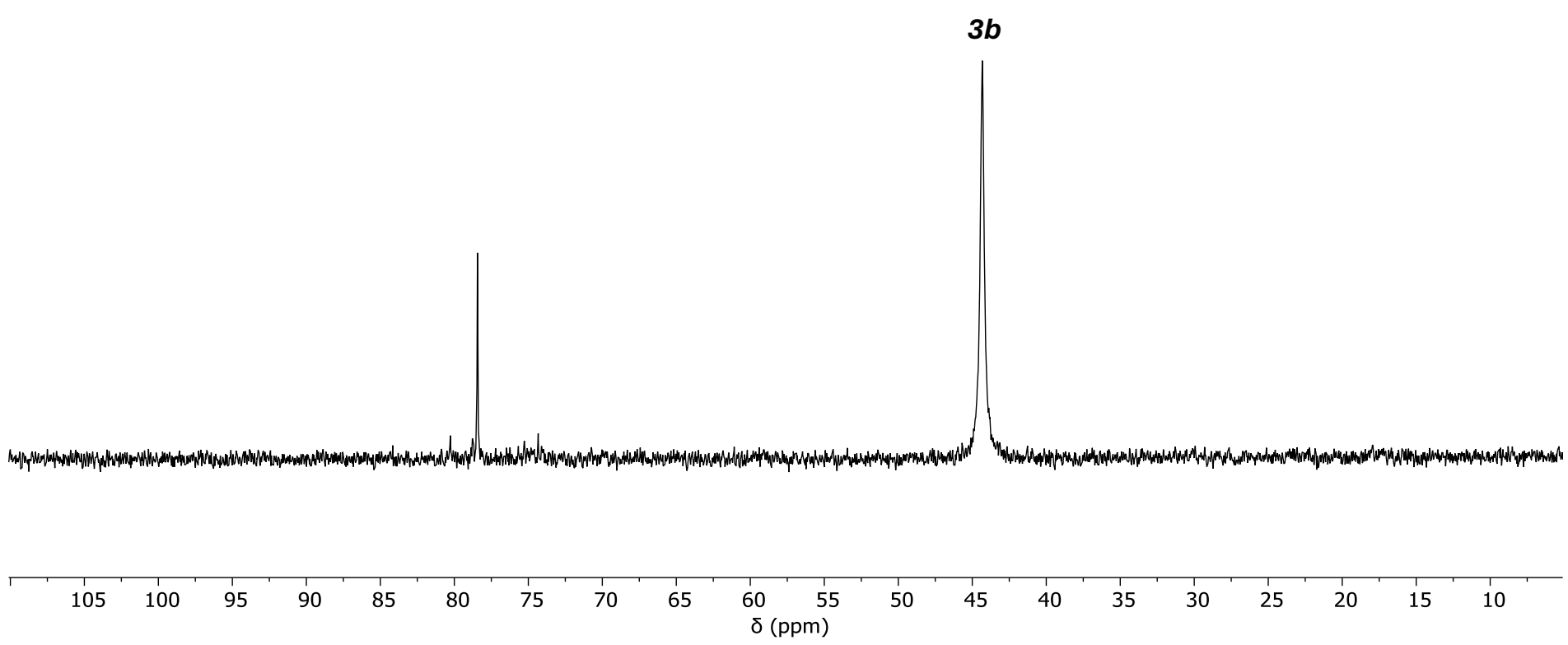

Figure S39. *^{31}P NMR spectra for reaction of [$Co_2(CO)_8$] with D^{i}PrPF (0.5 equiv.) in C_6D_6.*

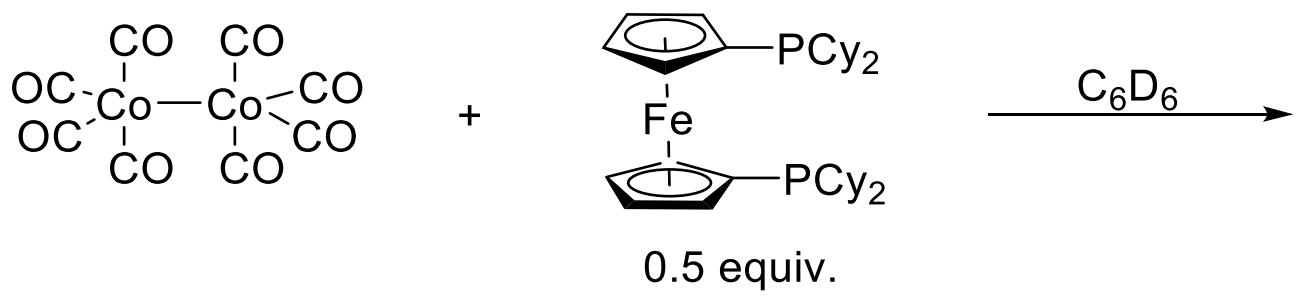

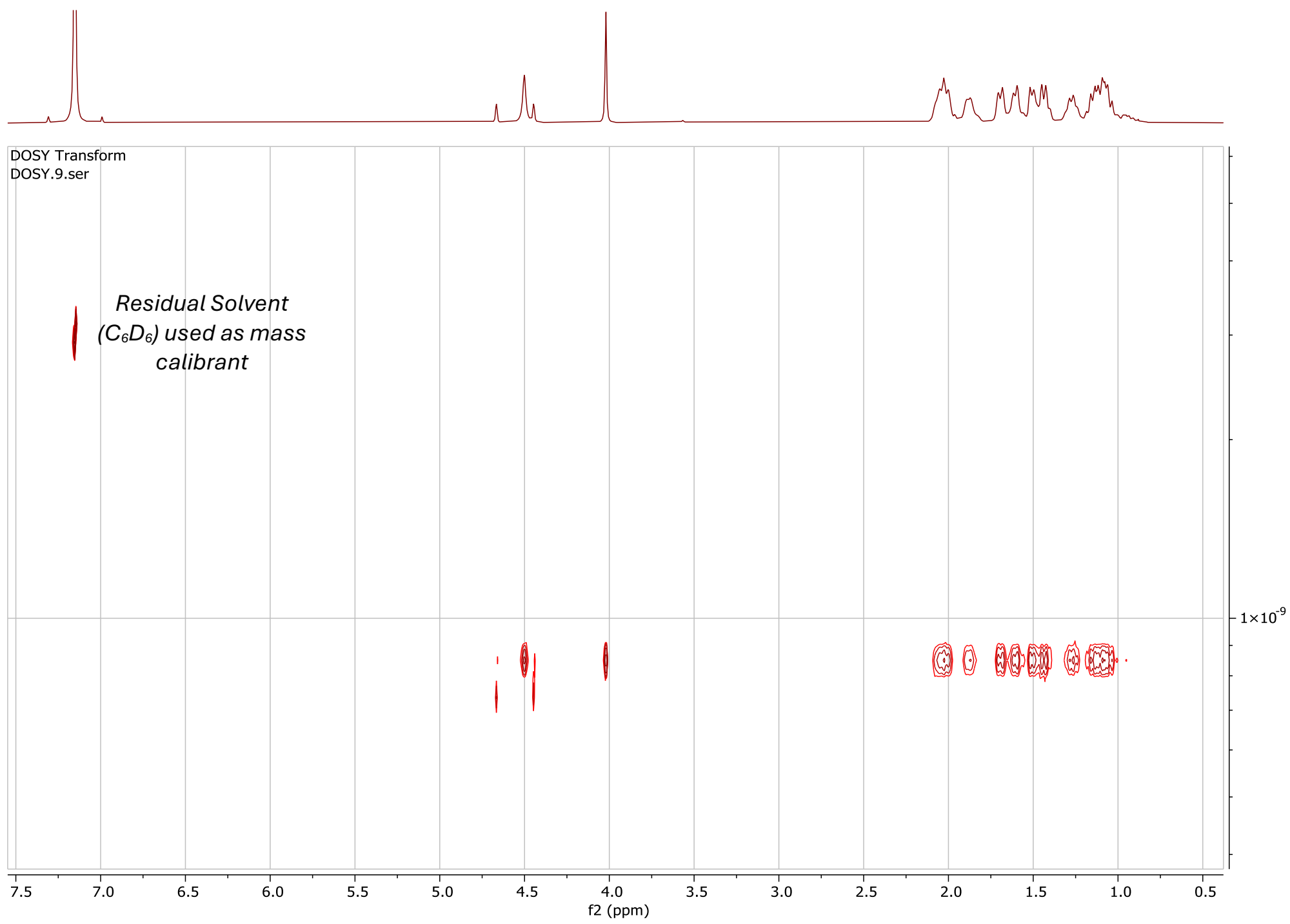

Figure S40. *2D DOSY spectra for mixture of [$Co_2(CO)_8$] (6.8 mg, 0.2 mmol) and DCyPF (5.8 mg ,0.1 mmol, 0.5 equiv.) in C_6D_6 (2 mL). Correction versus residual solvent C_6H_6 using Stalke DOSY MW software gives mass of 906 g mol^{-1} which falls within experimental error expectation. The expected mass for the DCyPF derivative of* ***3b*** *(****3c****) is 864.51 g mol^{-1} (shown)*

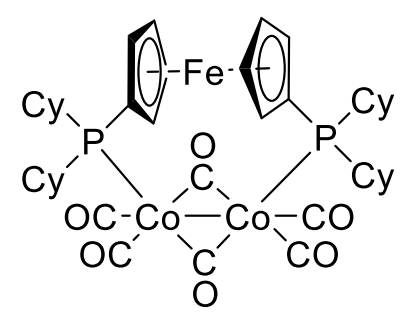

3c

Chemical Formula: $C_{40}H_{52}Co_2FeO_6P_2$

Molecular Weight: 864.51

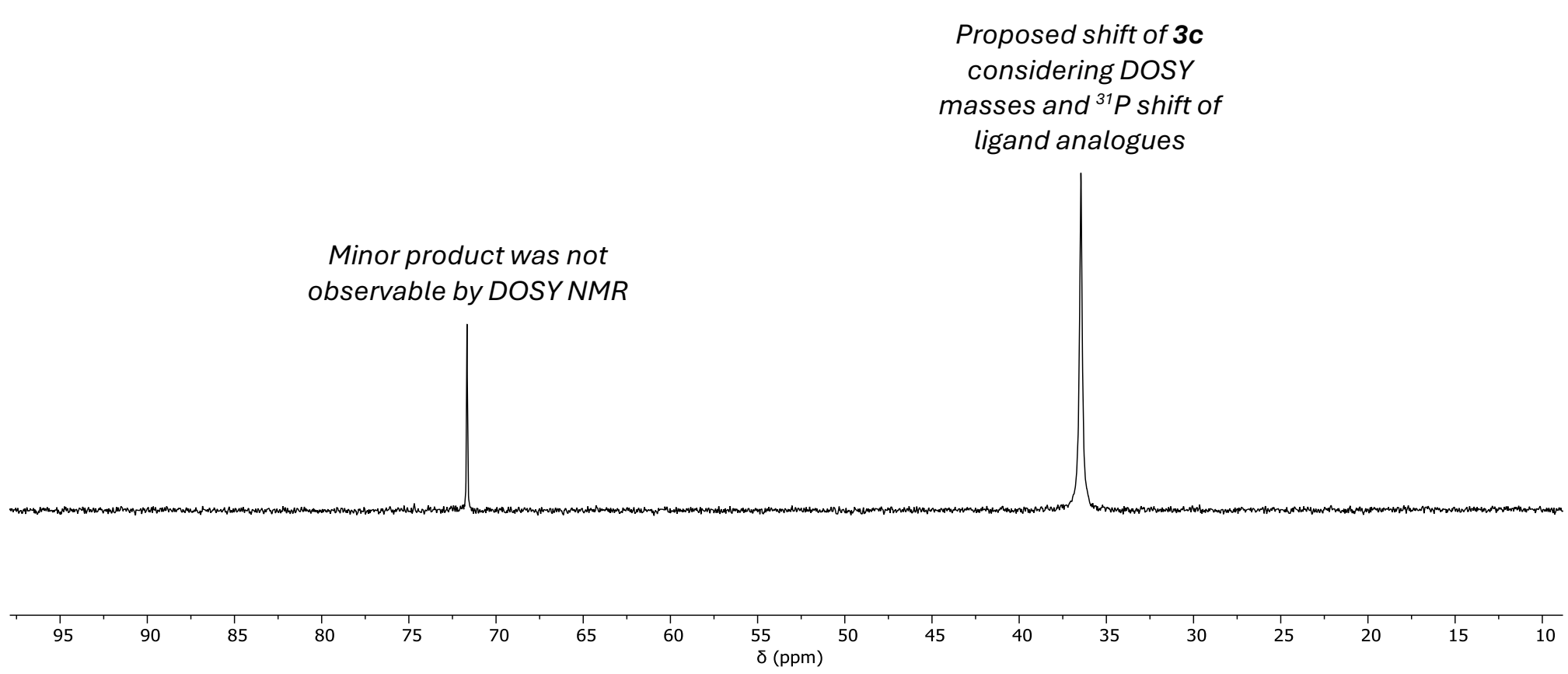

Figure S41. *^{31}P NMR spectra for reaction of [$Co_2(CO)_8$] with DCyPF (0.5 equiv.) in C_6D_6.*

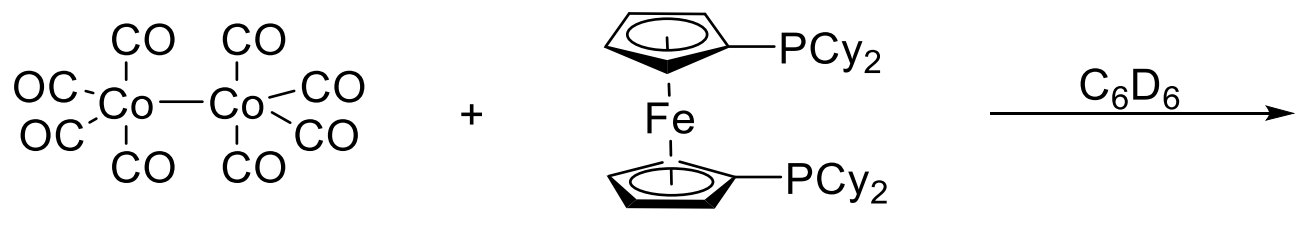

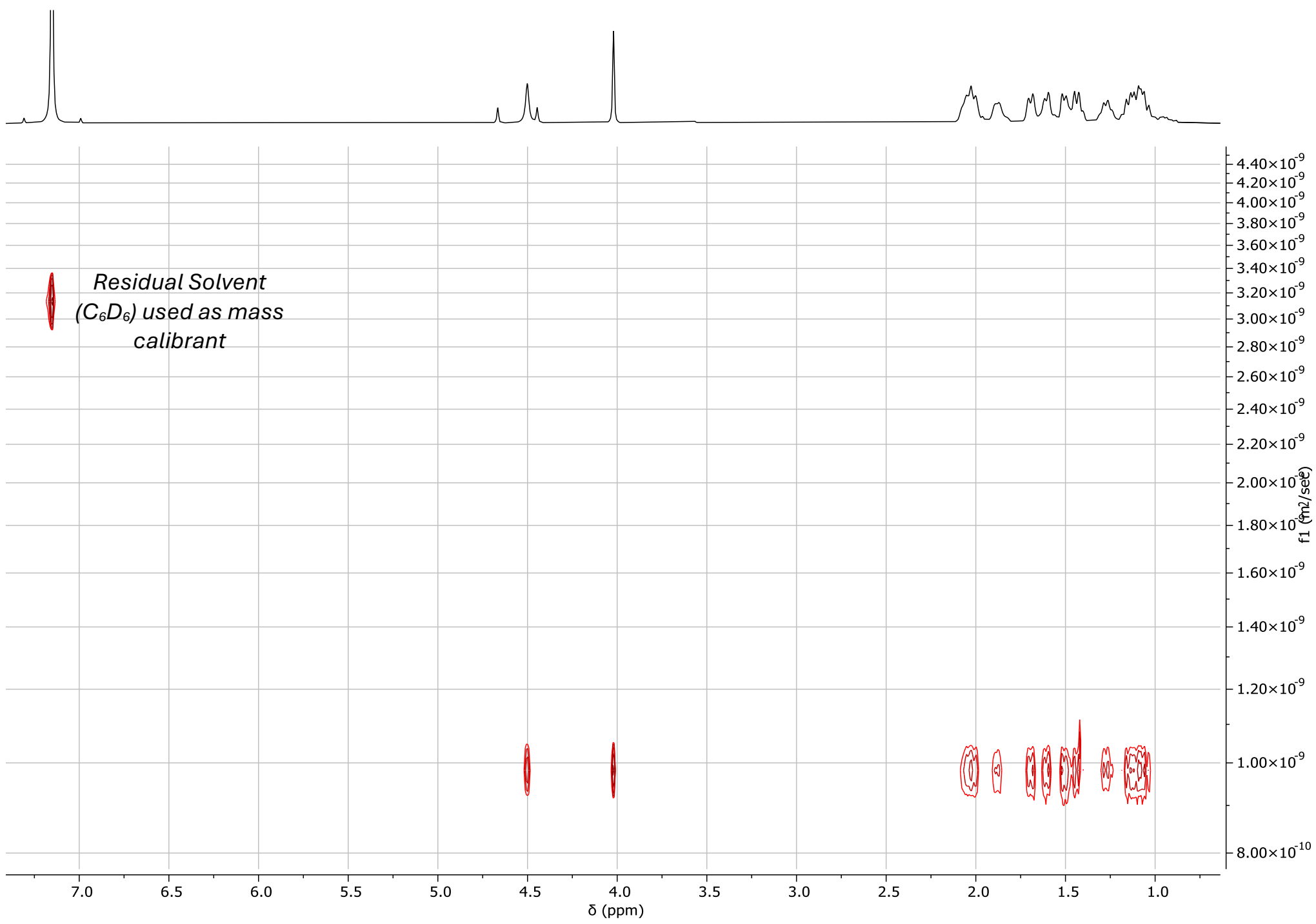

Figure S42. *2D DOSY spectra for mixture of [$Co_2(CO)_8$] (6.8 mg, 0.2 mmol) and DCyPF (5.8 mg ,0.1 mmol, 0.5 equiv.) in C_6D_6 (2 mL). Correction versus residual solvent C_6H_6 using Stalke DOSY MW software gives mass of 835 g mol^{-1}which falls within experimental error expectation. The expected mass for the DCyPF derivative of* ***3b*** *(****3c****) is 864.51 g mol^{-1}.*

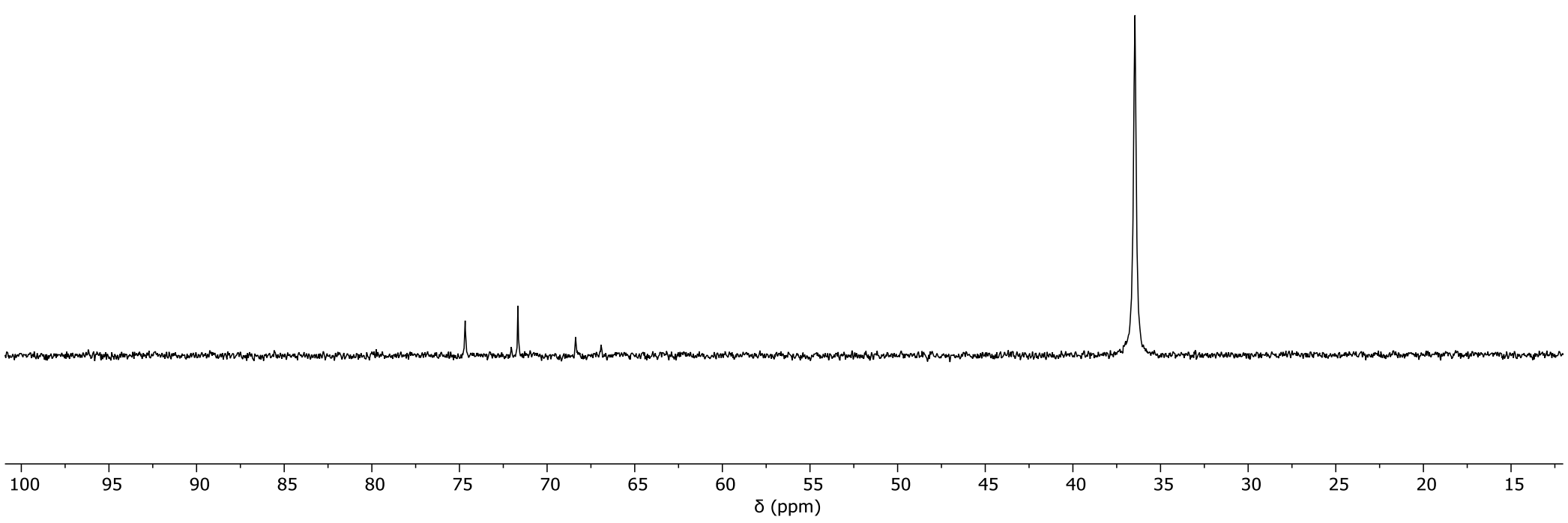

Figure S43. *^{31}P NMR spectra for reaction of [$Co_2(CO)_8$] with DCyPF (1.0 equiv.) in C_6D_6.*

Single Crystal X-ray Diffraction Data

data	1a	3b	4
**CCDC number**	2528291	2528292	2528290
**formula**	$[C_{29}H_{48}CoO_3P_2][C_4CoO_4]$	$C_{28}H_{36}Co_2FeO_6P_2$	$[C_{154}H_{112}Co_6Fe_4O_{18}P_8]$ $2[Co(CO)_4]_2$, $5.5[CH_2Cl_2]$, $2.75[C_5H_{12}]$
**formula weight (g·mol⁻¹)**	736.51	704.22	4082.60
**colour, habit**	pale yellow needle	orange block	red block
**crystal size (mm)**	0.304 x 0.188 x 0.076	0.29 x 0.2 x 0.07	0.593 x 0.465 x 0.158
**crystal system**	monoclinic	orthorhombic	monoclinic
**space group**	$P2_1/c$ (no. 14)	*Pbca* (no. 61)	I2/a (no. 15)
a **(Å)**	27.6311(8)	9.70670(10)	26.2077(2)
b **(Å)**	12.0282(4)	14.98700(10)	19.08744(17)
c **(Å)**	22.2562(6)	40.7576(3)	34.9761(3)
α **(°)**	90	90	90
β **(°)**	108.915(3)	90	97.8466(9)
γ **(°)**	90	90	90
**V ($Å^3$)**	6997.5(4)	5929.18(8)	17332.6(3)
**Z**	8 [c]	8 [b]	4
**T (K)**	172.95(10)	173(2)	173.00(14)
**D_c ($g·cm^{-3}$)**	1.400	1.578	1.565
**radiation used, μ (mm^{-1})**	Mo Kα, 1.083	Cu Kα, 13.887	Mo Kα, 1.380
**F(000)**	8296	2896	8314
**absorption correction**	analytical	analytical	analytical
**min-max transmission**	0.960 - 0.988	0.055 - 0.711	0.551 - 0.791
**Θ range for data collection (°)**	2.292 - 28.308	4.339 - 68.319	2.375 - 28.341
**no. of unique reflns measured, obs [F > 4σ(F)]**	15608, 10415	5427, 5076	19448, 16434
**R_{int}, R_{sigma}**	0.0596, 0.0578	0.0529, 0.0224	0.0291, 0.0182
**completeness to Θ(full) (°)**	0.999 to 25.242	1.000 to 67.684	0.998 to 25.242
**no. of parameters, restraints**	818, 943	360, 0	974, 221
**R_1, wR_2 [F > 4σ(F)] [a]**	0.0702, 0.1427	0.0257, 0.0638	0.0350, 0.0966
**R_1, wR_2 [all data] [a]**	0.1183, 0.1612	0.0281, 0.0650	0.0453, 0.1048
**GooF**	1.140	1.035	1.097
**largest diff. Fourier peak, hole ($eÅ^{-3}$)**	0.929, -0.371	0.304, -0.319	0.785, -1.098

Table S1. *Crystal Data, Data Collection and Refinement Parameters. Data were collected using Rigaku XtaLAB Synergy-i (Cu radiation, compound* ***3b****) and Agilent Xcalibur 3 E Agilent (Mo radiation, compounds* ***1a*** *and 4) diffractometers. Raw frame data was reduced using CrysAlisPro,[3] and the structures were solved and refined using the OLEX2,[4] SHELXT,[5] and SHELX-2018[6] program systems.* [a] $R_1 = \Sigma||F_o| - |F_c||/\Sigma|F_o|$; $wR_2 = \{\Sigma[w(F_o^2 - F_c^2)^2] / \Sigma[w(F_o^2)^2]\}^{1/2}$; $w^{-1} = \sigma_2(F_o^2) + (aP)^2 + bP$. [b] *The asymmetric unit contains two crystallographically independent molecules.* [c] *The asymmetric unit contains half of the main molecule.*

The X-ray crystal structure of **1a**

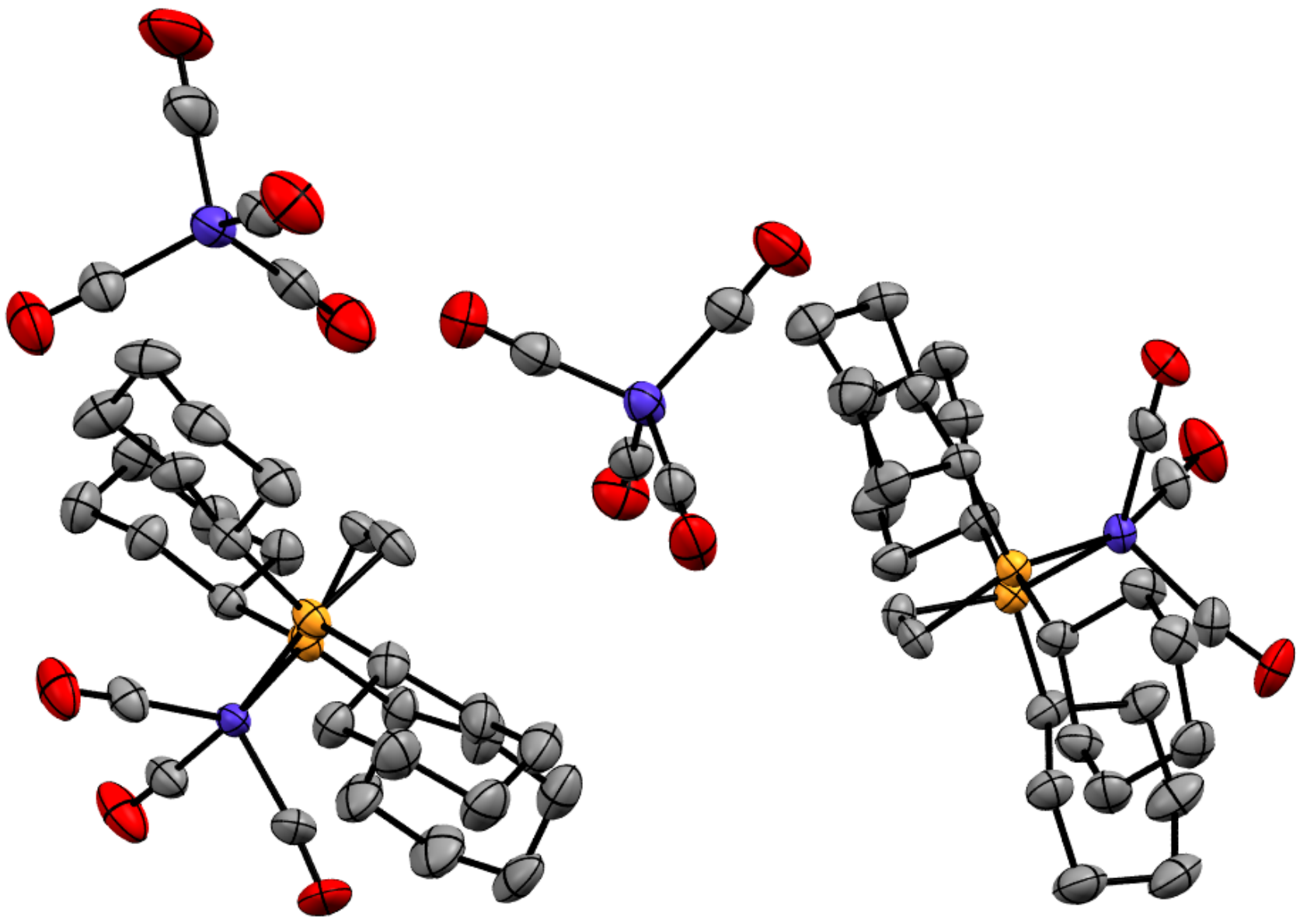

Figure S44. *X-ray structure of **1a**, including both independent main molecules and counterions. Hydrogen atoms and the minor occupancy orientation are hidden for clarity.*

The ASU for **1a** was found to contain two crystallographically independent molecules of the main molecule as well as 2 $[Co(CO)_4]^-$ counterions, all in general positions. The C28-based cyclohexyl group was found to be disordered. It was modelled in two orientations at a ratio of ca. 0.74:0.26. The two orientations were restrained to be similar and only the non-hydrogen atoms of the major orientations were modelled anisotropically, the rest were modelled isotropically.

The X-ray crystal structure of **3b**

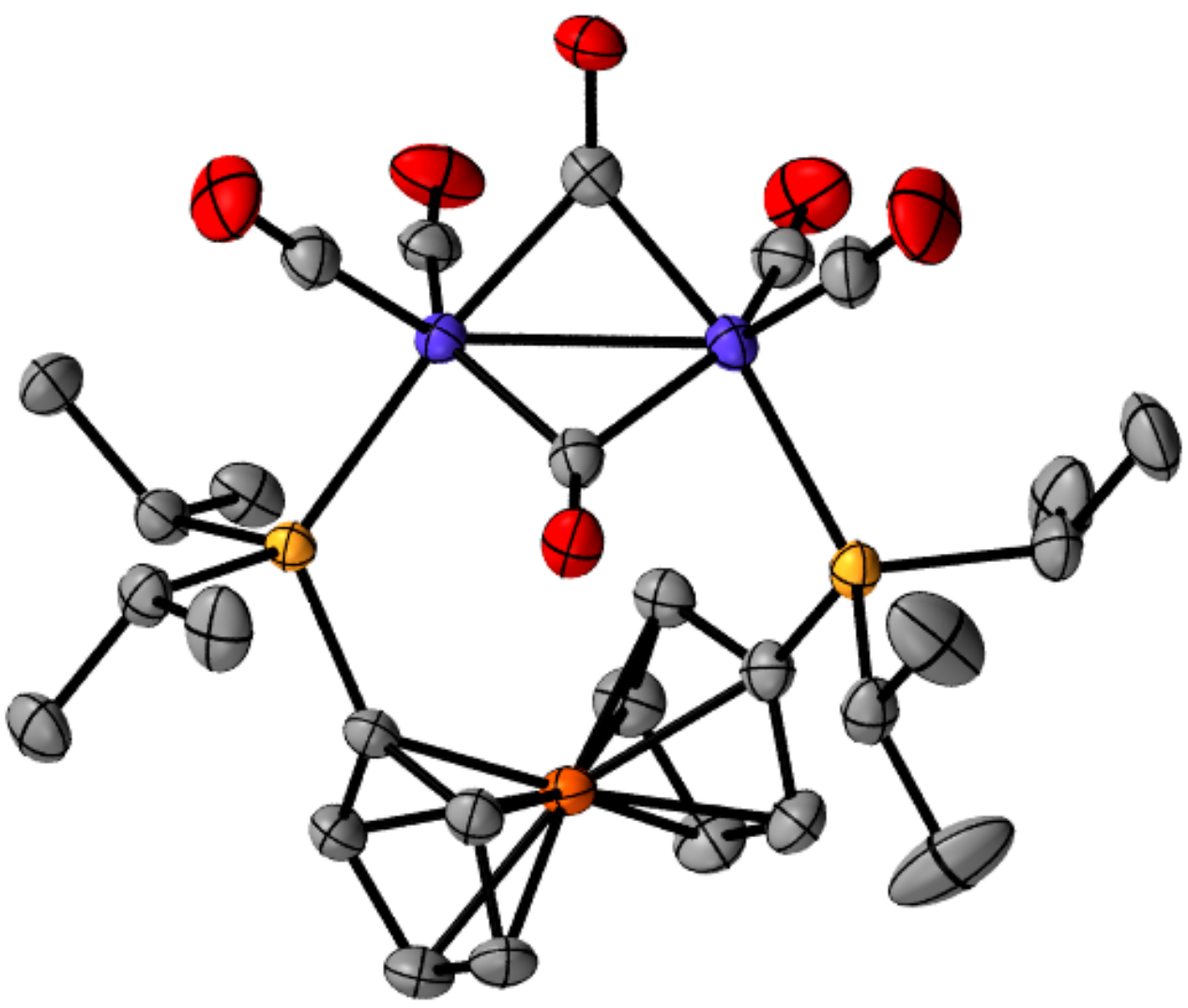

Figure S45. *X-ray structure of **3b**. Hydrogen atoms and atoms are hidden for clarity.*

PLAT230_ALERT_2_B: Analysis of the structure indicates that the observed differences in anisotropic displacement parameters along the C O bond arise from the nature of the metal carbonyl bonding and are not due to disorder or incorrect atom assignment. See *Acta. Cryst.* **2009** D65 148 155.

The X-ray crystal structure of **4**

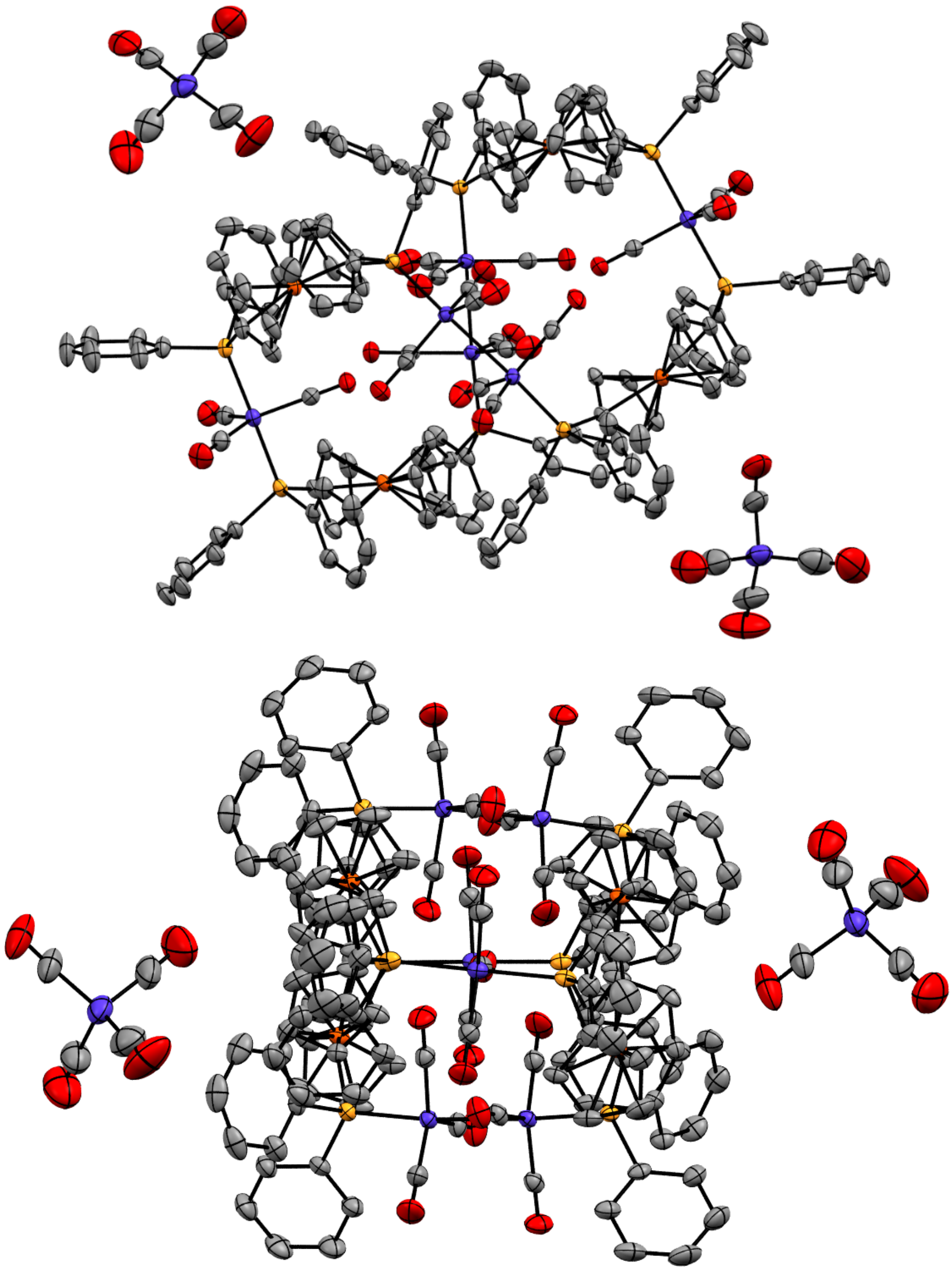

Figure S46. *X-ray structure of **4**, top/bottom: two different views. Hydrogen atoms are hidden for clarity.*

The crystal was found to be twinned, but the best model was obtained by not modelling the twinning. The main molecule macrocycle of **4** is located on a crystallographic C_2 axis, ca. 1.79 Å from the closest atom, O8; this means ASU contains half of the macrocycle. The ASU was also found to contain 1 $[Co(CO)_4]^-$ counterion (2 per macrocycle) as well as several solvent molecules. The $[Co(CO)_4]^-$ counterion was found to be disordered. It was modelled in two orientations at a ratio of ca. 0.83:0.17. The two orientations were restrained to be similar and only the atoms of the major occupancy orientation were modelled anisotropically, the rest were modelled isotropically. The solvent molecules were found to exhibit heavy disorder and could not be modelled satisfactorily. Initially, the solvent molecules looked like the solvents used in crystallisation, a mixture of DCM and pentane in ca. 2:1 ratio. The SQUEEZE[7] routine

implemented in PLATON[8,9] was used to mask these: overall 1 void of 3767 Å^3 was found per unit cell containing 1390 electrons. This is consistent with the presence of 5.5 molecules of DCM and 2.75 molecules of pentane per ASU, corresponding to 1386 electron per unit cell.

PLAT910_ALERT_3_B: Due to the large unit cell and the Mo radiation used, a large number of reflections are obscured by the beamstop.

References

(1) Bachmann, S.; Gernert, B.; Stalke, D. Solution structures of alkali metal cyclopentadienides in THF estimated by ECC-DOSY NMR-spectroscopy (incl. software). *Chem. Comm.,* **2016**, *52* (87), 12861-12864,
(2) Neufeld, R.; Stalke, D. Accurate molecular weight determination of small molecules via DOSY-NMR by using external calibration curves with normalized diffusion coefficients. *Chem. Sci.,* **2015**, *6* (6), 3354-3364,
(3) Agilent (**2014**). CrysAlis PRO. Agilent Technologies Ltd, Yarnton, Oxfordshire, England.
(4) Dolomanov, O. V., Bourhis, L. J., Gildea, R. J., Howard, J. A. K., Puschmann, H., OLEX2 : a complete structure solution, refinement and analysis program *J. Appl. Crystallogr.*, **2009**, 42, 339–341.
(5) Sheldrick, G. M., SHELXT – Integrated space-group and crystal-structure determination Acta *Crystallogr. Sect. A Found. Adv.,* **2015**, 71, 3–8.
(6) Sheldrick, G. M., Crystal structure refinement with SHELXL Acta Crystallogr. Sect. C Struct. Chem. **2015**, 71, 3–8.
(7) P. van der Sluis; Spek, A. L. BYPASS: an effective method for the refinement of crystal structures containing disordered solvent regions. *Acta Crystallogr. Sect. A,* **1990**, *46* (3), 194-201
(8) Spek, A. L. PLATON. A Multipurpose Crystallographic Tool, Utrecht University, The Netherlands, 1998.
(9) Spek, A. L. Single-crystal structure validation with the program PLATON. *J. Appl. Crystallogr.,* **2003**, *36* (1), 7-13,
